# Non-coding RNAs match the deleted genomic regions in humans

**DOI:** 10.1038/srep37452

**Published:** 2016-11-17

**Authors:** Boseon Byeon, Igor Kovalchuk

**Affiliations:** 1 Department of Biological Sciences, University of Lethbridge, Lethbridge, T1K 3M4, AB, Canada

## Abstract

RNA is transcribed from DNA, and therefore, there should be no RNA transcript from the deleted DNA region. Our study attempted to analyse whether any RNA cache that maps the deleted regions is present in human cells. Using data from the 1000 genome project, we selected 41 CEPH (CEU) and 38 Yoruba (YRI) samples that included the data for the entire genome sequence and ncRNA and mRNA sequences. Aligning the ncRNA reads against the genomic DNA in individual samples has revealed that 229 out of 1114 homozygous deletions have ncRNA reads that map to them. Further analysis has revealed that ncRNA reads that map the deleted regions are enriched around the deletion ends and at genic regions of the genome. The read enrichment at deletion ends suggests that these ncRNAs are likely some form of double-strand break induced RNAs. Our analysis suggests that human cells may contain a residual ncRNA cache that is possibly propagated across generations.

DNA is the primary source of heritable information. However, with the advent of epigenetics, it became apparent that modifications of DNA and the associated histones can also be heritable and affect the progeny^1^,^2^. RNA may also contain genetic material, as in RNA viruses. In the past two decades, research indicates a critical role of non-coding RNAs (ncRNAs) in the regulation of gene expression at multiple transcriptional and posttranscriptional levels^3^,^4^. In some species, ncRNAs are essential even for targeted rearrangements of genomic DNA, as seen in ciliates^5^. In *Oxytricha*, maternal RNA templates function as a guide for the elimination of massive amounts of DNA fragments by deleting ~95% of the germline genome, resulting in the fragmentation and reassortment of the remaining genome^6^. These ncRNAs also function as templates for fixing DNA fragments that are incorrectly spliced or mutated and can also propagate mutations accumulated during maternal growth^6^.

Mutations are typically restored to original sequence only via random reversion events. Mutations are rare, about 10^−8^ per nucleotide per generation^7^, and the chance of single-nucleotide mutation reverting back is at least 10-fold lower^8^. That is why the report by Lolle *et al.*^9^, which demonstrated the ~10% reversion frequency in *Arabidopsis* plants containing a homozygous recessive mutation in *HOTHEAD (HTH*), was so puzzling^9^. The authors suggested that RNA was a template for restoring the DNA sequence. Other explanations of this phenomenon included an alternative model for the distributed genome in which *HTH* mutations were reversed by homologous sequences present in the genome itself^10^ or by recombining with DNA fragments inherited from a previous generation^11^. The unusual susceptibility of this mutant to outcrossing was proposed^12^ and partially confirmed^13^ as one of other possible mechanisms.

Several key questions about the potential role of RNA in the process of inheritance remain unanswered. What happens to the mRNA and ncRNA cache when certain genomic regions transition from a heterozygous to homozygous genomic deletion that eliminates the potential transcripts? Cells in the developing organism may likely contain a certain number of residual mRNA or ncRNA molecules stemming from the region before a deletion event. If such events occur in diploid germ cells before meiosis, the molecules can end up in haploid sperm or ovum cells lacking certain genomic regions. How quickly are such molecules degraded? Would they survive over a single generation or multiple generations? These questions are absolutely critical for understanding the potential roles RNA molecules can play in the inheritance of genetic traits and even in partial or complete restoration of deleted genomic regions. Unfortunately, at the time of writing this manuscript, no such information exists for animals or plants.

In the current study, we have attempted to analyse the distribution of ncRNAs across the genome in two human populations. We found the presence of multiple ncRNAs reads matching various deleted genomic regions. We have also found that these reads are enriched at the deletion ends, at genic regions, and specifically at exons.

## Results

### ncRNA reads mapped to deletions

The total number of deletions found in 41 CEU and 38 Yoruba samples was 1114, with 467 unique deletions. The CEU population had 231 unique deletions, whereas Yoruba population–236 (File S1). Among the unique deletions, 239 deletions were unique to specific individuals, whereas the remaining 228 deletions were found in two or more individuals. Deletions ranged from 96 nt to 690,380 nt and were 21,538 ± 50,772 on average (File S1). Most deletions were unique to either the CEU or Yoruba population, and 14 different deletions appeared in ≥25% of the population (10 or more individuals) (Table 1). Ten deletions were common between two populations and were present at least in one individual from each population. Deletions at some chromosomes at chromosomal regions were more common, such as deletions at ch3, ch4 and ch7. Moreover, ch3 and ch7 had deletions with the same start (5′ end) but different ends (3′ end) for different populations, resulting in deletions of different size (Table 1).

Mapping ncRNA sequences in each individual sample to the corresponding genomic sequences revealed that ncRNAs mapped to 229 out of 1114 deletions, with 137 deletions being unique (File S1). The average size of these deletions was 53,001 ± 91,141, and they ranged from 1,058 nt to 690,380 nt (File S1). Reads with mapping ncRNAs were not evenly distributed in two populations. The CEU population had 65 deletions with mapping ncRNA reads, or 1.58 per individual on average, whereas the Yoruba population had 163 deletions, or 4.29 per individual on average (File S1). Out of these, 40 unique deletions with mapping reads were found in the CEU population, and 98 in the Yoruba population. No correlation between the size of the deletions and the number of occurrence of these deletions in the population was found for 1114 (r = 0.001) or 229 deletions (r = 0.025).

**Figure 1:**
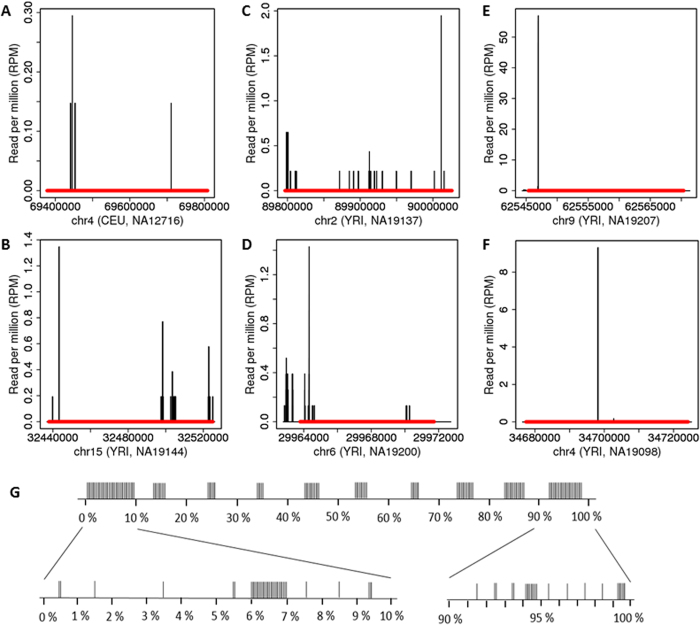
An example of ncRNA reads mapped to the regions between −1000 nt from the 5′-end and +1000 nt from the 3′-end of deletions. **(A–F)** The X-axis is the genomic coordinate of the indicated chromosome, and the Y-axis is RPM of mapped reads of ncRNAs. The red horizontal bar indicates a deletion. The name of CEU or YRI samples is in parentheses. The number of reads per deletion was calculated by dividing the total read count by the number of deletions in a 10 kb interval. **(G)** The figure depicts the distribution of ncRNA reads in deletion regions. Each deletion was divided into 10 equal bins, and reads in each bin were counted. The percentage indicates the relative distance of each bin from the start position of deletions. The one vertical bar corresponds to 10 ncRNA reads.

The reads were positioned in different regions of deletions and in close proximity to the deletion sites (Fig. 1A–F). The analysis of read distribution across all deletions showed that the reads were overrepresented at the deletion ends (Fig. 1G). The number of reads mapped to 1114 deletions did not correlate with the size of the deletions (r = 0.1, p = 0.0006; Fig. S1).

For 229 deletions, the reads were distributed equally between sense and antisense strands, approximately 55% and 45%, respectively (Table S1). Only two read pairs overlapped between sense and antisense strands (Tables S2 and S3). For 229 deletions with the mapped reads, the maximum number of reads, 218, mapping to any deletion was observed for the YRI sample NA19207 (Fig. 1E; Table S4). It was observed that 14 out of 46 unique reads mapping to this deletion region also multimapped to non-deletion regions, whereas the remaining 32 unique reads mapped exclusively to the deletion region. The analysis of the multimapped reads showed that the sequences of all 14 short reads were identical with the long sequence reads, and therefore are likely derivatives (degradation products) of the longer reads (Fig. S2). The 206 out of 218 reads in the deletion mapped to the high peak region around the coordinate 62546940, where no genes were located (Fig. S3), whereas the other 12 reads mapped outside the peak region. The second highest number of reads, 58 (i.e., 9.47 rpm), was found in the deletion region of the YRI sample NA19098 (see Fig. 1F).

Out of the remaining 227 deletions, 153 deletions contained a single ncRNA read at nucleotides, whereas in the remaining 74 deletions, the number of reads at nucleotides varied from 2 to 32 ncRNAs per deletion. The analysis of read distribution across all 229 deletions showed a typical Poisson distribution (Fig. S4).

### The enrichment of ncRNA reads at the deletions

**Figure 2:**
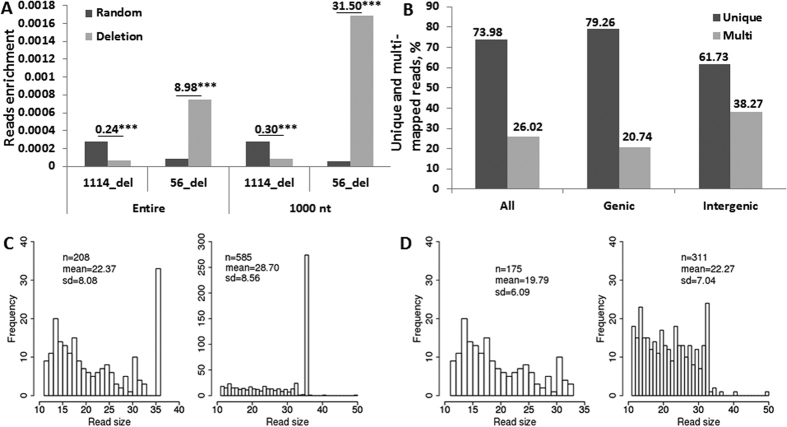
Characterization of reads located 1000 nt around the 5′ and 3′ ends. (**A**) Enrichment of reads in the deletion region versus the random region calculated for the entire region or for the first and last 1000 nt. Reads were counted either in the entire region or in the first and last 1000 nt regions of deletions and in random regions. Read counts were normalized to the size of regions, and then the normalized read counts were divided by the number of deletions. Calculations were done separately for all 1114 deletions or only for 56 deletions. The enrichment score of reads was calculated as the ratio of the mean read count in a deletion to the mean read count in the random region. The P-value was calculated by the two-sample Wilcoxon test of the mean difference. (**B**) Distribution of unique and multimapped reads. The values represent the percentage of unique and multimapped ncRNA reads stemming from deletions overlapping all regions, the genic and intergenic regions. (**C**) Distribution of sizes of unique reads in the −/+1000 nt regions of the 5′- and 3′-ends of deletions (left) and random regions (right). The n indicates the total number of reads, and the mean indicates the mean size of reads. The sd means the standard deviation. The P-value of the two-sample Wilcoxon test with regard to the mean difference of read sizes between the deletion and random region is less than 2.2e-16. (**D**) Distribution of sizesof unique reads excluding 36 nt reads in the −/+1000 nt regions of the 5′- and 3′-ends of deletions (left) and random regions (right). The same as in C.

The reads mapped to the entire regions of 1114 deletions and the corresponding 1114 random regions were counted. As seen in Fig. 2, read counts in deletions are significantly lower than those in random regions (enrichment of 0.24, p < 0.001). Similarly, there were fewer reads in the regions of the first and last 1000 nt of deletion as compared to corresponding random regions (enrichment of 0.30, p < 0.001). However, the same analysis on 56 deletions of which the first or last 1000 nt regions contained at least a single read showed that these deletions had significantly more reads than the corresponding 56 random regions (Fig. 2A).

30 out of 56 deletions, or 53.57%, overlapped genic regions (Table 2 and Table S5); this was much higher than the 34.11% of deletions that overlapped genic regions in all 1114 deletions (p = 0.001). Out of these 30 deletions, 17 overlapped exon regions and 13–intron regions. Again, the percentage of deletions overlapping the exon regions (56.67%) in these 56 deletions was much higher than that of 39.11% in 1114 deletions (p = 0.047; Table 2). The mapping analysis showed that reads mapping to the deletions which overlapped genic regions multimapped in 20.74% of cases, whereas reads from intergenic regions multimapped in 38.27% of cases (Fig. 2B).

### Characterizing reads at the 5′ and 3′ ends of deletions

Unique reads at the deletion ends are significantly shorter on average than those ones in random regions, 22.37 nt and 28.7 nt, respectively (p = 0.00) (Fig. 2C). The difference was still significant even when a fraction of 36 nt reads was eliminated from the analysis (p = 0.00; Fig. 2D). The ncRNA reads of 50 nt were largely absent, mostly due to a low contribution of 50 nt reads in the original sequencing data (Fig. S5).

**Figure 3:**
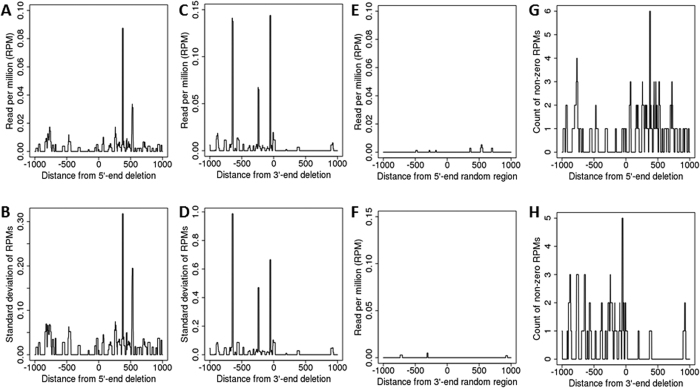
The average RPM of reads mapping to the ends of deletions and random regions. Reads were aligned at either the 5′-end (**A**) or the 3′-end (**C**) of 56 deletions and the corresponding 56 random regions (**E** for the 5′-end and **F** for the 3′-end), and RPMs of reads were averaged over positions (with standard deviations, **B**–for the 5′-end and **D**–the 3′-end). The value of “0” indicates the 5′- or 3′-end position of deletions or random regions. The region from “0” to “1000” indicates the deletion region at the 5′-end (**A**), and the region from “0” to “−1000” indicates the deletion region at the 3′-end (**C**). The non-zero RPM count of reads aligned at the 5′-end (**G**) and 3′-end (**H**) of the deletions.

First, we analysed the reads in the −/+1000 nt regions at the deletion ends by comparing the read distribution between 56 deletions and the corresponding random regions. As shown in Fig. 3, there was a low peak of around −800 nt upstream of the 5′-end of deletions and a relatively high peak of around 380 nt within the deletions. Concerning the reads aligned at the 3′-end of deletions, multiple high peaks were observed only in the deletion regions. The number of non-zero RPMs at a high peak of about the 380 nt distance from the 5′-end deletions and the −60 nt distance from the 3′-end deletions was 6 and 5, respectively (Fig. 3G,H). We noticed that there were more reads in the regions on either side of the deletion end. Enrichment analysis comparing reads that map deletion or random regions for 56 deletion/random region pairs indeed showed that read numbers within −1000 nt upstream of the 5′-end and +1000 nt downstream of the 3′-end of the deletions were on average enriched 10.3- and 6.0-fold, respectively, over random regions (Table S6). More extensive analysis using 300 random comparisons for deletions also showed differences in the enrichment of reads at the deletion ends for 56 deletions as compared to random regions (~4.5-fold; p = 0.08) and even for 229 deletions (~4.5-fold; p = 0.09) (Table S7). The differences in enrichment were more prominent in the 3′-ends: 3.74-fold (p = 0.11) for 5′-ends and 7.65-fold (p = 0.067) for 3′-end for 229 deletions; and 3.07-fold (p = 0.13) and 6.52-fold (p = 0.04) for 5′- and 3′-ends, respectively (Table S7).

### Characterizing reads at the nucleotide level

The analysis of the genomic locations of 1114 deletions showed that only 6 of them actually overlapped the regions of known 6629 ncRNAs in the −/+1000 nt regions of the 5′- and 3′-ends of deletions. Surprisingly, no ncRNA read was mapped to the overlapping regions of these 6 deletions, suggesting that ncRNAs mapped around the ends of 1114 deletions can be considered unclassified. Next, we attempted to identify the nature of ncRNAs mapping to deletions using the position-weight matrix analysis^14^. The analysis did not find any similarity between ncRNA reads mapping to the deletion regions and any known ncRNA sequences (Fig. S6 and Table S8).

**Figure 4:**
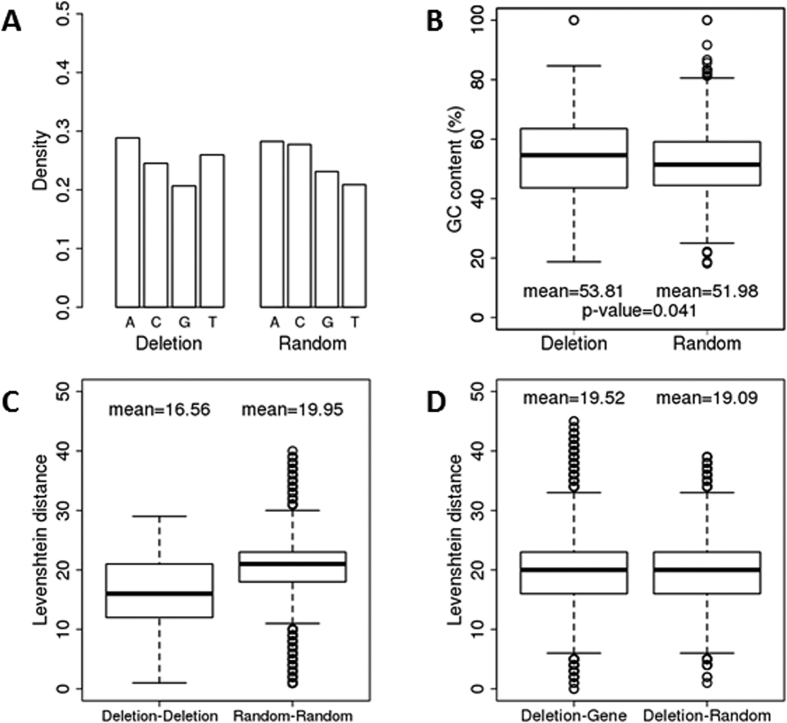
Characterization of ncRNA reads in the deletion regions and the corresponding random and gene regions. The nucleotide density at the first position (**A**) and the GC content (**B**) of unique read sequences in the deletions and random regions. The mean indicates the mean GC content of read sequences. The P-value was calculated by the two-sample Wilcoxon test with regard that the GC content around a deletion is higher than that around the random region. The Levenshtein distance between two unique read sequences. The distance was calculated between two sequences around the ends of deletions (Deletion-Deletion) and between two sequences around the ends of random regions (Random-Random) (**C**). The distance was calculated between the sequence around the ends of deletions and that one in gene regions (Deletion-Gene), and between the sequence around the ends of deletions and that one in random regions (Deletion-Random) (**D**). The mean indicates the mean of distances between two sequences. The P-value in **C** and **D** is less than 2.2e-16.

Next, we analysed the frequency of occurrence of a particular nucleotide at the first nucleotide position and the relative GC contents of unique read sequences around the ends of deletions and random regions (Fig. 4A,B). Compared to random regions, the frequency of T nucleotide at the first position of reads was relatively high around the deletion ends. Additionally, the GC content of sequences around the deletions was significantly higher than that around random regions.

The Levenshtein distance was subsequently calculated as the dissimilarity measure between two unique read sequences around the ends of deletions and random regions. The mean distance between two sequences around deletions was on average 16.56 nt or 74% (16.56/22.37) of the mean size of sequences (see Fig. 2C,D and Fig. 4C). In the meantime, the distance between two sequences around random regions was on average 19.95 nt, which equals 70% (19.95/28.70) of the mean size of sequences. Therefore, the sequences around deletions were not more similar to each other than the sequences around random regions. The Levenshtein distance calculated between unique read sequences around the ends of deletions and unique read sequences that map gene regions as well as between unique read sequences around the ends of deletions and unique read sequences that map random regions showed that the sequences around deletions were dissimilar to the sequences in gene regions in the equal degree of their dissimilarity to the sequences around random regions (Fig. 4D). Therefore, there was no sequence similarity to each other around deletions and to sequences in gene regions.

### The effect of sequence mismatches on the enrichment of ncRNA reads

**Figure 5:**
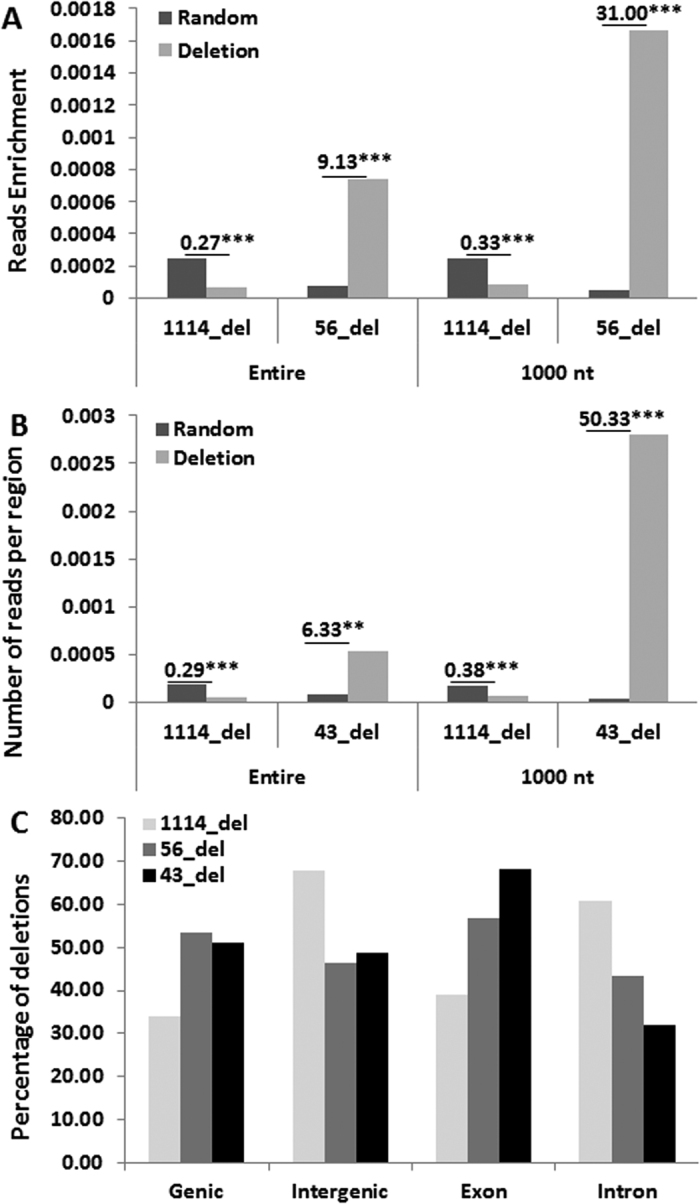
Characterization of reads located 1000 nt around the 5′ and 3′ ends using ncRNAs with 1 or 0 mismatches. Enrichment of reads in the deletion region versus the random region calculated for the entire region or for the first and last 1000 nt using ncRNAs with 1 mismatch (**A**) or 0 mismatches (**B**). Reads were counted either in the entire region or in the first and last 1000 nt regions of deletions and random regions, read counts were normalized by the region size, and then the normalized read counts were divided by the number of deletions. Calculations were done separately for all 1114 deletions or only for 56 deletions. The enrichment score of reads was calculated as the ratio of the mean read count in a deletion to the mean read count in the random region. The P-value was calculated by the two-sample Wilcoxon test of the mean difference. (**C**) The percentage of 1114, 56 and 43 deletions overlapping genic, intergenic, exon and intron regions. The percentage is calculated by dividing the number of deletions overlapping a specific region by the total number of deletions in one of three categories. “1114 deletions”–shows the data for all 1114 deletions found in the analyzed samples; “56 deletions”–shows the data for samples in which at least a single ncRNA read was matched to the first or last 1000 nt of the deletion, with 2 mismatches being used; “43 deletions”–the same as for “56 deletions” but when no mismatches were used. See Table S1 for details.

We showed that ncRNA sequences that multimap to other genomic regions were in most cases derivatives of larger ncRNAs from the deleted regions that were unique to these regions. Since in this analysis we allowed two mismatches between ncRNAs and the genomic sequence, we investigated the effect of sequence mismatches on the enrichment of ncRNA reads. Reducing the number of mismatches to 1 did not change the number of deletions that contained at least a single read in the first or last 1000 nt and nearly failed to change read enrichment (compare Fig. 5A to Fig. 2A). Reducing the mismatch number to 0 decreased the number of deletions to which the reads were mapped down to 43; reads mapping to these deletions were enriched even more than those with 1 or 2 mismatches (Fig. 5B). Enrichment analysis using 300 random comparisons with deletions when 1 or 0 mismatches were considered showed differences for 229 deletions–4.33-fold (p = 0.097) and 3.77-fold (p = 0.087) for 1 and 0 mismatches, respectively (Table S7). Similarly, for the deletions with reads at the deletion ends, the enrichments in 300 random comparisons when 1 and 0 mismatches were considered were also substantial, 4.22-fold (p = 0.08) and 10.63-fold (p = 0.04). Therefore, the most significant differences were observed when perfect homology between ncRNA reads and genomic regions was used (0 mismatches). Again, more prominent differences were observed at 3′-ends (Table S7).

In addition, these 43 deletions overlapped exon regions more frequently than 56 deletions or 1114 deletions (Table 2). We also found that both 56 and 43 deletions more often overlapped genic regions than intergenic regions, in contrast to 1114 deletions (Fig. 5C). When only the genic region is taken into consideration, the majority of 56 and 43 deletions overlapped exonic regions, with 43 deletions having a greater value.

### The origin of ncRNAs from the deletion regions

We hypothesized that ncRNA reads in the deleted regions that overlap genes could be the degradation products of mRNAs. The analysis was conducted separately for deletions that partially overlapped at least a single gene (51 samples) and deletions in which gene(s) were entirely within deletions (24 samples) (Tables S9 and S10). The first category had 17 samples with ncRNA reads, out of which 14 samples had no mRNA reads, whereas the other 3 samples had them. One out of the three remaining samples had 48-fold higher levels of mRNA reads stemming from the gene in the deleted region compared to the same genes in samples without deletions. The second category had 7 samples with ncRNA reads, and 5 of them had no mRNA reads.

## Discussion

The main finding of our research is the presence of ncRNA reads that match the deleted regions in human genomes. They typically do not match other genomic regions and are enriched at the deletion ends.

Previous work demonstrated that insertions and deletions (INDELs) are located in genic regions (~35.7%)^15^. Among them, ~1.2% (1713 out of 148 335) were located in exon regions and ~95.7% - in introns (with the remaining ones - in promoter and terminator regions)^15^. Our analysis returned the similar number of the percentage of deletions overlapping genes–34.1% when all 1114 deletions were analysed, although a larger proportion of them overlapped exons (12.6%) (Table 2). However, when only 56 deletions with ncRNA reads at the deletion ends are considered, deletions overlapped genes in 53.6% of cases, and over 30% of them overlapped exons (Table 2). Considering that exons occupy ~1.5% of the human genome^16^, deletions with ncRNA reads overlapping exons (over 30%) are substantially enriched in the analysed samples.

Although the deletion size did not correlate with the read number, we found that the deletion ends contained a larger number of reads. There were several important findings concerning these reads. First, there was more than a 30-fold enrichment of these reads over the corresponding random regions. The level of this enrichment was 50-fold when reads with 0 mismatches at nucleotides were considered (Fig. 5). Second, reads that were mapped to deletions overlapping genic regions multimapped in 20.74% of cases, whereas reads present in deletions that overlapped intergenic regions multimapped in 38.27% of cases. Since multimapped reads were short and were likely degradation products of longer reads, it can be suggested that ncRNAs matching intergenic regions degrade more frequently than those matching genic regions. Third, reads that mapped to the first or last 1000 nt of deletions were more frequently found in deletions that overlap genic and exon regions as compared to reads found in all other regions of deletions (Table 2). Moreover, ncRNAs without mismatches (43 deletions) match to exon regions more often than ncRNAs with mismatches (56 deletions). Fourth, the nucleotide distribution around the ends of deletions is not similar to any nucleotide distribution of known ncRNA sequences. This suggests that ncRNAs mapping to deletion regions cannot be classified as any known ncRNAs, and they likely represent degradation products of longer RNAs; these longer RNAs are unlikely mRNA reads because deletions that contained ncRNAs overlapping genes in most cases did not contain mRNA reads. Finally, the GC content of sequences of ncRNAs mapping to deletions was 53.81%, whereas in random regions, it was 51.98%. The average GC content of the human genome is ~41%, and it is higher in genic regions and in exons rather than introns^17^. Therefore, a higher GC content of ncRNA reads correlates with the fact that they match genic (and exonic) regions more frequently.

Through the bioinformatics analysis of genomic sequences and matching ncRNA reads, we have identified ncRNA reads mapping to the deleted genomic regions. The simplest explanation of our finding is that all deletions to which the reads map were not homozygous, despite the fact that these genomic sequences were not present in the corresponding genomes^18^. However, this does not explain the enrichment of ncRNAs at the deletion ends. The report that utilized the same data set attempted to prove that identified deletions were homozygous^18^. Ten genes that were deleted at a relatively high level were confirmed as homozygous nulls by quantitative PCR^18^. In addition, the authors analysed another 60 deletions that occurred less often and also showed that most of them were homozygous. In our work, we showed that out of 24 samples that contained ncRNA reads, 5 also contained mRNA reads, suggesting that at least some of these deletions were likely heterozygous.

Our main hypothesis is that ncRNAs mapping to the deleted regions have been transcribed from these genomic regions in previous generations and can represent the degradation products from RNAs from these regions. Since we do not have any information about the genome of parents of individuals with deletions, we do not know when and how these deletions have occurred. The appearance and propagation of homozygous deletions may only occur frequently in isolated populations. Therefore, some of these deletions can be common deletion variants found in humans^18^. It is possible that an ncRNA cache is propagated across a single generation or multiple generations through gametes. More than 50% of mammalian RNAs overlap a transcript from the opposite strand in a divergent, convergent, or full-length configuration^19^. Thus, dsRNAs are likely formed and can serve as templates for self-propagation. It should be noted, however, that we observed a little overlap between ncRNA reads matching sense and antisense strands.

Why then are these ncRNAs enriched at the deletion ends? It can be hypothesized that when deletions form, RNA-mediated repair is attempted, but it fails to repair strand breaks. Two previous reports showed the enrichment of double-strand break interacting RNAs (diRNAs) around the sites of double-strand breaks. It is highly likely that ncRNAs which we identified are some type of diRNAs^20^,^21^. It is also possible that the deletions are the result of RNA-mediated DNA rearrangement, although the process has never been reported in humans.

How else can we describe the presence of ncRNA reads that map to the non-existing genomic positions? Although there is a remote possibility that the deleted region was translocated, it is highly unlikely because we checked the genomic sequences of individual samples for the presence of deleted sequences. Theoretically, it is possible that RNA editing could substantially modify ncRNAs such that they no longer map to the original source. However, the analysis of ncRNAs from random regions showed that they only match to the respective genomic regions (data not shown). This suggests that a high rate of editing among the analysed ncRNA sequences is unlikely to occur. Finally, the presence of these ncRNAs could also be explained by contamination from other samples or sources, though it still does not explain enrichments or bias we found while trying to characterize the reads.

Further work will claify whether these ncRNAs play any role in the maintenance of genome stability and the mechanisms of inheritance.

## Methods

### Data sources

The small ncRNA sequencing data of the 41 CEPH (CEU) and 38 Yoruba (YRI) samples of the 1000 genomes shared by HapMap data^18^ were downloaded from http://www.ebi.ac.uk/arrayexpress/experiments/E-GEUV-2/.22 The processed mRNA sequencing data were downloaded from http://www.ebi.ac.uk/arrayexpress/experiments/E-GEUV-1/files/processed/.22 The sequencing platforms were Illumina HiSeq 2000 with single-end 36-bp small RNA-seq and paired-end 75-bp mRNA-seq^22^. The analysis of the data published by McCarroll *et al.*^18^ revealed the presence of 641 individual deletions in 41 CEU samples and 473 individual deletions in 38 YRI samples^18^. Since the coordinates of deletions were in the human genome hg16, the hg16 genome sequence was downloaded from http://hgdownload.cse.ucsc.edu/goldenPath/hg16/bigZips/. The ncRNA and gene information of the hg16 genome was downloaded from http://hgdownload.cse.ucsc.edu/goldenPath/hg16/database/. In addition, ncRNA sequences were downloaded from ftp://ftp.ensembl.org/pub/release-25/human-25.34e/data/fasta/rna/.

### Mapping sequencing data to the reference genome

The adapter sequences were removed from small ncRNA sequencing data, and the trimmed sequences shorter than 12 nt were discarded using Cutadapt^23^. The sequences were subsequently mapped to the hg16 genome using Bowtie 2 with the SAM output options of “–no-unal –no-hd –no-sq -S”^24^, and sequences with more than two mismatches were excluded. Since mappers such as Bowtie and TopHat use the default value of 2 for the maximum number of mismatches^25^,^26^, sequence mismatches were allowed up to 2 mismatches for the analysis. The read per million (RPM) was calculated as ([*# of mapped reads*] × *10*^*6*^*/*[*total reads of sample*]). In addition, we have also performed the same analysis in which either a single mismatch or zero mismatches were allowed.

### Deletions and the corresponding random regions

For each of 1114 deletions, the corresponding random region with the same size as the deletion size was randomly (computer generated) chosen from the same chromosome of the same sample of the deletion so that the region between −1000 nt from its 5′-end and +1000 nt from its 3′-end did not overlap with any deletion (see Fig. S7).

To analyse whether the size of deletions is correlated with the number of ncRNAs mapped to them, the range of deletion sizes was divided into bins of a 10-kb interval, the number of ncRNA reads was counted in each bin, and then the number of reads per deletion was calculated by dividing the total read count by the number of deletions in each bin. The correlation coefficient between the size of deletions and the number of reads is 0.1 (p = 0.0006).

The analysis of the nucleotide density at the first nucleotide and the GC content of reads was performed in the reads mapping to the 1000 nt regions at the 5′- (0 to +1000) and the 3′- (0 to −1000) ends of the deletion and the corresponding random regions.

### The read enrichment in the deletion and random regions

Within the deletion and random regions, reads were counted either in the entire regions, the 1000 nt upstream regions, the 1000 nt downstream regions, or in the first and last 1000 nt of the deletion regions or in the random regions. Read counts were normalized by the sizes of regions, and then the normalized read counts were averaged by the number of deletions. An enrichment score of reads was calculated as the ratio of the mean read count in the deletion to the mean read count in the random region. Also, the p-value was calculated by the two-sample Wilcoxon test of the mean difference between the normalized read counts in the deletion and random regions.

The random regions of 300 random sets corresponding to the deletions were generated in the manner described above. Then, the reads in the 5′- and 3′-end and non-end regions of the deletions and random regions were counted. The non-end region is the remaining region other than the 5′- and 3′-end regions within the deletion or the random region. The end regions were defined as the first and last 100 nt regions for the deletion or the random region in the sizes less than 1000 nt, 200 nt for 2000 nt, 500 nt for 3000 nt, and 1000 nt for the deletion or the random region in the size greater than or equal to 3000 nt. For each of the deletion set and the 300 random sets, the total read counts in the 5′-end region, the 3′-end region, and the non-end region were normalized by the total size of the regions. The enrichment score of reads in the end region was calculated as the ratio of the normalized total read count in the end region to the normalized total read count in the non-end region. Then the enrichment score of deletions was compared with the median value of the 300 random enrichment scores. For the significance of the read enrichment in the end region of deletions, a randomization test was performed^27^. The p-value was calculated as the proportion of the random sets with an enrichment score greater than the enrichment score of deletions in the 300 random sets.

For the analysis of significance of shorter read sizes around the end regions of deletions as compared to random regions, the randomization test to compare the average read sizes in the −/+1000 nt regions of the 5′- and 3′-ends of deletions and random regions was performed using the 300 random sets^27^. The p-value was calculated as the proportion of the random sets with the average read size smaller than the average read size of deletions in the 300 random sets.

### Read counts mapped to forward and reverse strands

Reads mapped to forward and reverse strands in the deletion regions were extracted and counted. Reads overlapping between forward and reverse strands were examined.

### Deletions in the genic, intergenic, exon and intron regions

If a deletion overlapped any gene region, it was regarded as being in the genic region. Otherwise, it was considered as being in the intergenic region. Among the deletions in the genic regions, if a deletion overlapped any exon region, it was regarded as being in the exon region. Otherwise, it was considered as being in the intron region. Fisher’s exact test was performed to test the alternative hypothesis that the proportion of deletions overlapping gene regions or exon regions is lower among the 1114 deletions than among the 56 deletions.

### Reads mapped between 62546298 and 62547331 on chromosome 9

The file of samples containing the deletion was loaded, and the mapped reads were viewed in the range of 62546298 and 62547331 on chromosome 9 by Integrated Genome Browser (IGB)^28^.

### The dissimilarity between two read sequences

The Levenshtein distance was calculated as the dissimilarity measure of two read sequences using the R package stringdist^29^. It counts the number of nucleotides that need to be deleted, inserted, and replaced to make two sequences identical^29^. If two sequences are exactly the same, the distance is 0. If two sequences are completely different, the distance is the length of a longer sequence. To calculate the Levenshtein distance, a comparison was done for reads mapping within the region from 0 to +1000 nt from the 5′ end and from 0 to −1000 nt from the 3′ end in the deletion and random regions, whereas in the gene region, reads mapped to the entire gene (randomly chosen on the same chromosome on which a deletion was found) were used.

### Position weight matrix analysis

The sequences of all known ncRNAs and the sequences of unique reads around the ends of deletions were extracted, and sequence logos of nucleotides at the first 15 nt positions of their 5′-ends were drawn by WebLogo at http://weblogo.berkeley.edu/logo.cgi. Sequence logos are composed of stacks of nucleotide symbols^30^. The height of each symbol represents the relative frequency of a nucleotide at the position, and the overall height of each stack represents the sequence conservation at the position^30^. As seen on Fig. S6 and Table S7, sequences around the ends of deletions do not show a pattern similar to known ncRNA sequences.

## Additional Information

**How to cite this article**: Byeon, B. and Kovalchuk, I. Non-coding RNAs match the deleted genomic regions in humans. *Sci. Rep.* **6**, 37452; doi: 10.1038/srep37452 (2016).

**Publisher’s note:** Springer Nature remains neutral with regard to jurisdictional claims in published maps and institutional affiliations.

## Supplementary Material

Supplementary Information

Supplementary File

## Figures and Tables

**Table 1:** Characterization of deletions in CEU and Yoruba populations.

Deletion	Start	End	Size	CEU	Yoruba
Unique					
Ch1	149,786,102	149,800,260	14,158	10	
Ch3	194,200,830	194,204,450	3,620	19	
Ch3	163,860,609	163,861,908	1,299	19	
Ch3	163,833,596	163,892,785	59,189	1	
Ch3	163,833,596	163,943,569	109,973		12
Ch4	92,391,958	92,393,093	1,135	10	
Ch6	103,787,052	103,794,525	7,473	12	
Ch7	89,422,556	89,424,158	1,602	13	
Ch7	141,462,154	141,472,512	10,358	10	
Ch7	141,462,154	141,472,285	10,131		19
Ch7	141,456,537	141,472,512	15,975	12	
Ch7	141,456,537	141,472,285	15,748		19
Ch7	133,203,070	133,212,391	9,321	10	
Ch8	16,212,027	16,216,726	4,699		10
Ch8	39,271,742	39,390,071	118,329	12	
Common					
Ch1	55,871	68,941	13,070	2	2
Ch3	99,731,950	99,732,552	602	8	1
Ch4	9,969,524	9,980,122	10,598	22	8
Ch4	23,759,754	23,765,449	5,695	1	1
Ch4	119,071,973	119,076,909	4,936	1	1
Ch4	138,551,715	138,556,270	4,555	1	2
Ch7	109,003,350	109,007,346	3,996	8	3
Ch7	125,601,054	125,603,762	2,708	4	14
Ch8	7,201,387	7,206,953	5,566	1	1
Ch12	88,992,727	88,993,412	685	2	1

For the analysis of unique deletions overrepresented in the population, deletions that occur in ≥25% (10 individuals or more) were used. “Unique” indicate deletions occurring in either CEU or Yoruba and “Common” indicate deletions occurring in both populations.

**Table 2:** The number and the percentage of deletions overlapping various genomic regions.

The total number of deletions	Deletions overlapping gene regions	Deletions overlapping intergenic regions	Deletions overlapping exon regions	Deletions overlapping intron regions
1114 deletions	358	756	140	218
	34.1%	67.9%	39.1% (12.6%)	60.9% (19.6%)
229 deletions	106	123	68	38
	46.3%	53.7%	64.2% (29.7%)	35.8% (16.6%)
56 deletions	30	26	17	13
	53.6%	46.4%	56.7% (30.4%)	43.3% (23.2%)
43 deletions	22	21	15	7
	51.2%	48.8%	68.2% (34.9%)	31.8% (16.3%)

The values are the number of deletions that overlap gene regions and intergenic regions, and the number of deletions in exon and intron regions. The percentage is calculated by dividing the number of deletions in a specific region by the total number of deletions. “1114 deletions”–shows data for all 1114 deletions found in the analyzed samples; “229 deletions”–shows the data for samples in which at least a single ncRNA read in a deletion was found; “56 deletions”–shows the data for samples in which at least a single ncRNA read was matched to the first or last 1000 nt of the deletion, with 2 mismatches being used; “43 deletions”–the same as for “56 deletions” but when no mismatches were used. For deletions overlapping exonic or intronic regions, the percentage shows the fraction from deletions overlapping genic regions. The numbers in parenthesis show the percentage of the entire genomic region.
