# Supplementary material for: Non-coding RNAs match the deleted genomic regions in humans
Source: Sci Rep. 2016 Nov 17;6:37452. doi: 10.1038/srep37452 (PMC5112596; doi:10.1038/srep37452)
Supplement: Supplementary Information [file srep37452-s1.doc]

**Non-coding RNAs match the deleted genomic regions in humans**

Boseon Byeon and Igor Kovalchuk*


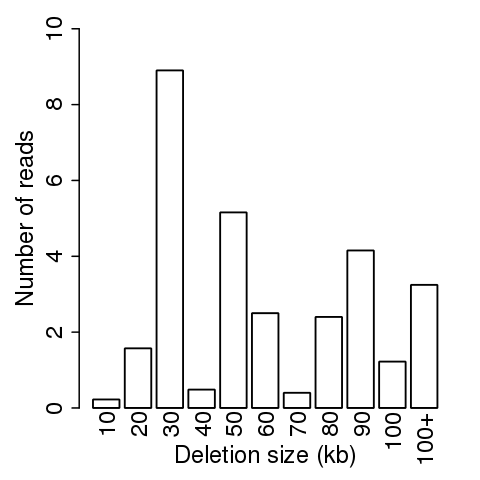


**Figure S1. The average number of reads per deletion of a certain size**

The number of reads per deletion was calculated by dividing the total read count by the number of deletions in a 10 kb interval. 100+ indicates a deletion size larger than 100 kb. The number of reads mapped to 1114 deletions did not correlate with the size of deletions (r=0.1, p-value=0.0006; Fig. S1).


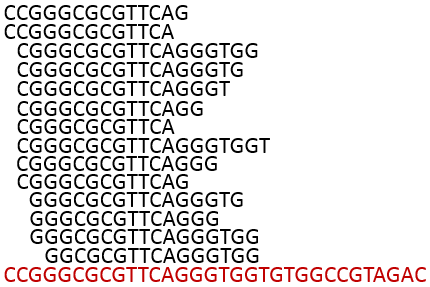


**Figure S2. Multi-mapped and uniquely mapped read sequences**. A sequence in red is the sequence of a uniquely mapped read, and sequences in black are sequences of 14 multi-mapped reads.


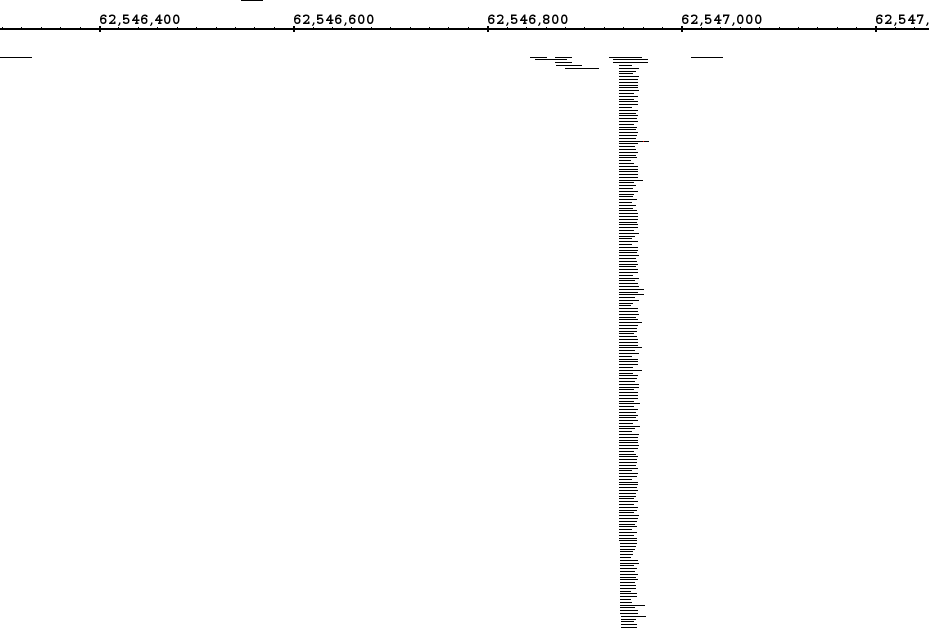

**Figure S3. Mapping of reads in the 1 kb region between coordinates 62546298 and 62547331 on chromosome 9 of YRI sample NA19207**

**Figure S4. The read distribution across all 229 deletions (A) or 227 deletions that exclude two largest deletions - 218 and 58 nucleotides (B).** The Y-axis shows the deletion number and the X-axis shows the read number.

**Figure S5. Distribution of 36 and 50 nt reads in the sequencing data.**

An upper panel shows the data for CEU, and a lower panel for Yoruba groups. The Y-axis shows the read number, and the X-axis shows the read size.


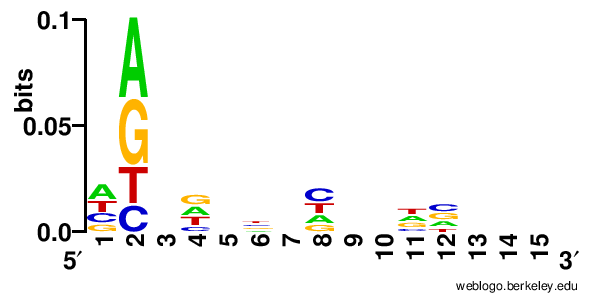

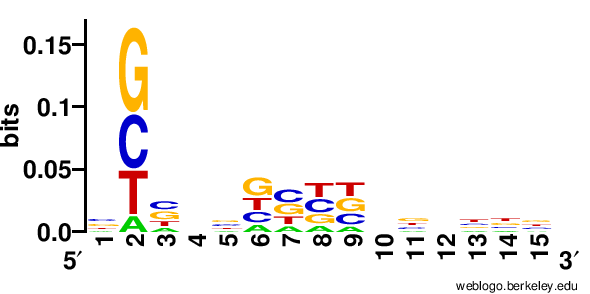


**Figure S6. Sequence logo of the first 15 nucleotides at the 5’-end of unique read sequences in the -/+1000 nt regions of the 5’- and 3’-ends of deletions (left) and miRNA sequences (right)**. Read sequences of a size less than 18 nt around the ends of deletions were excluded. Sequence logos were drawn by WebLogo at http://weblogo.berkeley.edu/logo.cgi. Although miRNA sequences and sequences around the ends of deletions are more conservative at the second nucleotide position than at other positions, sequences around the ends of deletions have a much higher relative frequency of A nucleotide than miRNA sequences at the position.


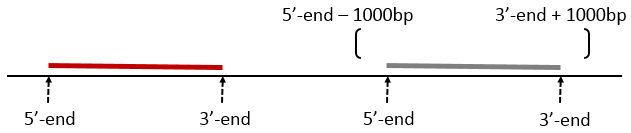


**Figure S7. Deletion and random regions.** The size of the random region is the same as the size of the corresponding deletion region. The red horizontal bar indicates a deletion, and the grey one is the corresponding random region.

**Table S1. List of deletions with reads matching to sense and antisense strands**

“Deletion” - deletion catalogue number; “Sample” - sample number; “Chr” - chromosome number; “Start” - start of the deletion; “End” - end of the deletion; “Read” - number of reads mapping to the deletion; “Forward” - number of reads mapping to the sense strand; “Reverse” - number of reads mapping to the antisense strand.

| **Deletion** | **Sample** | **Chr** | **Start** | **End** | **Read** | **Forward** | **Reverse** |
| --- | --- | --- | --- | --- | --- | --- | --- |
| yri1179 | NA19207 | chr9 | 62545375 | 62570383 | 218 | 3 | 215 |
| yri542 | NA19098 | chr4 | 34677422 | 34724191 | 58 | 1 | 57 |
| yri863 | NA19143 | chr12 | 63304111 | 63323750 | 35 | 34 | 1 |
| yri875 | NA19144 | chr15 | 32437866 | 32525037 | 34 | 13 | 21 |
| yri778 | NA19137 | chr2 | 89796705 | 90026105 | 34 | 18 | 16 |
| yri702 | NA19129 | chr1 | 149785060 | 149797102 | 32 | 32 | 0 |
| yri701 | NA19129 | chr1 | 149771758 | 149798424 | 32 | 32 | 0 |
| yri703 | NA19129 | chr1 | 149786102 | 149798424 | 32 | 32 | 0 |
| yri729 | NA19130 | chr6 | 103787052 | 103807031 | 26 | 22 | 4 |
| yri1204 | NA19209 | chr3 | 163833596 | 163943569 | 26 | 17 | 9 |
| yri126 | NA18505 | chr2 | 89796705 | 90026105 | 24 | 20 | 4 |
| ceu799 | NA12005 | chr12 | 96517213 | 96533447 | 21 | 0 | 21 |
| yri128 | NA18505 | chr2 | 89826086 | 89981417 | 19 | 16 | 3 |
| yri1063 | NA19200 | chr6 | 29963788 | 29971727 | 19 | 19 | 0 |
| ceu1215 | NA12760 | chr2 | 89093935 | 89175498 | 17 | 8 | 9 |
| ceu635 | NA11829 | chr7 | 141921685 | 141931471 | 15 | 0 | 15 |
| yri1177 | NA19207 | chr9 | 36872141 | 36890817 | 15 | 1 | 14 |
| yri181 | NA18508 | chr1 | 146563653 | 146572086 | 15 | 15 | 0 |
| yri548 | NA19099 | chr1 | 146591613 | 146605848 | 15 | 2 | 13 |
| yri742 | NA19131 | chr4 | 34685154 | 34701647 | 14 | 0 | 14 |
| yri743 | NA19131 | chr4 | 34686467 | 34707485 | 14 | 0 | 14 |
| yri217 | NA18517 | chr1 | 149771758 | 149798424 | 14 | 14 | 0 |
| yri329 | NA18858 | chr4 | 34685154 | 34701647 | 14 | 0 | 14 |
| yri1176 | NA19207 | chr8 | 39271742 | 39390862 | 13 | 6 | 7 |
| yri127 | NA18505 | chr2 | 89803197 | 89848524 | 13 | 11 | 2 |
| yri218 | NA18517 | chr1 | 149785060 | 149797102 | 13 | 13 | 0 |
| yri219 | NA18517 | chr1 | 149786102 | 149798424 | 13 | 13 | 0 |
| ceu273 | NA07357 | chr9 | 32991449 | 33014917 | 12 | 2 | 10 |
| yri1167 | NA19207 | chr1 | 149771758 | 149798424 | 11 | 11 | 0 |
| yri1168 | NA19207 | chr1 | 149786102 | 149798424 | 11 | 11 | 0 |
| yri859 | NA19143 | chr1 | 149771758 | 149798424 | 11 | 11 | 0 |
| yri860 | NA19143 | chr1 | 149786102 | 149798424 | 11 | 11 | 0 |
| yri876 | NA19144 | chr22 | 21359787 | 21388825 | 11 | 10 | 1 |
| yri120 | NA18505 | chr1 | 149785060 | 149797102 | 9 | 9 | 0 |
| yri119 | NA18505 | chr1 | 149771758 | 149798424 | 9 | 9 | 0 |
| yri121 | NA18505 | chr1 | 149786102 | 149798424 | 9 | 9 | 0 |
| yri642 | NA19119 | chr1 | 149771758 | 149798424 | 9 | 7 | 2 |
| yri1060 | NA19200 | chr4 | 34677422 | 34724191 | 8 | 0 | 8 |
| yri511 | NA19093 | chr6 | 29963788 | 29971727 | 8 | 8 | 0 |
| yri916 | NA19153 | chr4 | 70471691 | 70542965 | 8 | 1 | 7 |
| yri68 | NA18502 | chrX | 65105136 | 65531010 | 8 | 2 | 6 |
| yri1119 | NA19204 | chr12 | 130253222 | 130299606 | 8 | 4 | 4 |
| yri1254 | NA19222 | chr7 | 104193511 | 104201772 | 8 | 0 | 8 |
| ceu258 | NA07357 | chr22 | 21118175 | 21360293 | 7 | 3 | 4 |
| yri1059 | NA19200 | chr3 | 163833596 | 163943569 | 7 | 3 | 4 |
| ceu859 | NA12044 | chr4 | 10148210 | 10151039 | 6 | 6 | 0 |
| yri1211 | NA19210 | chr13 | 18261867 | 18268071 | 6 | 0 | 6 |
| yri955 | NA19160 | chr4 | 69432417 | 69486334 | 6 | 1 | 5 |
| ceu997 | NA12234 | chr8 | 14647130 | 15337510 | 5 | 3 | 2 |
| ceu998 | NA12234 | chr8 | 14650691 | 15336034 | 5 | 3 | 2 |
| ceu1119 | NA12716 | chr4 | 69378123 | 69808237 | 5 | 1 | 4 |
| yri1053 | NA19200 | chr1 | 149771758 | 149798424 | 5 | 5 | 0 |
| yri649 | NA19119 | chr4 | 70471691 | 70542965 | 5 | 0 | 5 |
| yri643 | NA19119 | chr12 | 11400655 | 11434605 | 5 | 3 | 2 |
| yri1210 | NA19210 | chr12 | 81459099 | 81470766 | 5 | 0 | 5 |
| yri831 | NA19141 | chr12 | 22086469 | 22099211 | 5 | 5 | 0 |
| ceu259 | NA07357 | chr22 | 21396778 | 21538141 | 4 | 4 | 0 |
| ceu5 | NA06985 | chr12 | 131823562 | 131838245 | 4 | 4 | 0 |
| ceu62 | NA06994 | chr22 | 21104182 | 21129536 | 4 | 4 | 0 |
| ceu1438 | NA12872 | chr9 | 81945705 | 81999325 | 4 | 3 | 1 |
| yri724 | NA19130 | chr2 | 41213645 | 41220036 | 4 | 0 | 4 |
| yri736 | NA19130 | chr8 | 115126252 | 115130784 | 4 | 2 | 2 |
| yri1054 | NA19200 | chr1 | 149786102 | 149798424 | 4 | 4 | 0 |
| yri1056 | NA19200 | chr10 | 46327384 | 46342622 | 4 | 4 | 0 |
| yri738 | NA19131 | chr10 | 54723271 | 54798755 | 4 | 3 | 1 |
| yri746 | NA19131 | chr6 | 103787052 | 103807031 | 4 | 1 | 3 |
| yri914 | NA19153 | chr14 | 74338282 | 74350474 | 4 | 1 | 3 |
| yri1121 | NA19204 | chr3 | 163833596 | 163943569 | 4 | 3 | 1 |
| yri1072 | NA19201 | chr4 | 70471691 | 70542965 | 4 | 1 | 3 |
| yri600 | NA19102 | chr20 | 16562202 | 16580314 | 4 | 2 | 2 |
| yri787 | NA19138 | chr2 | 71306841 | 71317129 | 4 | 1 | 3 |
| yri788 | NA19138 | chr3 | 163833596 | 163943569 | 4 | 2 | 2 |
| yri452 | NA18912 | chr8 | 4619513 | 4691949 | 4 | 4 | 0 |
| yri908 | NA19152 | chr3 | 46758432 | 46807284 | 4 | 2 | 2 |
| yri1008 | NA19172 | chr4 | 69432417 | 69486334 | 4 | 0 | 4 |
| yri1263 | NA19223 | chrX | 107662335 | 107675738 | 4 | 0 | 4 |
| ceu999 | NA12234 | chr8 | 14978429 | 15392548 | 3 | 2 | 1 |
| ceu660 | NA11831 | chr8 | 39271742 | 39390071 | 3 | 1 | 2 |
| ceu1326 | NA12812 | chr3 | 163840486 | 163939798 | 3 | 1 | 2 |
| yri1173 | NA19207 | chr4 | 70477074 | 70542965 | 3 | 1 | 2 |
| yri727 | NA19130 | chr3 | 127035541 | 127042413 | 3 | 1 | 2 |
| yri123 | NA18505 | chr12 | 50791226 | 50819085 | 3 | 3 | 0 |
| yri125 | NA18505 | chr2 | 71306841 | 71317129 | 3 | 0 | 3 |
| yri58 | NA18502 | chr14 | 74338282 | 74350474 | 3 | 3 | 0 |
| yri949 | NA19160 | chr14 | 74338282 | 74350474 | 3 | 3 | 0 |
| yri784 | NA19138 | chr1 | 16392736 | 16400201 | 3 | 0 | 3 |
| yri909 | NA19152 | chr3 | 163833596 | 163943569 | 3 | 1 | 2 |
| yri1158 | NA19206 | chr2 | 203499611 | 203511609 | 3 | 3 | 0 |
| yri941 | NA19159 | chr9 | 11903287 | 11978036 | 3 | 2 | 1 |
| yri1202 | NA19209 | chr12 | 94301826 | 94311594 | 3 | 2 | 1 |
| ceu824 | NA12006 | chr22 | 37615466 | 37624865 | 2 | 2 | 0 |
| ceu832 | NA12006 | chr8 | 39271742 | 39390071 | 2 | 1 | 1 |
| ceu939 | NA12154 | chr1 | 172037243 | 172041015 | 2 | 2 | 0 |
| ceu82 | NA07000 | chr2 | 89039268 | 89049267 | 2 | 0 | 2 |
| ceu445 | NA10851 | chr3 | 163840486 | 163939798 | 2 | 2 | 0 |
| ceu464 | NA10851 | chr8 | 39271742 | 39390071 | 2 | 1 | 1 |
| ceu1450 | NA12873 | chr4 | 69441695 | 69482361 | 2 | 0 | 2 |
| ceu1451 | NA12873 | chr8 | 39250107 | 39397764 | 2 | 2 | 0 |
| ceu1452 | NA12873 | chr8 | 39268398 | 39389812 | 2 | 2 | 0 |
| yri539 | NA19098 | chr21 | 34400727 | 34401819 | 2 | 2 | 0 |
| yri534 | NA19098 | chr18 | 64171649 | 64270532 | 2 | 2 | 0 |
| yri870 | NA19143 | chr4 | 70477074 | 70542965 | 2 | 0 | 2 |
| yri777 | NA19137 | chr12 | 32414722 | 32422479 | 2 | 1 | 1 |
| yri725 | NA19130 | chr20 | 1564704 | 1567374 | 2 | 0 | 2 |
| yri728 | NA19130 | chr4 | 108651560 | 108665451 | 2 | 1 | 1 |
| yri133 | NA18505 | chr3 | 46758432 | 46807284 | 2 | 1 | 1 |
| yri134 | NA18505 | chr3 | 163833596 | 163943569 | 2 | 1 | 1 |
| yri1061 | NA19200 | chr4 | 69450972 | 69458490 | 2 | 1 | 1 |
| yri1052 | NA19200 | chr1 | 72137668 | 72147489 | 2 | 2 | 0 |
| yri1064 | NA19200 | chr7 | 141456537 | 141472285 | 2 | 0 | 2 |
| yri1065 | NA19200 | chr7 | 141462154 | 141472285 | 2 | 0 | 2 |
| yri1066 | NA19200 | chrX | 91086005 | 91109766 | 2 | 1 | 1 |
| yri189 | NA18508 | chr4 | 70471691 | 70542965 | 2 | 0 | 2 |
| yri184 | NA18508 | chr12 | 47011045 | 47020876 | 2 | 2 | 0 |
| yri558 | NA19099 | chr6 | 103784468 | 103799771 | 2 | 2 | 0 |
| yri560 | NA19099 | chr7 | 141456537 | 141472285 | 2 | 1 | 1 |
| yri561 | NA19099 | chr7 | 141462154 | 141472285 | 2 | 1 | 1 |
| yri652 | NA19119 | chr6 | 103784468 | 103799771 | 2 | 2 | 0 |
| yri920 | NA19153 | chrX | 107662335 | 107675738 | 2 | 0 | 2 |
| yri1253 | NA19222 | chr7 | 3136864 | 3171352 | 2 | 0 | 2 |
| yri1067 | NA19201 | chr12 | 130625352 | 130629179 | 2 | 0 | 2 |
| yri910 | NA19152 | chr4 | 9969524 | 9980122 | 2 | 1 | 1 |
| yri1007 | NA19172 | chr21 | 9979029 | 10012221 | 2 | 0 | 2 |
| yri1257 | NA19223 | chr2 | 203499611 | 203511609 | 2 | 2 | 0 |
| yri943 | NA19159 | chrX | 91086005 | 91109766 | 2 | 1 | 1 |
| yri366 | NA18861 | chr3 | 163833596 | 163943569 | 2 | 0 | 2 |
| yri409 | NA18870 | chr7 | 141456537 | 141472285 | 2 | 1 | 1 |
| yri410 | NA18870 | chr7 | 141462154 | 141472285 | 2 | 1 | 1 |
| yri498 | NA19092 | chr21 | 9979029 | 10012221 | 2 | 1 | 1 |
| yri1207 | NA19209 | chr8 | 2242110 | 2250519 | 2 | 0 | 2 |
| ceu807 | NA12005 | chr3 | 163840486 | 163939798 | 1 | 1 | 0 |
| ceu818 | NA12005 | chr8 | 39271742 | 39390071 | 1 | 1 | 0 |
| ceu257 | NA07357 | chr21 | 9979029 | 10016793 | 1 | 1 | 0 |
| ceu261 | NA07357 | chr3 | 163840486 | 163939798 | 1 | 0 | 1 |
| ceu263 | NA07357 | chr3 | 163875766 | 163940699 | 1 | 0 | 1 |
| ceu264 | NA07357 | chr3 | 163882205 | 163926256 | 1 | 0 | 1 |
| ceu270 | NA07357 | chr7 | 141456537 | 141472512 | 1 | 1 | 0 |
| ceu271 | NA07357 | chr7 | 141462154 | 141472512 | 1 | 1 | 0 |
| ceu856 | NA12044 | chr22 | 21268707 | 21396778 | 1 | 1 | 0 |
| ceu1125 | NA12716 | chr8 | 39250107 | 39397764 | 1 | 1 | 0 |
| ceu1126 | NA12716 | chr8 | 39268398 | 39389812 | 1 | 1 | 0 |
| ceu63 | NA06994 | chr22 | 21116954 | 21282684 | 1 | 0 | 1 |
| ceu64 | NA06994 | chr22 | 21118175 | 21360293 | 1 | 0 | 1 |
| ceu66 | NA06994 | chr22 | 21396778 | 21538141 | 1 | 1 | 0 |
| ceu1423 | NA12872 | chr2 | 89093935 | 89175498 | 1 | 1 | 0 |
| ceu1325 | NA12812 | chr3 | 52991144 | 52995043 | 1 | 1 | 0 |
| ceu1333 | NA12812 | chr7 | 89422556 | 89424158 | 1 | 1 | 0 |
| ceu1323 | NA12812 | chr2 | 34688262 | 34696094 | 1 | 1 | 0 |
| ceu955 | NA12154 | chr8 | 39250107 | 39397764 | 1 | 1 | 0 |
| ceu956 | NA12154 | chr8 | 39268398 | 39389812 | 1 | 1 | 0 |
| ceu85 | NA07000 | chr3 | 163835144 | 163922881 | 1 | 1 | 0 |
| ceu86 | NA07000 | chr3 | 163837185 | 163917427 | 1 | 1 | 0 |
| ceu87 | NA07000 | chr3 | 163840486 | 163939798 | 1 | 1 | 0 |
| ceu449 | NA10851 | chr3 | 180609192 | 180611006 | 1 | 1 | 0 |
| ceu452 | NA10851 | chr4 | 69378123 | 69808237 | 1 | 1 | 0 |
| ceu459 | NA10851 | chr7 | 109003350 | 109007346 | 1 | 0 | 1 |
| ceu844 | NA12043 | chr4 | 69441695 | 69482361 | 1 | 0 | 1 |
| ceu836 | NA12043 | chr10 | 41640549 | 41649682 | 1 | 0 | 1 |
| ceu217 | NA07056 | chr2 | 52726065 | 52756887 | 1 | 0 | 1 |
| ceu668 | NA11832 | chr12 | 131823562 | 131838245 | 1 | 1 | 0 |
| ceu721 | NA11992 | chr3 | 60783767 | 60860449 | 1 | 0 | 1 |
| ceu722 | NA11992 | chr3 | 60806084 | 60844635 | 1 | 0 | 1 |
| ceu723 | NA11992 | chr3 | 60816034 | 60842631 | 1 | 0 | 1 |
| ceu1166 | NA12750 | chr4 | 69441695 | 69482361 | 1 | 0 | 1 |
| ceu1179 | NA12751 | chr3 | 163840486 | 163939798 | 1 | 1 | 0 |
| ceu1254 | NA12762 | chr7 | 141456537 | 141472512 | 1 | 0 | 1 |
| ceu1267 | NA12763 | chr7 | 133203070 | 133212391 | 1 | 1 | 0 |
| ceu1356 | NA12814 | chr1 | 142902233 | 142921305 | 1 | 1 | 0 |
| ceu1375 | NA12815 | chr7 | 141456537 | 141472512 | 1 | 1 | 0 |
| ceu1376 | NA12815 | chr7 | 141462154 | 141472512 | 1 | 1 | 0 |
| yri1172 | NA19207 | chr3 | 163833596 | 163943569 | 1 | 0 | 1 |
| yri1178 | NA19207 | chr9 | 43348537 | 43374214 | 1 | 1 | 0 |
| yri543 | NA19098 | chr4 | 69432417 | 69486334 | 1 | 0 | 1 |
| yri862 | NA19143 | chr12 | 27540053 | 27545038 | 1 | 1 | 0 |
| yri858 | NA19143 | chr1 | 72137668 | 72147489 | 1 | 1 | 0 |
| yri868 | NA19143 | chr4 | 69460790 | 69486227 | 1 | 1 | 0 |
| yri869 | NA19143 | chr4 | 69482361 | 69491890 | 1 | 0 | 1 |
| yri783 | NA19137 | chr8 | 2242110 | 2250519 | 1 | 1 | 0 |
| yri711 | NA19129 | chr7 | 89422556 | 89424327 | 1 | 1 | 0 |
| yri708 | NA19129 | chr4 | 70471691 | 70542965 | 1 | 0 | 1 |
| yri731 | NA19130 | chr7 | 141456537 | 141472285 | 1 | 1 | 0 |
| yri732 | NA19130 | chr7 | 141462154 | 141472285 | 1 | 1 | 0 |
| yri139 | NA18505 | chr9 | 5102519 | 5103577 | 1 | 1 | 0 |
| yri131 | NA18505 | chr22 | 22269923 | 22271906 | 1 | 1 | 0 |
| yri117 | NA18505 | chr1 | 94609885 | 94625063 | 1 | 0 | 1 |
| yri135 | NA18505 | chr4 | 108651560 | 108665451 | 1 | 1 | 0 |
| yri1058 | NA19200 | chr3 | 156501087 | 156505963 | 1 | 1 | 0 |
| yri221 | NA18517 | chr7 | 141456537 | 141472285 | 1 | 1 | 0 |
| yri222 | NA18517 | chr7 | 141462154 | 141472285 | 1 | 1 | 0 |
| yri336 | NA18858 | chr8 | 103010682 | 103011802 | 1 | 0 | 1 |
| yri326 | NA18858 | chr2 | 196183303 | 196184993 | 1 | 1 | 0 |
| yri335 | NA18858 | chr8 | 39250107 | 39326538 | 1 | 0 | 1 |
| yri647 | NA19119 | chr4 | 9969524 | 9980122 | 1 | 1 | 0 |
| yri509 | NA19093 | chr2 | 18160633 | 18171634 | 1 | 0 | 1 |
| yri510 | NA19093 | chr4 | 70471691 | 70542965 | 1 | 0 | 1 |
| yri508 | NA19093 | chr2 | 18156325 | 18177354 | 1 | 0 | 1 |
| yri915 | NA19153 | chr20 | 681314 | 685325 | 1 | 1 | 0 |
| yri917 | NA19153 | chr7 | 141456537 | 141472285 | 1 | 1 | 0 |
| yri55 | NA18502 | chr1 | 149771758 | 149798424 | 1 | 1 | 0 |
| yri57 | NA18502 | chr12 | 11398341 | 11431147 | 1 | 1 | 0 |
| yri65 | NA18502 | chr6 | 103784468 | 103799771 | 1 | 1 | 0 |
| yri945 | NA19160 | chr1 | 149771758 | 149798424 | 1 | 1 | 0 |
| yri946 | NA19160 | chr1 | 149786102 | 149798424 | 1 | 1 | 0 |
| yri956 | NA19160 | chr4 | 69460790 | 69486227 | 1 | 1 | 0 |
| yri957 | NA19160 | chr4 | 70471691 | 70542965 | 1 | 0 | 1 |
| yri837 | NA19141 | chrX | 15964777 | 15971948 | 1 | 0 | 1 |
| yri1070 | NA19201 | chr2 | 203499611 | 203511609 | 1 | 0 | 1 |
| yri605 | NA19102 | chr8 | 14590534 | 14596692 | 1 | 1 | 0 |
| yri442 | NA18912 | chr1 | 55871 | 68941 | 1 | 1 | 0 |
| yri449 | NA18912 | chr4 | 70471691 | 70542965 | 1 | 1 | 0 |
| yri912 | NA19152 | chr8 | 14590534 | 14596692 | 1 | 1 | 0 |
| yri906 | NA19152 | chr12 | 27540053 | 27545038 | 1 | 1 | 0 |
| yri1010 | NA19172 | chr4 | 69460790 | 69486227 | 1 | 0 | 1 |
| yri1013 | NA19172 | chr8 | 55414544 | 55423847 | 1 | 1 | 0 |
| yri940 | NA19159 | chr4 | 21123929 | 21126700 | 1 | 0 | 1 |
| yri369 | NA18861 | chr4 | 12129939 | 12134830 | 1 | 1 | 0 |
| yri406 | NA18870 | chr7 | 109002968 | 109011761 | 1 | 0 | 1 |
| yri407 | NA18870 | chr7 | 109003350 | 109007346 | 1 | 0 | 1 |
| yri411 | NA18870 | chr9 | 1500299 | 1516383 | 1 | 1 | 0 |
| yri503 | NA19092 | chr7 | 78439557 | 78445109 | 1 | 0 | 1 |
| yri499 | NA19092 | chr4 | 9969524 | 9980122 | 1 | 0 | 1 |
| yri501 | NA19092 | chr6 | 33985151 | 33989083 | 1 | 0 | 1 |
| yri641 | NA19116 | chr8 | 141973953 | 141975249 | 1 | 0 | 1 |
| yri628 | NA19116 | chr3 | 163833596 | 163943569 | 1 | 0 | 1 |
| yri630 | NA19116 | chr4 | 138551715 | 138556270 | 1 | 0 | 1 |
| yri640 | NA19116 | chr8 | 95515277 | 95528026 | 1 | 0 | 1 |
| yri1208 | NA19209 | chr8 | 16212027 | 16216726 | 1 | 0 | 1 |
| yri1203 | NA19209 | chr17 | 39893166 | 39898343 | 1 | 0 | 1 |
| yri1205 | NA19209 | chr6 | 54698192 | 54707081 | 1 | 0 | 1 |

**Table S2. Sequence of individual reads overlapping sense strands**

**“ForwardID” - internal ID of reads mapping to forward strand; “Deletion” - internal deletion ID; “Sample” - Sample number; “DChr” - chromosome where deletion is located; “DStart” - deletion start position; “DEnd” - deletion end position; “RStart” - read start position; “Rend” - read end position; “Overlap Forward/ReverseID” - overlapping read ID of the other strand. The value 0 indicates the read is not overlapping any read on the other strand. There are only two reads overlapping on the other strand.**

| ForwardRId | Deletion | Sample | DChr | DStart | DEnd | RStart | REnd | Overlap  ReverseRId |
| --- | --- | --- | --- | --- | --- | --- | --- | --- |
| 1 | ceu807 | NA12005 | chr3 | 163840486 | 163939798 | 163848622 | 163848654 | 0 |
| 2 | ceu818 | NA12005 | chr8 | 39271742 | 39390071 | 39355868 | 39355880 | 0 |
| 3 | ceu1215 | NA12760 | chr2 | 89093935 | 89175498 | 89102514 | 89102549 | 0 |
| 4 | ceu1215 | NA12760 | chr2 | 89093935 | 89175498 | 89101435 | 89101470 | 0 |
| 5 | ceu1215 | NA12760 | chr2 | 89093935 | 89175498 | 89104692 | 89104727 | 0 |
| 6 | ceu1215 | NA12760 | chr2 | 89093935 | 89175498 | 89102081 | 89102116 | 0 |
| 7 | ceu1215 | NA12760 | chr2 | 89093935 | 89175498 | 89107388 | 89107423 | 0 |
| 8 | ceu1215 | NA12760 | chr2 | 89093935 | 89175498 | 89103516 | 89103551 | 0 |
| 9 | ceu1215 | NA12760 | chr2 | 89093935 | 89175498 | 89105904 | 89105939 | 0 |
| 10 | ceu1215 | NA12760 | chr2 | 89093935 | 89175498 | 89099516 | 89099548 | 0 |
| 11 | ceu273 | NA07357 | chr9 | 32991449 | 33014917 | 32991742 | 32991777 | 0 |
| 12 | ceu273 | NA07357 | chr9 | 32991449 | 33014917 | 33007018 | 33007053 | 0 |
| 13 | ceu258 | NA07357 | chr22 | 21118175 | 21360293 | 21247265 | 21247300 | 0 |
| 14 | ceu258 | NA07357 | chr22 | 21118175 | 21360293 | 21166755 | 21166771 | 0 |
| 15 | ceu258 | NA07357 | chr22 | 21118175 | 21360293 | 21222388 | 21222423 | 0 |
| 16 | ceu259 | NA07357 | chr22 | 21396778 | 21538141 | 21523735 | 21523770 | 0 |
| 17 | ceu259 | NA07357 | chr22 | 21396778 | 21538141 | 21426144 | 21426179 | 0 |
| 18 | ceu259 | NA07357 | chr22 | 21396778 | 21538141 | 21425784 | 21425819 | 0 |
| 19 | ceu259 | NA07357 | chr22 | 21396778 | 21538141 | 21425785 | 21425820 | 0 |
| 20 | ceu257 | NA07357 | chr21 | 9979029 | 10016793 | 10012477 | 10012512 | 0 |
| 21 | ceu270 | NA07357 | chr7 | 141456537 | 141472512 | 141471370 | 141471405 | 0 |
| 22 | ceu271 | NA07357 | chr7 | 141462154 | 141472512 | 141471370 | 141471405 | 0 |
| 23 | ceu859 | NA12044 | chr4 | 10148210 | 10151039 | 10150795 | 10150808 | 0 |
| 24 | ceu859 | NA12044 | chr4 | 10148210 | 10151039 | 10150794 | 10150808 | 0 |
| 25 | ceu859 | NA12044 | chr4 | 10148210 | 10151039 | 10150795 | 10150808 | 0 |
| 26 | ceu859 | NA12044 | chr4 | 10148210 | 10151039 | 10150795 | 10150809 | 0 |
| 27 | ceu859 | NA12044 | chr4 | 10148210 | 10151039 | 10150795 | 10150809 | 0 |
| 28 | ceu859 | NA12044 | chr4 | 10148210 | 10151039 | 10150795 | 10150809 | 0 |
| 29 | ceu856 | NA12044 | chr22 | 21268707 | 21396778 | 21308616 | 21308651 | 0 |
| 30 | ceu997 | NA12234 | chr8 | 14647130 | 15337510 | 15256707 | 15256742 | 0 |
| 31 | ceu997 | NA12234 | chr8 | 14647130 | 15337510 | 15256707 | 15256742 | 0 |
| 32 | ceu997 | NA12234 | chr8 | 14647130 | 15337510 | 14846346 | 14846381 | 0 |
| 33 | ceu998 | NA12234 | chr8 | 14650691 | 15336034 | 15256707 | 15256742 | 0 |
| 34 | ceu998 | NA12234 | chr8 | 14650691 | 15336034 | 15256707 | 15256742 | 0 |
| 35 | ceu998 | NA12234 | chr8 | 14650691 | 15336034 | 14846346 | 14846381 | 0 |
| 36 | ceu999 | NA12234 | chr8 | 14978429 | 15392548 | 15256707 | 15256742 | 0 |
| 37 | ceu999 | NA12234 | chr8 | 14978429 | 15392548 | 15256707 | 15256742 | 0 |
| 38 | ceu1119 | NA12716 | chr4 | 69378123 | 69808237 | 69453006 | 69453041 | 0 |
| 39 | ceu1125 | NA12716 | chr8 | 39250107 | 39397764 | 39274411 | 39274446 | 0 |
| 40 | ceu1126 | NA12716 | chr8 | 39268398 | 39389812 | 39274411 | 39274446 | 0 |
| 41 | ceu5 | NA06985 | chr12 | 131823562 | 131838245 | 131825715 | 131825750 | 0 |
| 42 | ceu5 | NA06985 | chr12 | 131823562 | 131838245 | 131827024 | 131827059 | 0 |
| 43 | ceu5 | NA06985 | chr12 | 131823562 | 131838245 | 131829141 | 131829176 | 0 |
| 44 | ceu5 | NA06985 | chr12 | 131823562 | 131838245 | 131825629 | 131825664 | 0 |
| 45 | ceu62 | NA06994 | chr22 | 21104182 | 21129536 | 21110865 | 21110900 | 0 |
| 46 | ceu62 | NA06994 | chr22 | 21104182 | 21129536 | 21111246 | 21111281 | 0 |
| 47 | ceu62 | NA06994 | chr22 | 21104182 | 21129536 | 21111246 | 21111281 | 0 |
| 48 | ceu62 | NA06994 | chr22 | 21104182 | 21129536 | 21111247 | 21111282 | 0 |
| 49 | ceu66 | NA06994 | chr22 | 21396778 | 21538141 | 21423259 | 21423274 | 0 |
| 50 | ceu1438 | NA12872 | chr9 | 81945705 | 81999325 | 81993110 | 81993145 | 0 |
| 51 | ceu1438 | NA12872 | chr9 | 81945705 | 81999325 | 81993110 | 81993145 | 0 |
| 52 | ceu1438 | NA12872 | chr9 | 81945705 | 81999325 | 81993040 | 81993075 | 0 |
| 53 | ceu1423 | NA12872 | chr2 | 89093935 | 89175498 | 89121889 | 89121924 | 0 |
| 54 | ceu660 | NA11831 | chr8 | 39271742 | 39390071 | 39279231 | 39279266 | 0 |
| 55 | ceu1326 | NA12812 | chr3 | 163840486 | 163939798 | 163848622 | 163848654 | 0 |
| 56 | ceu1325 | NA12812 | chr3 | 52991144 | 52995043 | 52994468 | 52994503 | 0 |
| 57 | ceu1333 | NA12812 | chr7 | 89422556 | 89424158 | 89424147 | 89424182 | 0 |
| 58 | ceu1323 | NA12812 | chr2 | 34688262 | 34696094 | 34694624 | 34694659 | 0 |
| 59 | ceu824 | NA12006 | chr22 | 37615466 | 37624865 | 37623174 | 37623209 | 0 |
| 60 | ceu824 | NA12006 | chr22 | 37615466 | 37624865 | 37624691 | 37624726 | 0 |
| 61 | ceu832 | NA12006 | chr8 | 39271742 | 39390071 | 39378108 | 39378143 | 0 |
| 62 | ceu939 | NA12154 | chr1 | 172037243 | 172041015 | 172037602 | 172037637 | 0 |
| 63 | ceu939 | NA12154 | chr1 | 172037243 | 172041015 | 172037602 | 172037637 | 0 |
| 64 | ceu955 | NA12154 | chr8 | 39250107 | 39397764 | 39388386 | 39388421 | 0 |
| 65 | ceu956 | NA12154 | chr8 | 39268398 | 39389812 | 39388386 | 39388421 | 0 |
| 66 | ceu85 | NA07000 | chr3 | 163835144 | 163922881 | 163906512 | 163906547 | 0 |
| 67 | ceu86 | NA07000 | chr3 | 163837185 | 163917427 | 163906512 | 163906547 | 0 |
| 68 | ceu87 | NA07000 | chr3 | 163840486 | 163939798 | 163906512 | 163906547 | 0 |
| 69 | ceu445 | NA10851 | chr3 | 163840486 | 163939798 | 163848622 | 163848654 | 0 |
| 70 | ceu445 | NA10851 | chr3 | 163840486 | 163939798 | 163848622 | 163848654 | 0 |
| 71 | ceu464 | NA10851 | chr8 | 39271742 | 39390071 | 39355956 | 39355968 | 0 |
| 72 | ceu449 | NA10851 | chr3 | 180609192 | 180611006 | 180609353 | 180609384 | 0 |
| 73 | ceu452 | NA10851 | chr4 | 69378123 | 69808237 | 69669041 | 69669076 | 0 |
| 74 | ceu1451 | NA12873 | chr8 | 39250107 | 39397764 | 39357071 | 39357106 | 0 |
| 75 | ceu1451 | NA12873 | chr8 | 39250107 | 39397764 | 39313825 | 39313860 | 0 |
| 76 | ceu1452 | NA12873 | chr8 | 39268398 | 39389812 | 39357071 | 39357106 | 0 |
| 77 | ceu1452 | NA12873 | chr8 | 39268398 | 39389812 | 39313825 | 39313860 | 0 |
| 78 | ceu668 | NA11832 | chr12 | 131823562 | 131838245 | 131830267 | 131830302 | 0 |
| 79 | ceu1179 | NA12751 | chr3 | 163840486 | 163939798 | 163848622 | 163848654 | 0 |
| 80 | ceu1267 | NA12763 | chr7 | 133203070 | 133212391 | 133205648 | 133205679 | 0 |
| 81 | ceu1356 | NA12814 | chr1 | 142902233 | 142921305 | 142903778 | 142903789 | 0 |
| 82 | ceu1375 | NA12815 | chr7 | 141456537 | 141472512 | 141471371 | 141471406 | 0 |
| 83 | ceu1376 | NA12815 | chr7 | 141462154 | 141472512 | 141471371 | 141471406 | 0 |
| 84 | yri1179 | NA19207 | chr9 | 62545375 | 62570383 | 62547331 | 62547348 | 0 |
| 85 | yri1179 | NA19207 | chr9 | 62545375 | 62570383 | 62555415 | 62555444 | 0 |
| 86 | yri1179 | NA19207 | chr9 | 62545375 | 62570383 | 62546548 | 62546570 | 0 |
| 87 | yri1177 | NA19207 | chr9 | 36872141 | 36890817 | 36885998 | 36886009 | 0 |
| 88 | yri1176 | NA19207 | chr8 | 39271742 | 39390862 | 39377263 | 39377276 | 0 |
| 89 | yri1176 | NA19207 | chr8 | 39271742 | 39390862 | 39299177 | 39299194 | 0 |
| 90 | yri1176 | NA19207 | chr8 | 39271742 | 39390862 | 39299176 | 39299194 | 0 |
| 91 | yri1176 | NA19207 | chr8 | 39271742 | 39390862 | 39271811 | 39271824 | 0 |
| 92 | yri1176 | NA19207 | chr8 | 39271742 | 39390862 | 39299177 | 39299194 | 0 |
| 93 | yri1176 | NA19207 | chr8 | 39271742 | 39390862 | 39299177 | 39299193 | 0 |
| 94 | yri1167 | NA19207 | chr1 | 149771758 | 149798424 | 149797043 | 149797056 | 0 |
| 95 | yri1167 | NA19207 | chr1 | 149771758 | 149798424 | 149797043 | 149797056 | 0 |
| 96 | yri1167 | NA19207 | chr1 | 149771758 | 149798424 | 149797044 | 149797056 | 0 |
| 97 | yri1167 | NA19207 | chr1 | 149771758 | 149798424 | 149797043 | 149797056 | 0 |
| 98 | yri1167 | NA19207 | chr1 | 149771758 | 149798424 | 149797043 | 149797056 | 0 |
| 99 | yri1167 | NA19207 | chr1 | 149771758 | 149798424 | 149797043 | 149797056 | 0 |
| 100 | yri1167 | NA19207 | chr1 | 149771758 | 149798424 | 149797043 | 149797056 | 0 |
| 101 | yri1167 | NA19207 | chr1 | 149771758 | 149798424 | 149797042 | 149797056 | 0 |
| 102 | yri1167 | NA19207 | chr1 | 149771758 | 149798424 | 149797043 | 149797056 | 0 |
| 103 | yri1167 | NA19207 | chr1 | 149771758 | 149798424 | 149797043 | 149797056 | 0 |
| 104 | yri1167 | NA19207 | chr1 | 149771758 | 149798424 | 149797043 | 149797056 | 0 |
| 105 | yri1168 | NA19207 | chr1 | 149786102 | 149798424 | 149797043 | 149797056 | 0 |
| 106 | yri1168 | NA19207 | chr1 | 149786102 | 149798424 | 149797043 | 149797056 | 0 |
| 107 | yri1168 | NA19207 | chr1 | 149786102 | 149798424 | 149797044 | 149797056 | 0 |
| 108 | yri1168 | NA19207 | chr1 | 149786102 | 149798424 | 149797043 | 149797056 | 0 |
| 109 | yri1168 | NA19207 | chr1 | 149786102 | 149798424 | 149797043 | 149797056 | 0 |
| 110 | yri1168 | NA19207 | chr1 | 149786102 | 149798424 | 149797043 | 149797056 | 0 |
| 111 | yri1168 | NA19207 | chr1 | 149786102 | 149798424 | 149797043 | 149797056 | 0 |
| 112 | yri1168 | NA19207 | chr1 | 149786102 | 149798424 | 149797042 | 149797056 | 0 |
| 113 | yri1168 | NA19207 | chr1 | 149786102 | 149798424 | 149797043 | 149797056 | 0 |
| 114 | yri1168 | NA19207 | chr1 | 149786102 | 149798424 | 149797043 | 149797056 | 0 |
| 115 | yri1168 | NA19207 | chr1 | 149786102 | 149798424 | 149797043 | 149797056 | 0 |
| 116 | yri1173 | NA19207 | chr4 | 70477074 | 70542965 | 70490076 | 70490089 | 0 |
| 117 | yri1178 | NA19207 | chr9 | 43348537 | 43374214 | 43352558 | 43352587 | 0 |
| 118 | yri542 | NA19098 | chr4 | 34677422 | 34724191 | 34702715 | 34702729 | 0 |
| 119 | yri539 | NA19098 | chr21 | 34400727 | 34401819 | 34401417 | 34401452 | 0 |
| 120 | yri539 | NA19098 | chr21 | 34400727 | 34401819 | 34401044 | 34401079 | 0 |
| 121 | yri534 | NA19098 | chr18 | 64171649 | 64270532 | 64173342 | 64173353 | 0 |
| 122 | yri534 | NA19098 | chr18 | 64171649 | 64270532 | 64191120 | 64191135 | 0 |
| 123 | yri863 | NA19143 | chr12 | 63304111 | 63323750 | 63321163 | 63321183 | 0 |
| 124 | yri863 | NA19143 | chr12 | 63304111 | 63323750 | 63308486 | 63308512 | 0 |
| 125 | yri863 | NA19143 | chr12 | 63304111 | 63323750 | 63305857 | 63305881 | 0 |
| 126 | yri863 | NA19143 | chr12 | 63304111 | 63323750 | 63319370 | 63319399 | 0 |
| 127 | yri863 | NA19143 | chr12 | 63304111 | 63323750 | 63322849 | 63322877 | 0 |
| 128 | yri863 | NA19143 | chr12 | 63304111 | 63323750 | 63309465 | 63309482 | 0 |
| 129 | yri863 | NA19143 | chr12 | 63304111 | 63323750 | 63306236 | 63306259 | 0 |
| 130 | yri863 | NA19143 | chr12 | 63304111 | 63323750 | 63315077 | 63315104 | 0 |
| 131 | yri863 | NA19143 | chr12 | 63304111 | 63323750 | 63304751 | 63304778 | 0 |
| 132 | yri863 | NA19143 | chr12 | 63304111 | 63323750 | 63322848 | 63322874 | 0 |
| 133 | yri863 | NA19143 | chr12 | 63304111 | 63323750 | 63321123 | 63321147 | 0 |
| 134 | yri863 | NA19143 | chr12 | 63304111 | 63323750 | 63305693 | 63305728 | 0 |
| 135 | yri863 | NA19143 | chr12 | 63304111 | 63323750 | 63305562 | 63305589 | 0 |
| 136 | yri863 | NA19143 | chr12 | 63304111 | 63323750 | 63312052 | 63312084 | 0 |
| 137 | yri863 | NA19143 | chr12 | 63304111 | 63323750 | 63311912 | 63311932 | 0 |
| 138 | yri863 | NA19143 | chr12 | 63304111 | 63323750 | 63313153 | 63313179 | 0 |
| 139 | yri863 | NA19143 | chr12 | 63304111 | 63323750 | 63320114 | 63320149 | 0 |
| 140 | yri863 | NA19143 | chr12 | 63304111 | 63323750 | 63322849 | 63322877 | 0 |
| 141 | yri863 | NA19143 | chr12 | 63304111 | 63323750 | 63305222 | 63305251 | 0 |
| 142 | yri863 | NA19143 | chr12 | 63304111 | 63323750 | 63316388 | 63316423 | 0 |
| 143 | yri863 | NA19143 | chr12 | 63304111 | 63323750 | 63322849 | 63322884 | 0 |
| 144 | yri863 | NA19143 | chr12 | 63304111 | 63323750 | 63321532 | 63321555 | 0 |
| 145 | yri863 | NA19143 | chr12 | 63304111 | 63323750 | 63311915 | 63311950 | 0 |
| 146 | yri863 | NA19143 | chr12 | 63304111 | 63323750 | 63306673 | 63306701 | 0 |
| 147 | yri863 | NA19143 | chr12 | 63304111 | 63323750 | 63321595 | 63321630 | 391 |
| 148 | yri863 | NA19143 | chr12 | 63304111 | 63323750 | 63311233 | 63311258 | 0 |
| 149 | yri863 | NA19143 | chr12 | 63304111 | 63323750 | 63320300 | 63320325 | 0 |
| 150 | yri863 | NA19143 | chr12 | 63304111 | 63323750 | 63307392 | 63307427 | 0 |
| 151 | yri863 | NA19143 | chr12 | 63304111 | 63323750 | 63309466 | 63309481 | 0 |
| 152 | yri863 | NA19143 | chr12 | 63304111 | 63323750 | 63309281 | 63309316 | 0 |
| 153 | yri863 | NA19143 | chr12 | 63304111 | 63323750 | 63323688 | 63323723 | 0 |
| 154 | yri863 | NA19143 | chr12 | 63304111 | 63323750 | 63323480 | 63323508 | 0 |
| 155 | yri863 | NA19143 | chr12 | 63304111 | 63323750 | 63316394 | 63316418 | 0 |
| 156 | yri863 | NA19143 | chr12 | 63304111 | 63323750 | 63323471 | 63323490 | 0 |
| 157 | yri859 | NA19143 | chr1 | 149771758 | 149798424 | 149797043 | 149797056 | 0 |
| 158 | yri859 | NA19143 | chr1 | 149771758 | 149798424 | 149797043 | 149797056 | 0 |
| 159 | yri859 | NA19143 | chr1 | 149771758 | 149798424 | 149797043 | 149797056 | 0 |
| 160 | yri859 | NA19143 | chr1 | 149771758 | 149798424 | 149797043 | 149797056 | 0 |
| 161 | yri859 | NA19143 | chr1 | 149771758 | 149798424 | 149797043 | 149797056 | 0 |
| 162 | yri859 | NA19143 | chr1 | 149771758 | 149798424 | 149797041 | 149797056 | 0 |
| 163 | yri859 | NA19143 | chr1 | 149771758 | 149798424 | 149797043 | 149797056 | 0 |
| 164 | yri859 | NA19143 | chr1 | 149771758 | 149798424 | 149797043 | 149797056 | 0 |
| 165 | yri859 | NA19143 | chr1 | 149771758 | 149798424 | 149797043 | 149797056 | 0 |
| 166 | yri859 | NA19143 | chr1 | 149771758 | 149798424 | 149797043 | 149797056 | 0 |
| 167 | yri859 | NA19143 | chr1 | 149771758 | 149798424 | 149797043 | 149797056 | 0 |
| 168 | yri860 | NA19143 | chr1 | 149786102 | 149798424 | 149797043 | 149797056 | 0 |
| 169 | yri860 | NA19143 | chr1 | 149786102 | 149798424 | 149797043 | 149797056 | 0 |
| 170 | yri860 | NA19143 | chr1 | 149786102 | 149798424 | 149797043 | 149797056 | 0 |
| 171 | yri860 | NA19143 | chr1 | 149786102 | 149798424 | 149797043 | 149797056 | 0 |
| 172 | yri860 | NA19143 | chr1 | 149786102 | 149798424 | 149797043 | 149797056 | 0 |
| 173 | yri860 | NA19143 | chr1 | 149786102 | 149798424 | 149797041 | 149797056 | 0 |
| 174 | yri860 | NA19143 | chr1 | 149786102 | 149798424 | 149797043 | 149797056 | 0 |
| 175 | yri860 | NA19143 | chr1 | 149786102 | 149798424 | 149797043 | 149797056 | 0 |
| 176 | yri860 | NA19143 | chr1 | 149786102 | 149798424 | 149797043 | 149797056 | 0 |
| 177 | yri860 | NA19143 | chr1 | 149786102 | 149798424 | 149797043 | 149797056 | 0 |
| 178 | yri860 | NA19143 | chr1 | 149786102 | 149798424 | 149797043 | 149797056 | 0 |
| 179 | yri862 | NA19143 | chr12 | 27540053 | 27545038 | 27544942 | 27544967 | 0 |
| 180 | yri858 | NA19143 | chr1 | 72137668 | 72147489 | 72142781 | 72142811 | 0 |
| 181 | yri868 | NA19143 | chr4 | 69460790 | 69486227 | 69467061 | 69467074 | 0 |
| 182 | yri875 | NA19144 | chr15 | 32437866 | 32525037 | 32504984 | 32504999 | 0 |
| 183 | yri875 | NA19144 | chr15 | 32437866 | 32525037 | 32503531 | 32503555 | 0 |
| 184 | yri875 | NA19144 | chr15 | 32437866 | 32525037 | 32522902 | 32522917 | 0 |
| 185 | yri875 | NA19144 | chr15 | 32437866 | 32525037 | 32502683 | 32502711 | 0 |
| 186 | yri875 | NA19144 | chr15 | 32437866 | 32525037 | 32505274 | 32505299 | 0 |
| 187 | yri875 | NA19144 | chr15 | 32437866 | 32525037 | 32504493 | 32504524 | 0 |
| 188 | yri875 | NA19144 | chr15 | 32437866 | 32525037 | 32503412 | 32503438 | 0 |
| 189 | yri875 | NA19144 | chr15 | 32437866 | 32525037 | 32522902 | 32522916 | 0 |
| 190 | yri875 | NA19144 | chr15 | 32437866 | 32525037 | 32524995 | 32525008 | 0 |
| 191 | yri875 | NA19144 | chr15 | 32437866 | 32525037 | 32503533 | 32503568 | 0 |
| 192 | yri875 | NA19144 | chr15 | 32437866 | 32525037 | 32522902 | 32522917 | 0 |
| 193 | yri875 | NA19144 | chr15 | 32437866 | 32525037 | 32504287 | 32504305 | 0 |
| 194 | yri875 | NA19144 | chr15 | 32437866 | 32525037 | 32523505 | 32523517 | 0 |
| 195 | yri876 | NA19144 | chr22 | 21359787 | 21388825 | 21388046 | 21388081 | 0 |
| 196 | yri876 | NA19144 | chr22 | 21359787 | 21388825 | 21377961 | 21377976 | 0 |
| 197 | yri876 | NA19144 | chr22 | 21359787 | 21388825 | 21387386 | 21387421 | 0 |
| 198 | yri876 | NA19144 | chr22 | 21359787 | 21388825 | 21387925 | 21387956 | 0 |
| 199 | yri876 | NA19144 | chr22 | 21359787 | 21388825 | 21388157 | 21388192 | 0 |
| 200 | yri876 | NA19144 | chr22 | 21359787 | 21388825 | 21388137 | 21388157 | 416 |
| 201 | yri876 | NA19144 | chr22 | 21359787 | 21388825 | 21377961 | 21377976 | 0 |
| 202 | yri876 | NA19144 | chr22 | 21359787 | 21388825 | 21377961 | 21377976 | 0 |
| 203 | yri876 | NA19144 | chr22 | 21359787 | 21388825 | 21387932 | 21387962 | 0 |
| 204 | yri876 | NA19144 | chr22 | 21359787 | 21388825 | 21388158 | 21388178 | 0 |
| 205 | yri778 | NA19137 | chr2 | 89796705 | 90026105 | 89804073 | 89804085 | 0 |
| 206 | yri778 | NA19137 | chr2 | 89796705 | 90026105 | 89798729 | 89798743 | 0 |
| 207 | yri778 | NA19137 | chr2 | 89796705 | 90026105 | 89930502 | 89930516 | 0 |
| 208 | yri778 | NA19137 | chr2 | 89796705 | 90026105 | 89800656 | 89800667 | 0 |
| 209 | yri778 | NA19137 | chr2 | 89796705 | 90026105 | 89800656 | 89800667 | 0 |
| 210 | yri778 | NA19137 | chr2 | 89796705 | 90026105 | 90001976 | 90001987 | 0 |
| 211 | yri778 | NA19137 | chr2 | 89796705 | 90026105 | 89912046 | 89912081 | 0 |
| 212 | yri778 | NA19137 | chr2 | 89796705 | 90026105 | 90015221 | 90015233 | 0 |
| 213 | yri778 | NA19137 | chr2 | 89796705 | 90026105 | 89897419 | 89897454 | 0 |
| 214 | yri778 | NA19137 | chr2 | 89796705 | 90026105 | 89798728 | 89798743 | 0 |
| 215 | yri778 | NA19137 | chr2 | 89796705 | 90026105 | 89800656 | 89800667 | 0 |
| 216 | yri778 | NA19137 | chr2 | 89796705 | 90026105 | 89812125 | 89812139 | 0 |
| 217 | yri778 | NA19137 | chr2 | 89796705 | 90026105 | 89912490 | 89912519 | 0 |
| 218 | yri778 | NA19137 | chr2 | 89796705 | 90026105 | 89798728 | 89798742 | 0 |
| 219 | yri778 | NA19137 | chr2 | 89796705 | 90026105 | 89890895 | 89890906 | 0 |
| 220 | yri778 | NA19137 | chr2 | 89796705 | 90026105 | 89970046 | 89970062 | 0 |
| 221 | yri778 | NA19137 | chr2 | 89796705 | 90026105 | 89912490 | 89912519 | 0 |
| 222 | yri778 | NA19137 | chr2 | 89796705 | 90026105 | 89919641 | 89919652 | 0 |
| 223 | yri777 | NA19137 | chr12 | 32414722 | 32422479 | 32417294 | 32417317 | 0 |
| 224 | yri783 | NA19137 | chr8 | 2242110 | 2250519 | 2245675 | 2245688 | 0 |
| 225 | yri702 | NA19129 | chr1 | 149785060 | 149797102 | 149797043 | 149797056 | 0 |
| 226 | yri702 | NA19129 | chr1 | 149785060 | 149797102 | 149797043 | 149797056 | 0 |
| 227 | yri702 | NA19129 | chr1 | 149785060 | 149797102 | 149797043 | 149797056 | 0 |
| 228 | yri702 | NA19129 | chr1 | 149785060 | 149797102 | 149797043 | 149797056 | 0 |
| 229 | yri702 | NA19129 | chr1 | 149785060 | 149797102 | 149797043 | 149797056 | 0 |
| 230 | yri702 | NA19129 | chr1 | 149785060 | 149797102 | 149797043 | 149797056 | 0 |
| 231 | yri702 | NA19129 | chr1 | 149785060 | 149797102 | 149797043 | 149797056 | 0 |
| 232 | yri702 | NA19129 | chr1 | 149785060 | 149797102 | 149797043 | 149797056 | 0 |
| 233 | yri702 | NA19129 | chr1 | 149785060 | 149797102 | 149797043 | 149797056 | 0 |
| 234 | yri702 | NA19129 | chr1 | 149785060 | 149797102 | 149797043 | 149797056 | 0 |
| 235 | yri702 | NA19129 | chr1 | 149785060 | 149797102 | 149797043 | 149797056 | 0 |
| 236 | yri702 | NA19129 | chr1 | 149785060 | 149797102 | 149797043 | 149797056 | 0 |
| 237 | yri702 | NA19129 | chr1 | 149785060 | 149797102 | 149797043 | 149797056 | 0 |
| 238 | yri702 | NA19129 | chr1 | 149785060 | 149797102 | 149797043 | 149797056 | 0 |
| 239 | yri702 | NA19129 | chr1 | 149785060 | 149797102 | 149797043 | 149797056 | 0 |
| 240 | yri702 | NA19129 | chr1 | 149785060 | 149797102 | 149797042 | 149797056 | 0 |
| 241 | yri702 | NA19129 | chr1 | 149785060 | 149797102 | 149797043 | 149797056 | 0 |
| 242 | yri702 | NA19129 | chr1 | 149785060 | 149797102 | 149797043 | 149797056 | 0 |
| 243 | yri702 | NA19129 | chr1 | 149785060 | 149797102 | 149797043 | 149797056 | 0 |
| 244 | yri702 | NA19129 | chr1 | 149785060 | 149797102 | 149797043 | 149797056 | 0 |
| 245 | yri702 | NA19129 | chr1 | 149785060 | 149797102 | 149797043 | 149797056 | 0 |
| 246 | yri702 | NA19129 | chr1 | 149785060 | 149797102 | 149797043 | 149797055 | 0 |
| 247 | yri702 | NA19129 | chr1 | 149785060 | 149797102 | 149797043 | 149797056 | 0 |
| 248 | yri702 | NA19129 | chr1 | 149785060 | 149797102 | 149797043 | 149797056 | 0 |
| 249 | yri702 | NA19129 | chr1 | 149785060 | 149797102 | 149797043 | 149797056 | 0 |
| 250 | yri702 | NA19129 | chr1 | 149785060 | 149797102 | 149797044 | 149797056 | 0 |
| 251 | yri702 | NA19129 | chr1 | 149785060 | 149797102 | 149797043 | 149797056 | 0 |
| 252 | yri702 | NA19129 | chr1 | 149785060 | 149797102 | 149797043 | 149797056 | 0 |
| 253 | yri702 | NA19129 | chr1 | 149785060 | 149797102 | 149797043 | 149797056 | 0 |
| 254 | yri702 | NA19129 | chr1 | 149785060 | 149797102 | 149797043 | 149797056 | 0 |
| 255 | yri702 | NA19129 | chr1 | 149785060 | 149797102 | 149797043 | 149797056 | 0 |
| 256 | yri702 | NA19129 | chr1 | 149785060 | 149797102 | 149797043 | 149797056 | 0 |
| 257 | yri701 | NA19129 | chr1 | 149771758 | 149798424 | 149797043 | 149797056 | 0 |
| 258 | yri701 | NA19129 | chr1 | 149771758 | 149798424 | 149797043 | 149797056 | 0 |
| 259 | yri701 | NA19129 | chr1 | 149771758 | 149798424 | 149797043 | 149797056 | 0 |
| 260 | yri701 | NA19129 | chr1 | 149771758 | 149798424 | 149797043 | 149797056 | 0 |
| 261 | yri701 | NA19129 | chr1 | 149771758 | 149798424 | 149797043 | 149797056 | 0 |
| 262 | yri701 | NA19129 | chr1 | 149771758 | 149798424 | 149797043 | 149797056 | 0 |
| 263 | yri701 | NA19129 | chr1 | 149771758 | 149798424 | 149797043 | 149797056 | 0 |
| 264 | yri701 | NA19129 | chr1 | 149771758 | 149798424 | 149797043 | 149797056 | 0 |
| 265 | yri701 | NA19129 | chr1 | 149771758 | 149798424 | 149797043 | 149797056 | 0 |
| 266 | yri701 | NA19129 | chr1 | 149771758 | 149798424 | 149797043 | 149797056 | 0 |
| 267 | yri701 | NA19129 | chr1 | 149771758 | 149798424 | 149797043 | 149797056 | 0 |
| 268 | yri701 | NA19129 | chr1 | 149771758 | 149798424 | 149797043 | 149797056 | 0 |
| 269 | yri701 | NA19129 | chr1 | 149771758 | 149798424 | 149797043 | 149797056 | 0 |
| 270 | yri701 | NA19129 | chr1 | 149771758 | 149798424 | 149797043 | 149797056 | 0 |
| 271 | yri701 | NA19129 | chr1 | 149771758 | 149798424 | 149797043 | 149797056 | 0 |
| 272 | yri701 | NA19129 | chr1 | 149771758 | 149798424 | 149797042 | 149797056 | 0 |
| 273 | yri701 | NA19129 | chr1 | 149771758 | 149798424 | 149797043 | 149797056 | 0 |
| 274 | yri701 | NA19129 | chr1 | 149771758 | 149798424 | 149797043 | 149797056 | 0 |
| 275 | yri701 | NA19129 | chr1 | 149771758 | 149798424 | 149797043 | 149797056 | 0 |
| 276 | yri701 | NA19129 | chr1 | 149771758 | 149798424 | 149797043 | 149797056 | 0 |
| 277 | yri701 | NA19129 | chr1 | 149771758 | 149798424 | 149797043 | 149797056 | 0 |
| 278 | yri701 | NA19129 | chr1 | 149771758 | 149798424 | 149797043 | 149797055 | 0 |
| 279 | yri701 | NA19129 | chr1 | 149771758 | 149798424 | 149797043 | 149797056 | 0 |
| 280 | yri701 | NA19129 | chr1 | 149771758 | 149798424 | 149797043 | 149797056 | 0 |
| 281 | yri701 | NA19129 | chr1 | 149771758 | 149798424 | 149797043 | 149797056 | 0 |
| 282 | yri701 | NA19129 | chr1 | 149771758 | 149798424 | 149797044 | 149797056 | 0 |
| 283 | yri701 | NA19129 | chr1 | 149771758 | 149798424 | 149797043 | 149797056 | 0 |
| 284 | yri701 | NA19129 | chr1 | 149771758 | 149798424 | 149797043 | 149797056 | 0 |
| 285 | yri701 | NA19129 | chr1 | 149771758 | 149798424 | 149797043 | 149797056 | 0 |
| 286 | yri701 | NA19129 | chr1 | 149771758 | 149798424 | 149797043 | 149797056 | 0 |
| 287 | yri701 | NA19129 | chr1 | 149771758 | 149798424 | 149797043 | 149797056 | 0 |
| 288 | yri701 | NA19129 | chr1 | 149771758 | 149798424 | 149797043 | 149797056 | 0 |
| 289 | yri703 | NA19129 | chr1 | 149786102 | 149798424 | 149797043 | 149797056 | 0 |
| 290 | yri703 | NA19129 | chr1 | 149786102 | 149798424 | 149797043 | 149797056 | 0 |
| 291 | yri703 | NA19129 | chr1 | 149786102 | 149798424 | 149797043 | 149797056 | 0 |
| 292 | yri703 | NA19129 | chr1 | 149786102 | 149798424 | 149797043 | 149797056 | 0 |
| 293 | yri703 | NA19129 | chr1 | 149786102 | 149798424 | 149797043 | 149797056 | 0 |
| 294 | yri703 | NA19129 | chr1 | 149786102 | 149798424 | 149797043 | 149797056 | 0 |
| 295 | yri703 | NA19129 | chr1 | 149786102 | 149798424 | 149797043 | 149797056 | 0 |
| 296 | yri703 | NA19129 | chr1 | 149786102 | 149798424 | 149797043 | 149797056 | 0 |
| 297 | yri703 | NA19129 | chr1 | 149786102 | 149798424 | 149797043 | 149797056 | 0 |
| 298 | yri703 | NA19129 | chr1 | 149786102 | 149798424 | 149797043 | 149797056 | 0 |
| 299 | yri703 | NA19129 | chr1 | 149786102 | 149798424 | 149797043 | 149797056 | 0 |
| 300 | yri703 | NA19129 | chr1 | 149786102 | 149798424 | 149797043 | 149797056 | 0 |
| 301 | yri703 | NA19129 | chr1 | 149786102 | 149798424 | 149797043 | 149797056 | 0 |
| 302 | yri703 | NA19129 | chr1 | 149786102 | 149798424 | 149797043 | 149797056 | 0 |
| 303 | yri703 | NA19129 | chr1 | 149786102 | 149798424 | 149797043 | 149797056 | 0 |
| 304 | yri703 | NA19129 | chr1 | 149786102 | 149798424 | 149797042 | 149797056 | 0 |
| 305 | yri703 | NA19129 | chr1 | 149786102 | 149798424 | 149797043 | 149797056 | 0 |
| 306 | yri703 | NA19129 | chr1 | 149786102 | 149798424 | 149797043 | 149797056 | 0 |
| 307 | yri703 | NA19129 | chr1 | 149786102 | 149798424 | 149797043 | 149797056 | 0 |
| 308 | yri703 | NA19129 | chr1 | 149786102 | 149798424 | 149797043 | 149797056 | 0 |
| 309 | yri703 | NA19129 | chr1 | 149786102 | 149798424 | 149797043 | 149797056 | 0 |
| 310 | yri703 | NA19129 | chr1 | 149786102 | 149798424 | 149797043 | 149797055 | 0 |
| 311 | yri703 | NA19129 | chr1 | 149786102 | 149798424 | 149797043 | 149797056 | 0 |
| 312 | yri703 | NA19129 | chr1 | 149786102 | 149798424 | 149797043 | 149797056 | 0 |
| 313 | yri703 | NA19129 | chr1 | 149786102 | 149798424 | 149797043 | 149797056 | 0 |
| 314 | yri703 | NA19129 | chr1 | 149786102 | 149798424 | 149797044 | 149797056 | 0 |
| 315 | yri703 | NA19129 | chr1 | 149786102 | 149798424 | 149797043 | 149797056 | 0 |
| 316 | yri703 | NA19129 | chr1 | 149786102 | 149798424 | 149797043 | 149797056 | 0 |
| 317 | yri703 | NA19129 | chr1 | 149786102 | 149798424 | 149797043 | 149797056 | 0 |
| 318 | yri703 | NA19129 | chr1 | 149786102 | 149798424 | 149797043 | 149797056 | 0 |
| 319 | yri703 | NA19129 | chr1 | 149786102 | 149798424 | 149797043 | 149797056 | 0 |
| 320 | yri703 | NA19129 | chr1 | 149786102 | 149798424 | 149797043 | 149797056 | 0 |
| 321 | yri711 | NA19129 | chr7 | 89422556 | 89424327 | 89423123 | 89423136 | 0 |
| 322 | yri729 | NA19130 | chr6 | 103787052 | 103807031 | 103797346 | 103797361 | 0 |
| 323 | yri729 | NA19130 | chr6 | 103787052 | 103807031 | 103797344 | 103797359 | 0 |
| 324 | yri729 | NA19130 | chr6 | 103787052 | 103807031 | 103797346 | 103797361 | 0 |
| 325 | yri729 | NA19130 | chr6 | 103787052 | 103807031 | 103797346 | 103797361 | 0 |
| 326 | yri729 | NA19130 | chr6 | 103787052 | 103807031 | 103797344 | 103797362 | 0 |
| 327 | yri729 | NA19130 | chr6 | 103787052 | 103807031 | 103797346 | 103797361 | 0 |
| 328 | yri729 | NA19130 | chr6 | 103787052 | 103807031 | 103797346 | 103797360 | 0 |
| 329 | yri729 | NA19130 | chr6 | 103787052 | 103807031 | 103797346 | 103797361 | 0 |
| 330 | yri729 | NA19130 | chr6 | 103787052 | 103807031 | 103797346 | 103797361 | 0 |
| 331 | yri729 | NA19130 | chr6 | 103787052 | 103807031 | 103797344 | 103797362 | 0 |
| 332 | yri729 | NA19130 | chr6 | 103787052 | 103807031 | 103797344 | 103797359 | 0 |
| 333 | yri729 | NA19130 | chr6 | 103787052 | 103807031 | 103797346 | 103797362 | 0 |
| 334 | yri729 | NA19130 | chr6 | 103787052 | 103807031 | 103797346 | 103797361 | 0 |
| 335 | yri729 | NA19130 | chr6 | 103787052 | 103807031 | 103797344 | 103797360 | 0 |
| 336 | yri729 | NA19130 | chr6 | 103787052 | 103807031 | 103797346 | 103797361 | 0 |
| 337 | yri729 | NA19130 | chr6 | 103787052 | 103807031 | 103797346 | 103797361 | 0 |
| 338 | yri729 | NA19130 | chr6 | 103787052 | 103807031 | 103797346 | 103797361 | 0 |
| 339 | yri729 | NA19130 | chr6 | 103787052 | 103807031 | 103797347 | 103797361 | 0 |
| 340 | yri729 | NA19130 | chr6 | 103787052 | 103807031 | 103797346 | 103797362 | 0 |
| 341 | yri729 | NA19130 | chr6 | 103787052 | 103807031 | 103797345 | 103797361 | 0 |
| 342 | yri729 | NA19130 | chr6 | 103787052 | 103807031 | 103797346 | 103797360 | 0 |
| 343 | yri729 | NA19130 | chr6 | 103787052 | 103807031 | 103797346 | 103797361 | 0 |
| 344 | yri736 | NA19130 | chr8 | 115126252 | 115130784 | 115128925 | 115128936 | 0 |
| 345 | yri736 | NA19130 | chr8 | 115126252 | 115130784 | 115129438 | 115129460 | 0 |
| 346 | yri727 | NA19130 | chr3 | 127035541 | 127042413 | 127036970 | 127036984 | 0 |
| 347 | yri728 | NA19130 | chr4 | 108651560 | 108665451 | 108662275 | 108662290 | 0 |
| 348 | yri731 | NA19130 | chr7 | 141456537 | 141472285 | 141463392 | 141463403 | 0 |
| 349 | yri732 | NA19130 | chr7 | 141462154 | 141472285 | 141463392 | 141463403 | 0 |
| 350 | yri126 | NA18505 | chr2 | 89796705 | 90026105 | 89836589 | 89836611 | 0 |
| 351 | yri126 | NA18505 | chr2 | 89796705 | 90026105 | 89874724 | 89874735 | 0 |
| 352 | yri126 | NA18505 | chr2 | 89796705 | 90026105 | 89970048 | 89970065 | 0 |
| 353 | yri126 | NA18505 | chr2 | 89796705 | 90026105 | 89836579 | 89836605 | 0 |
| 354 | yri126 | NA18505 | chr2 | 89796705 | 90026105 | 89836094 | 89836120 | 0 |
| 355 | yri126 | NA18505 | chr2 | 89796705 | 90026105 | 89836092 | 89836115 | 0 |
| 356 | yri126 | NA18505 | chr2 | 89796705 | 90026105 | 89825025 | 89825036 | 0 |
| 357 | yri126 | NA18505 | chr2 | 89796705 | 90026105 | 90012531 | 90012546 | 0 |
| 358 | yri126 | NA18505 | chr2 | 89796705 | 90026105 | 89836200 | 89836222 | 0 |
| 359 | yri126 | NA18505 | chr2 | 89796705 | 90026105 | 89836589 | 89836611 | 0 |
| 360 | yri126 | NA18505 | chr2 | 89796705 | 90026105 | 89870794 | 89870806 | 0 |
| 361 | yri126 | NA18505 | chr2 | 89796705 | 90026105 | 89866817 | 89866832 | 0 |
| 362 | yri126 | NA18505 | chr2 | 89796705 | 90026105 | 90011611 | 90011635 | 0 |
| 363 | yri126 | NA18505 | chr2 | 89796705 | 90026105 | 89993987 | 89994000 | 0 |
| 364 | yri126 | NA18505 | chr2 | 89796705 | 90026105 | 89834855 | 89834868 | 0 |
| 365 | yri126 | NA18505 | chr2 | 89796705 | 90026105 | 89836085 | 89836099 | 0 |
| 366 | yri126 | NA18505 | chr2 | 89796705 | 90026105 | 89836093 | 89836121 | 0 |
| 367 | yri126 | NA18505 | chr2 | 89796705 | 90026105 | 89970051 | 89970065 | 0 |
| 368 | yri126 | NA18505 | chr2 | 89796705 | 90026105 | 89970029 | 89970042 | 0 |
| 369 | yri126 | NA18505 | chr2 | 89796705 | 90026105 | 89836108 | 89836143 | 0 |
| 370 | yri128 | NA18505 | chr2 | 89826086 | 89981417 | 89836589 | 89836611 | 0 |
| 371 | yri128 | NA18505 | chr2 | 89826086 | 89981417 | 89874724 | 89874735 | 0 |
| 372 | yri128 | NA18505 | chr2 | 89826086 | 89981417 | 89970048 | 89970065 | 0 |
| 373 | yri128 | NA18505 | chr2 | 89826086 | 89981417 | 89836579 | 89836605 | 0 |
| 374 | yri128 | NA18505 | chr2 | 89826086 | 89981417 | 89836094 | 89836120 | 0 |
| 375 | yri128 | NA18505 | chr2 | 89826086 | 89981417 | 89836092 | 89836115 | 0 |
| 376 | yri128 | NA18505 | chr2 | 89826086 | 89981417 | 89836200 | 89836222 | 0 |
| 377 | yri128 | NA18505 | chr2 | 89826086 | 89981417 | 89836589 | 89836611 | 0 |
| 378 | yri128 | NA18505 | chr2 | 89826086 | 89981417 | 89870794 | 89870806 | 0 |
| 379 | yri128 | NA18505 | chr2 | 89826086 | 89981417 | 89866817 | 89866832 | 0 |
| 380 | yri128 | NA18505 | chr2 | 89826086 | 89981417 | 89834855 | 89834868 | 0 |
| 381 | yri128 | NA18505 | chr2 | 89826086 | 89981417 | 89836085 | 89836099 | 0 |
| 382 | yri128 | NA18505 | chr2 | 89826086 | 89981417 | 89836093 | 89836121 | 0 |
| 383 | yri128 | NA18505 | chr2 | 89826086 | 89981417 | 89970051 | 89970065 | 0 |
| 384 | yri128 | NA18505 | chr2 | 89826086 | 89981417 | 89970029 | 89970042 | 0 |
| 385 | yri128 | NA18505 | chr2 | 89826086 | 89981417 | 89836108 | 89836143 | 0 |
| 386 | yri127 | NA18505 | chr2 | 89803197 | 89848524 | 89836589 | 89836611 | 0 |
| 387 | yri127 | NA18505 | chr2 | 89803197 | 89848524 | 89836579 | 89836605 | 0 |
| 388 | yri127 | NA18505 | chr2 | 89803197 | 89848524 | 89836094 | 89836120 | 0 |
| 389 | yri127 | NA18505 | chr2 | 89803197 | 89848524 | 89836092 | 89836115 | 0 |
| 390 | yri127 | NA18505 | chr2 | 89803197 | 89848524 | 89825025 | 89825036 | 0 |
| 391 | yri127 | NA18505 | chr2 | 89803197 | 89848524 | 89836200 | 89836222 | 0 |
| 392 | yri127 | NA18505 | chr2 | 89803197 | 89848524 | 89836589 | 89836611 | 0 |
| 393 | yri127 | NA18505 | chr2 | 89803197 | 89848524 | 89834855 | 89834868 | 0 |
| 394 | yri127 | NA18505 | chr2 | 89803197 | 89848524 | 89836085 | 89836099 | 0 |
| 395 | yri127 | NA18505 | chr2 | 89803197 | 89848524 | 89836093 | 89836121 | 0 |
| 396 | yri127 | NA18505 | chr2 | 89803197 | 89848524 | 89836108 | 89836143 | 0 |
| 397 | yri120 | NA18505 | chr1 | 149785060 | 149797102 | 149797043 | 149797056 | 0 |
| 398 | yri120 | NA18505 | chr1 | 149785060 | 149797102 | 149797041 | 149797056 | 0 |
| 399 | yri120 | NA18505 | chr1 | 149785060 | 149797102 | 149797043 | 149797056 | 0 |
| 400 | yri120 | NA18505 | chr1 | 149785060 | 149797102 | 149797043 | 149797056 | 0 |
| 401 | yri120 | NA18505 | chr1 | 149785060 | 149797102 | 149797043 | 149797056 | 0 |
| 402 | yri120 | NA18505 | chr1 | 149785060 | 149797102 | 149797043 | 149797056 | 0 |
| 403 | yri120 | NA18505 | chr1 | 149785060 | 149797102 | 149797043 | 149797056 | 0 |
| 404 | yri120 | NA18505 | chr1 | 149785060 | 149797102 | 149797041 | 149797056 | 0 |
| 405 | yri120 | NA18505 | chr1 | 149785060 | 149797102 | 149797043 | 149797056 | 0 |
| 406 | yri119 | NA18505 | chr1 | 149771758 | 149798424 | 149797043 | 149797056 | 0 |
| 407 | yri119 | NA18505 | chr1 | 149771758 | 149798424 | 149797041 | 149797056 | 0 |
| 408 | yri119 | NA18505 | chr1 | 149771758 | 149798424 | 149797043 | 149797056 | 0 |
| 409 | yri119 | NA18505 | chr1 | 149771758 | 149798424 | 149797043 | 149797056 | 0 |
| 410 | yri119 | NA18505 | chr1 | 149771758 | 149798424 | 149797043 | 149797056 | 0 |
| 411 | yri119 | NA18505 | chr1 | 149771758 | 149798424 | 149797043 | 149797056 | 0 |
| 412 | yri119 | NA18505 | chr1 | 149771758 | 149798424 | 149797043 | 149797056 | 0 |
| 413 | yri119 | NA18505 | chr1 | 149771758 | 149798424 | 149797041 | 149797056 | 0 |
| 414 | yri119 | NA18505 | chr1 | 149771758 | 149798424 | 149797043 | 149797056 | 0 |
| 415 | yri121 | NA18505 | chr1 | 149786102 | 149798424 | 149797043 | 149797056 | 0 |
| 416 | yri121 | NA18505 | chr1 | 149786102 | 149798424 | 149797041 | 149797056 | 0 |
| 417 | yri121 | NA18505 | chr1 | 149786102 | 149798424 | 149797043 | 149797056 | 0 |
| 418 | yri121 | NA18505 | chr1 | 149786102 | 149798424 | 149797043 | 149797056 | 0 |
| 419 | yri121 | NA18505 | chr1 | 149786102 | 149798424 | 149797043 | 149797056 | 0 |
| 420 | yri121 | NA18505 | chr1 | 149786102 | 149798424 | 149797043 | 149797056 | 0 |
| 421 | yri121 | NA18505 | chr1 | 149786102 | 149798424 | 149797043 | 149797056 | 0 |
| 422 | yri121 | NA18505 | chr1 | 149786102 | 149798424 | 149797041 | 149797056 | 0 |
| 423 | yri121 | NA18505 | chr1 | 149786102 | 149798424 | 149797043 | 149797056 | 0 |
| 424 | yri123 | NA18505 | chr12 | 50791226 | 50819085 | 50799094 | 50799111 | 0 |
| 425 | yri123 | NA18505 | chr12 | 50791226 | 50819085 | 50797906 | 50797921 | 0 |
| 426 | yri123 | NA18505 | chr12 | 50791226 | 50819085 | 50815080 | 50815093 | 0 |
| 427 | yri133 | NA18505 | chr3 | 46758432 | 46807284 | 46764563 | 46764586 | 0 |
| 428 | yri134 | NA18505 | chr3 | 163833596 | 163943569 | 163916843 | 163916855 | 0 |
| 429 | yri139 | NA18505 | chr9 | 5102519 | 5103577 | 5102698 | 5102726 | 0 |
| 430 | yri131 | NA18505 | chr22 | 22269923 | 22271906 | 22270317 | 22270336 | 0 |
| 431 | yri135 | NA18505 | chr4 | 108651560 | 108665451 | 108655467 | 108655481 | 0 |
| 432 | yri1063 | NA19200 | chr6 | 29963788 | 29971727 | 29964309 | 29964327 | 0 |
| 433 | yri1063 | NA19200 | chr6 | 29963788 | 29971727 | 29964310 | 29964327 | 0 |
| 434 | yri1063 | NA19200 | chr6 | 29963788 | 29971727 | 29964507 | 29964542 | 0 |
| 435 | yri1063 | NA19200 | chr6 | 29963788 | 29971727 | 29964624 | 29964644 | 0 |
| 436 | yri1063 | NA19200 | chr6 | 29963788 | 29971727 | 29964053 | 29964078 | 0 |
| 437 | yri1063 | NA19200 | chr6 | 29963788 | 29971727 | 29970264 | 29970276 | 0 |
| 438 | yri1063 | NA19200 | chr6 | 29963788 | 29971727 | 29964308 | 29964325 | 0 |
| 439 | yri1063 | NA19200 | chr6 | 29963788 | 29971727 | 29964047 | 29964064 | 0 |
| 440 | yri1063 | NA19200 | chr6 | 29963788 | 29971727 | 29964271 | 29964290 | 0 |
| 441 | yri1063 | NA19200 | chr6 | 29963788 | 29971727 | 29964314 | 29964331 | 0 |
| 442 | yri1063 | NA19200 | chr6 | 29963788 | 29971727 | 29964309 | 29964325 | 0 |
| 443 | yri1063 | NA19200 | chr6 | 29963788 | 29971727 | 29964048 | 29964064 | 0 |
| 444 | yri1063 | NA19200 | chr6 | 29963788 | 29971727 | 29964306 | 29964326 | 0 |
| 445 | yri1063 | NA19200 | chr6 | 29963788 | 29971727 | 29970073 | 29970108 | 0 |
| 446 | yri1063 | NA19200 | chr6 | 29963788 | 29971727 | 29964304 | 29964325 | 0 |
| 447 | yri1063 | NA19200 | chr6 | 29963788 | 29971727 | 29964305 | 29964327 | 0 |
| 448 | yri1063 | NA19200 | chr6 | 29963788 | 29971727 | 29964309 | 29964326 | 0 |
| 449 | yri1063 | NA19200 | chr6 | 29963788 | 29971727 | 29964311 | 29964329 | 0 |
| 450 | yri1063 | NA19200 | chr6 | 29963788 | 29971727 | 29964312 | 29964329 | 0 |
| 451 | yri1059 | NA19200 | chr3 | 163833596 | 163943569 | 163907627 | 163907642 | 0 |
| 452 | yri1059 | NA19200 | chr3 | 163833596 | 163943569 | 163845808 | 163845821 | 0 |
| 453 | yri1059 | NA19200 | chr3 | 163833596 | 163943569 | 163848622 | 163848654 | 0 |
| 454 | yri1053 | NA19200 | chr1 | 149771758 | 149798424 | 149797043 | 149797056 | 0 |
| 455 | yri1053 | NA19200 | chr1 | 149771758 | 149798424 | 149797043 | 149797056 | 0 |
| 456 | yri1053 | NA19200 | chr1 | 149771758 | 149798424 | 149781698 | 149781725 | 0 |
| 457 | yri1053 | NA19200 | chr1 | 149771758 | 149798424 | 149797043 | 149797056 | 0 |
| 458 | yri1053 | NA19200 | chr1 | 149771758 | 149798424 | 149797044 | 149797056 | 0 |
| 459 | yri1054 | NA19200 | chr1 | 149786102 | 149798424 | 149797043 | 149797056 | 0 |
| 460 | yri1054 | NA19200 | chr1 | 149786102 | 149798424 | 149797043 | 149797056 | 0 |
| 461 | yri1054 | NA19200 | chr1 | 149786102 | 149798424 | 149797043 | 149797056 | 0 |
| 462 | yri1054 | NA19200 | chr1 | 149786102 | 149798424 | 149797044 | 149797056 | 0 |
| 463 | yri1056 | NA19200 | chr10 | 46327384 | 46342622 | 46334442 | 46334474 | 0 |
| 464 | yri1056 | NA19200 | chr10 | 46327384 | 46342622 | 46330068 | 46330103 | 0 |
| 465 | yri1056 | NA19200 | chr10 | 46327384 | 46342622 | 46337172 | 46337204 | 0 |
| 466 | yri1056 | NA19200 | chr10 | 46327384 | 46342622 | 46334813 | 46334826 | 0 |
| 467 | yri1061 | NA19200 | chr4 | 69450972 | 69458490 | 69458204 | 69458231 | 0 |
| 468 | yri1052 | NA19200 | chr1 | 72137668 | 72147489 | 72143725 | 72143738 | 0 |
| 469 | yri1052 | NA19200 | chr1 | 72137668 | 72147489 | 72146839 | 72146854 | 0 |
| 470 | yri1066 | NA19200 | chrX | 91086005 | 91109766 | 91093619 | 91093634 | 0 |
| 471 | yri1058 | NA19200 | chr3 | 156501087 | 156505963 | 156502862 | 156502878 | 0 |
| 472 | yri181 | NA18508 | chr1 | 146563653 | 146572086 | 146571436 | 146571451 | 0 |
| 473 | yri181 | NA18508 | chr1 | 146563653 | 146572086 | 146571436 | 146571451 | 0 |
| 474 | yri181 | NA18508 | chr1 | 146563653 | 146572086 | 146571436 | 146571451 | 0 |
| 475 | yri181 | NA18508 | chr1 | 146563653 | 146572086 | 146571436 | 146571451 | 0 |
| 476 | yri181 | NA18508 | chr1 | 146563653 | 146572086 | 146571436 | 146571451 | 0 |
| 477 | yri181 | NA18508 | chr1 | 146563653 | 146572086 | 146571436 | 146571451 | 0 |
| 478 | yri181 | NA18508 | chr1 | 146563653 | 146572086 | 146571436 | 146571451 | 0 |
| 479 | yri181 | NA18508 | chr1 | 146563653 | 146572086 | 146571436 | 146571451 | 0 |
| 480 | yri181 | NA18508 | chr1 | 146563653 | 146572086 | 146571436 | 146571450 | 0 |
| 481 | yri181 | NA18508 | chr1 | 146563653 | 146572086 | 146571436 | 146571451 | 0 |
| 482 | yri181 | NA18508 | chr1 | 146563653 | 146572086 | 146571436 | 146571450 | 0 |
| 483 | yri181 | NA18508 | chr1 | 146563653 | 146572086 | 146571436 | 146571451 | 0 |
| 484 | yri181 | NA18508 | chr1 | 146563653 | 146572086 | 146571434 | 146571450 | 0 |
| 485 | yri181 | NA18508 | chr1 | 146563653 | 146572086 | 146571436 | 146571451 | 0 |
| 486 | yri181 | NA18508 | chr1 | 146563653 | 146572086 | 146571436 | 146571451 | 0 |
| 487 | yri184 | NA18508 | chr12 | 47011045 | 47020876 | 47013867 | 47013878 | 0 |
| 488 | yri184 | NA18508 | chr12 | 47011045 | 47020876 | 47013867 | 47013878 | 0 |
| 489 | yri548 | NA19099 | chr1 | 146591613 | 146605848 | 146596058 | 146596074 | 0 |
| 490 | yri548 | NA19099 | chr1 | 146591613 | 146605848 | 146593411 | 146593432 | 0 |
| 491 | yri558 | NA19099 | chr6 | 103784468 | 103799771 | 103797346 | 103797361 | 0 |
| 492 | yri558 | NA19099 | chr6 | 103784468 | 103799771 | 103797345 | 103797363 | 0 |
| 493 | yri560 | NA19099 | chr7 | 141456537 | 141472285 | 141470018 | 141470030 | 0 |
| 494 | yri561 | NA19099 | chr7 | 141462154 | 141472285 | 141470018 | 141470030 | 0 |
| 495 | yri738 | NA19131 | chr10 | 54723271 | 54798755 | 54773635 | 54773656 | 0 |
| 496 | yri738 | NA19131 | chr10 | 54723271 | 54798755 | 54777878 | 54777893 | 0 |
| 497 | yri738 | NA19131 | chr10 | 54723271 | 54798755 | 54763748 | 54763783 | 0 |
| 498 | yri746 | NA19131 | chr6 | 103787052 | 103807031 | 103797346 | 103797360 | 0 |
| 499 | yri217 | NA18517 | chr1 | 149771758 | 149798424 | 149781225 | 149781260 | 0 |
| 500 | yri217 | NA18517 | chr1 | 149771758 | 149798424 | 149797043 | 149797057 | 0 |
| 501 | yri217 | NA18517 | chr1 | 149771758 | 149798424 | 149797043 | 149797056 | 0 |
| 502 | yri217 | NA18517 | chr1 | 149771758 | 149798424 | 149797043 | 149797056 | 0 |
| 503 | yri217 | NA18517 | chr1 | 149771758 | 149798424 | 149797043 | 149797056 | 0 |
| 504 | yri217 | NA18517 | chr1 | 149771758 | 149798424 | 149797043 | 149797056 | 0 |
| 505 | yri217 | NA18517 | chr1 | 149771758 | 149798424 | 149797043 | 149797056 | 0 |
| 506 | yri217 | NA18517 | chr1 | 149771758 | 149798424 | 149797043 | 149797056 | 0 |
| 507 | yri217 | NA18517 | chr1 | 149771758 | 149798424 | 149797043 | 149797056 | 0 |
| 508 | yri217 | NA18517 | chr1 | 149771758 | 149798424 | 149797043 | 149797056 | 0 |
| 509 | yri217 | NA18517 | chr1 | 149771758 | 149798424 | 149797043 | 149797056 | 0 |
| 510 | yri217 | NA18517 | chr1 | 149771758 | 149798424 | 149797043 | 149797056 | 0 |
| 511 | yri217 | NA18517 | chr1 | 149771758 | 149798424 | 149797043 | 149797056 | 0 |
| 512 | yri217 | NA18517 | chr1 | 149771758 | 149798424 | 149797043 | 149797056 | 0 |
| 513 | yri218 | NA18517 | chr1 | 149785060 | 149797102 | 149797043 | 149797057 | 0 |
| 514 | yri218 | NA18517 | chr1 | 149785060 | 149797102 | 149797043 | 149797056 | 0 |
| 515 | yri218 | NA18517 | chr1 | 149785060 | 149797102 | 149797043 | 149797056 | 0 |
| 516 | yri218 | NA18517 | chr1 | 149785060 | 149797102 | 149797043 | 149797056 | 0 |
| 517 | yri218 | NA18517 | chr1 | 149785060 | 149797102 | 149797043 | 149797056 | 0 |
| 518 | yri218 | NA18517 | chr1 | 149785060 | 149797102 | 149797043 | 149797056 | 0 |
| 519 | yri218 | NA18517 | chr1 | 149785060 | 149797102 | 149797043 | 149797056 | 0 |
| 520 | yri218 | NA18517 | chr1 | 149785060 | 149797102 | 149797043 | 149797056 | 0 |
| 521 | yri218 | NA18517 | chr1 | 149785060 | 149797102 | 149797043 | 149797056 | 0 |
| 522 | yri218 | NA18517 | chr1 | 149785060 | 149797102 | 149797043 | 149797056 | 0 |
| 523 | yri218 | NA18517 | chr1 | 149785060 | 149797102 | 149797043 | 149797056 | 0 |
| 524 | yri218 | NA18517 | chr1 | 149785060 | 149797102 | 149797043 | 149797056 | 0 |
| 525 | yri218 | NA18517 | chr1 | 149785060 | 149797102 | 149797043 | 149797056 | 0 |
| 526 | yri219 | NA18517 | chr1 | 149786102 | 149798424 | 149797043 | 149797057 | 0 |
| 527 | yri219 | NA18517 | chr1 | 149786102 | 149798424 | 149797043 | 149797056 | 0 |
| 528 | yri219 | NA18517 | chr1 | 149786102 | 149798424 | 149797043 | 149797056 | 0 |
| 529 | yri219 | NA18517 | chr1 | 149786102 | 149798424 | 149797043 | 149797056 | 0 |
| 530 | yri219 | NA18517 | chr1 | 149786102 | 149798424 | 149797043 | 149797056 | 0 |
| 531 | yri219 | NA18517 | chr1 | 149786102 | 149798424 | 149797043 | 149797056 | 0 |
| 532 | yri219 | NA18517 | chr1 | 149786102 | 149798424 | 149797043 | 149797056 | 0 |
| 533 | yri219 | NA18517 | chr1 | 149786102 | 149798424 | 149797043 | 149797056 | 0 |
| 534 | yri219 | NA18517 | chr1 | 149786102 | 149798424 | 149797043 | 149797056 | 0 |
| 535 | yri219 | NA18517 | chr1 | 149786102 | 149798424 | 149797043 | 149797056 | 0 |
| 536 | yri219 | NA18517 | chr1 | 149786102 | 149798424 | 149797043 | 149797056 | 0 |
| 537 | yri219 | NA18517 | chr1 | 149786102 | 149798424 | 149797043 | 149797056 | 0 |
| 538 | yri219 | NA18517 | chr1 | 149786102 | 149798424 | 149797043 | 149797056 | 0 |
| 539 | yri221 | NA18517 | chr7 | 141456537 | 141472285 | 141463384 | 141463403 | 0 |
| 540 | yri222 | NA18517 | chr7 | 141462154 | 141472285 | 141463384 | 141463403 | 0 |
| 541 | yri326 | NA18858 | chr2 | 196183303 | 196184993 | 196184929 | 196184946 | 0 |
| 542 | yri642 | NA19119 | chr1 | 149771758 | 149798424 | 149797043 | 149797056 | 0 |
| 543 | yri642 | NA19119 | chr1 | 149771758 | 149798424 | 149797043 | 149797056 | 0 |
| 544 | yri642 | NA19119 | chr1 | 149771758 | 149798424 | 149797043 | 149797056 | 0 |
| 545 | yri642 | NA19119 | chr1 | 149771758 | 149798424 | 149797043 | 149797056 | 0 |
| 546 | yri642 | NA19119 | chr1 | 149771758 | 149798424 | 149797043 | 149797056 | 0 |
| 547 | yri642 | NA19119 | chr1 | 149771758 | 149798424 | 149797043 | 149797056 | 0 |
| 548 | yri642 | NA19119 | chr1 | 149771758 | 149798424 | 149797044 | 149797056 | 0 |
| 549 | yri643 | NA19119 | chr12 | 11400655 | 11434605 | 11418186 | 11418197 | 0 |
| 550 | yri643 | NA19119 | chr12 | 11400655 | 11434605 | 11433059 | 11433081 | 0 |
| 551 | yri643 | NA19119 | chr12 | 11400655 | 11434605 | 11433300 | 11433327 | 0 |
| 552 | yri652 | NA19119 | chr6 | 103784468 | 103799771 | 103797346 | 103797361 | 0 |
| 553 | yri652 | NA19119 | chr6 | 103784468 | 103799771 | 103797344 | 103797362 | 0 |
| 554 | yri647 | NA19119 | chr4 | 9969524 | 9980122 | 9974708 | 9974720 | 0 |
| 555 | yri511 | NA19093 | chr6 | 29963788 | 29971727 | 29964308 | 29964325 | 0 |
| 556 | yri511 | NA19093 | chr6 | 29963788 | 29971727 | 29964255 | 29964278 | 0 |
| 557 | yri511 | NA19093 | chr6 | 29963788 | 29971727 | 29964047 | 29964072 | 0 |
| 558 | yri511 | NA19093 | chr6 | 29963788 | 29971727 | 29968030 | 29968053 | 0 |
| 559 | yri511 | NA19093 | chr6 | 29963788 | 29971727 | 29966647 | 29966667 | 0 |
| 560 | yri511 | NA19093 | chr6 | 29963788 | 29971727 | 29964038 | 29964059 | 0 |
| 561 | yri511 | NA19093 | chr6 | 29963788 | 29971727 | 29963841 | 29963866 | 0 |
| 562 | yri511 | NA19093 | chr6 | 29963788 | 29971727 | 29964303 | 29964325 | 0 |
| 563 | yri916 | NA19153 | chr4 | 70471691 | 70542965 | 70521146 | 70521157 | 0 |
| 564 | yri914 | NA19153 | chr14 | 74338282 | 74350474 | 74345481 | 74345510 | 0 |
| 565 | yri915 | NA19153 | chr20 | 681314 | 685325 | 683407 | 683421 | 0 |
| 566 | yri917 | NA19153 | chr7 | 141456537 | 141472285 | 141458735 | 141458747 | 0 |
| 567 | yri68 | NA18502 | chrX | 65105136 | 65531010 | 65224861 | 65224893 | 0 |
| 568 | yri68 | NA18502 | chrX | 65105136 | 65531010 | 65349278 | 65349313 | 0 |
| 569 | yri58 | NA18502 | chr14 | 74338282 | 74350474 | 74345218 | 74345241 | 0 |
| 570 | yri58 | NA18502 | chr14 | 74338282 | 74350474 | 74344877 | 74344901 | 0 |
| 571 | yri58 | NA18502 | chr14 | 74338282 | 74350474 | 74344922 | 74344957 | 0 |
| 572 | yri55 | NA18502 | chr1 | 149771758 | 149798424 | 149797043 | 149797056 | 0 |
| 573 | yri57 | NA18502 | chr12 | 11398341 | 11431147 | 11426373 | 11426391 | 0 |
| 574 | yri65 | NA18502 | chr6 | 103784468 | 103799771 | 103797346 | 103797363 | 0 |
| 575 | yri1119 | NA19204 | chr12 | 130253222 | 130299606 | 130268961 | 130268972 | 0 |
| 576 | yri1119 | NA19204 | chr12 | 130253222 | 130299606 | 130259858 | 130259869 | 0 |
| 577 | yri1119 | NA19204 | chr12 | 130253222 | 130299606 | 130268961 | 130268973 | 0 |
| 578 | yri1119 | NA19204 | chr12 | 130253222 | 130299606 | 130293953 | 130293965 | 0 |
| 579 | yri1121 | NA19204 | chr3 | 163833596 | 163943569 | 163902128 | 163902154 | 0 |
| 580 | yri1121 | NA19204 | chr3 | 163833596 | 163943569 | 163923313 | 163923326 | 0 |
| 581 | yri1121 | NA19204 | chr3 | 163833596 | 163943569 | 163891822 | 163891833 | 0 |
| 582 | yri955 | NA19160 | chr4 | 69432417 | 69486334 | 69465182 | 69465213 | 0 |
| 583 | yri949 | NA19160 | chr14 | 74338282 | 74350474 | 74344931 | 74344966 | 0 |
| 584 | yri949 | NA19160 | chr14 | 74338282 | 74350474 | 74344931 | 74344966 | 0 |
| 585 | yri949 | NA19160 | chr14 | 74338282 | 74350474 | 74340754 | 74340789 | 0 |
| 586 | yri945 | NA19160 | chr1 | 149771758 | 149798424 | 149792993 | 149793021 | 0 |
| 587 | yri946 | NA19160 | chr1 | 149786102 | 149798424 | 149792993 | 149793021 | 0 |
| 588 | yri956 | NA19160 | chr4 | 69460790 | 69486227 | 69465182 | 69465213 | 0 |
| 589 | yri831 | NA19141 | chr12 | 22086469 | 22099211 | 22095440 | 22095472 | 0 |
| 590 | yri831 | NA19141 | chr12 | 22086469 | 22099211 | 22090422 | 22090443 | 0 |
| 591 | yri831 | NA19141 | chr12 | 22086469 | 22099211 | 22090392 | 22090403 | 0 |
| 592 | yri831 | NA19141 | chr12 | 22086469 | 22099211 | 22090679 | 22090700 | 0 |
| 593 | yri831 | NA19141 | chr12 | 22086469 | 22099211 | 22092570 | 22092614 | 0 |
| 594 | yri1072 | NA19201 | chr4 | 70471691 | 70542965 | 70518661 | 70518672 | 0 |
| 595 | yri600 | NA19102 | chr20 | 16562202 | 16580314 | 16568647 | 16568661 | 0 |
| 596 | yri600 | NA19102 | chr20 | 16562202 | 16580314 | 16563178 | 16563192 | 0 |
| 597 | yri605 | NA19102 | chr8 | 14590534 | 14596692 | 14595495 | 14595513 | 0 |
| 598 | yri787 | NA19138 | chr2 | 71306841 | 71317129 | 71308173 | 71308185 | 0 |
| 599 | yri788 | NA19138 | chr3 | 163833596 | 163943569 | 163940968 | 163940990 | 0 |
| 600 | yri788 | NA19138 | chr3 | 163833596 | 163943569 | 163839374 | 163839393 | 0 |
| 601 | yri452 | NA18912 | chr8 | 4619513 | 4691949 | 4633145 | 4633167 | 0 |
| 602 | yri452 | NA18912 | chr8 | 4619513 | 4691949 | 4632076 | 4632092 | 0 |
| 603 | yri452 | NA18912 | chr8 | 4619513 | 4691949 | 4632127 | 4632138 | 0 |
| 604 | yri452 | NA18912 | chr8 | 4619513 | 4691949 | 4632303 | 4632324 | 0 |
| 605 | yri442 | NA18912 | chr1 | 55871 | 68941 | 65885 | 65917 | 0 |
| 606 | yri449 | NA18912 | chr4 | 70471691 | 70542965 | 70483608 | 70483627 | 0 |
| 607 | yri908 | NA19152 | chr3 | 46758432 | 46807284 | 46803025 | 46803046 | 0 |
| 608 | yri908 | NA19152 | chr3 | 46758432 | 46807284 | 46768828 | 46768855 | 0 |
| 609 | yri909 | NA19152 | chr3 | 163833596 | 163943569 | 163881504 | 163881520 | 0 |
| 610 | yri910 | NA19152 | chr4 | 9969524 | 9980122 | 9972546 | 9972558 | 0 |
| 611 | yri912 | NA19152 | chr8 | 14590534 | 14596692 | 14590998 | 14591010 | 0 |
| 612 | yri906 | NA19152 | chr12 | 27540053 | 27545038 | 27542569 | 27542586 | 0 |
| 613 | yri1013 | NA19172 | chr8 | 55414544 | 55423847 | 55416496 | 55416508 | 0 |
| 614 | yri1257 | NA19223 | chr2 | 203499611 | 203511609 | 203510583 | 203510613 | 0 |
| 615 | yri1257 | NA19223 | chr2 | 203499611 | 203511609 | 203510583 | 203510613 | 0 |
| 616 | yri1158 | NA19206 | chr2 | 203499611 | 203511609 | 203503358 | 203503393 | 0 |
| 617 | yri1158 | NA19206 | chr2 | 203499611 | 203511609 | 203511362 | 203511397 | 0 |
| 618 | yri1158 | NA19206 | chr2 | 203499611 | 203511609 | 203510020 | 203510055 | 0 |
| 619 | yri941 | NA19159 | chr9 | 11903287 | 11978036 | 11921939 | 11921953 | 0 |
| 620 | yri941 | NA19159 | chr9 | 11903287 | 11978036 | 11927014 | 11927040 | 0 |
| 621 | yri943 | NA19159 | chrX | 91086005 | 91109766 | 91089319 | 91089342 | 0 |
| 622 | yri369 | NA18861 | chr4 | 12129939 | 12134830 | 12134347 | 12134363 | 0 |
| 623 | yri409 | NA18870 | chr7 | 141456537 | 141472285 | 141470024 | 141470035 | 0 |
| 624 | yri410 | NA18870 | chr7 | 141462154 | 141472285 | 141470024 | 141470035 | 0 |
| 625 | yri411 | NA18870 | chr9 | 1500299 | 1516383 | 1503664 | 1503676 | 0 |
| 626 | yri498 | NA19092 | chr21 | 9979029 | 10012221 | 9991307 | 9991320 | 0 |
| 627 | yri1204 | NA19209 | chr3 | 163833596 | 163943569 | 163857491 | 163857505 | 0 |
| 628 | yri1204 | NA19209 | chr3 | 163833596 | 163943569 | 163844277 | 163844294 | 0 |
| 629 | yri1204 | NA19209 | chr3 | 163833596 | 163943569 | 163867745 | 163867758 | 0 |
| 630 | yri1204 | NA19209 | chr3 | 163833596 | 163943569 | 163915490 | 163915503 | 0 |
| 631 | yri1204 | NA19209 | chr3 | 163833596 | 163943569 | 163872363 | 163872380 | 0 |
| 632 | yri1204 | NA19209 | chr3 | 163833596 | 163943569 | 163928964 | 163928979 | 0 |
| 633 | yri1204 | NA19209 | chr3 | 163833596 | 163943569 | 163866267 | 163866278 | 0 |
| 634 | yri1204 | NA19209 | chr3 | 163833596 | 163943569 | 163883327 | 163883339 | 0 |
| 635 | yri1204 | NA19209 | chr3 | 163833596 | 163943569 | 163928964 | 163928979 | 0 |
| 636 | yri1204 | NA19209 | chr3 | 163833596 | 163943569 | 163899467 | 163899484 | 0 |
| 637 | yri1204 | NA19209 | chr3 | 163833596 | 163943569 | 163845808 | 163845821 | 0 |
| 638 | yri1204 | NA19209 | chr3 | 163833596 | 163943569 | 163841516 | 163841527 | 0 |
| 639 | yri1204 | NA19209 | chr3 | 163833596 | 163943569 | 163882612 | 163882623 | 0 |
| 640 | yri1204 | NA19209 | chr3 | 163833596 | 163943569 | 163845808 | 163845821 | 0 |
| 641 | yri1204 | NA19209 | chr3 | 163833596 | 163943569 | 163872363 | 163872380 | 0 |
| 642 | yri1204 | NA19209 | chr3 | 163833596 | 163943569 | 163892423 | 163892435 | 0 |
| 643 | yri1204 | NA19209 | chr3 | 163833596 | 163943569 | 163915490 | 163915503 | 0 |
| 644 | yri1202 | NA19209 | chr12 | 94301826 | 94311594 | 94305312 | 94305324 | 0 |
| 645 | yri1202 | NA19209 | chr12 | 94301826 | 94311594 | 94305313 | 94305324 | 0 |

**Table S3. Sequence of individual reads overlapping antisense strands**

“ReverseID” - internal ID of reads mapping to reverse strand; “Deletion” - internal deletion ID; “Sample” - Sample number; “DChr” - chromosome where deletion is located; “DStart” - deletion start position; “DEnd” - deletion end position; “RStart” - read start position; “Rend” - read end position; “Overlap Reverse/ForwardID” - overlapping read ID of the other strand. The value 0 indicates the read is not overlapping any read on the other strand. There are only two reads overlapping on the other strand.

| ReverseRId | Deletion | Sample | DChr | DStart | DEnd | RStart | REnd | Overlap ForwardRId |
| --- | --- | --- | --- | --- | --- | --- | --- | --- |
| 1 | ceu799 | NA12005 | chr12 | 96517213 | 96533447 | 96518783 | 96518796 | 0 |
| 2 | ceu799 | NA12005 | chr12 | 96517213 | 96533447 | 96518783 | 96518796 | 0 |
| 3 | ceu799 | NA12005 | chr12 | 96517213 | 96533447 | 96518783 | 96518796 | 0 |
| 4 | ceu799 | NA12005 | chr12 | 96517213 | 96533447 | 96518783 | 96518796 | 0 |
| 5 | ceu799 | NA12005 | chr12 | 96517213 | 96533447 | 96518783 | 96518796 | 0 |
| 6 | ceu799 | NA12005 | chr12 | 96517213 | 96533447 | 96518783 | 96518796 | 0 |
| 7 | ceu799 | NA12005 | chr12 | 96517213 | 96533447 | 96518783 | 96518796 | 0 |
| 8 | ceu799 | NA12005 | chr12 | 96517213 | 96533447 | 96518783 | 96518796 | 0 |
| 9 | ceu799 | NA12005 | chr12 | 96517213 | 96533447 | 96518783 | 96518796 | 0 |
| 10 | ceu799 | NA12005 | chr12 | 96517213 | 96533447 | 96518783 | 96518796 | 0 |
| 11 | ceu799 | NA12005 | chr12 | 96517213 | 96533447 | 96518783 | 96518796 | 0 |
| 12 | ceu799 | NA12005 | chr12 | 96517213 | 96533447 | 96518783 | 96518796 | 0 |
| 13 | ceu799 | NA12005 | chr12 | 96517213 | 96533447 | 96518783 | 96518796 | 0 |
| 14 | ceu799 | NA12005 | chr12 | 96517213 | 96533447 | 96518783 | 96518796 | 0 |
| 15 | ceu799 | NA12005 | chr12 | 96517213 | 96533447 | 96518783 | 96518796 | 0 |
| 16 | ceu799 | NA12005 | chr12 | 96517213 | 96533447 | 96518783 | 96518796 | 0 |
| 17 | ceu799 | NA12005 | chr12 | 96517213 | 96533447 | 96518783 | 96518796 | 0 |
| 18 | ceu799 | NA12005 | chr12 | 96517213 | 96533447 | 96518783 | 96518796 | 0 |
| 19 | ceu799 | NA12005 | chr12 | 96517213 | 96533447 | 96518783 | 96518796 | 0 |
| 20 | ceu799 | NA12005 | chr12 | 96517213 | 96533447 | 96518783 | 96518796 | 0 |
| 21 | ceu799 | NA12005 | chr12 | 96517213 | 96533447 | 96518783 | 96518796 | 0 |
| 22 | ceu1215 | NA12760 | chr2 | 89093935 | 89175498 | 89139808 | 89139843 | 0 |
| 23 | ceu1215 | NA12760 | chr2 | 89093935 | 89175498 | 89168141 | 89168176 | 0 |
| 24 | ceu1215 | NA12760 | chr2 | 89093935 | 89175498 | 89127296 | 89127331 | 0 |
| 25 | ceu1215 | NA12760 | chr2 | 89093935 | 89175498 | 89149032 | 89149064 | 0 |
| 26 | ceu1215 | NA12760 | chr2 | 89093935 | 89175498 | 89149416 | 89149451 | 0 |
| 27 | ceu1215 | NA12760 | chr2 | 89093935 | 89175498 | 89146386 | 89146421 | 0 |
| 28 | ceu1215 | NA12760 | chr2 | 89093935 | 89175498 | 89149036 | 89149071 | 0 |
| 29 | ceu1215 | NA12760 | chr2 | 89093935 | 89175498 | 89144982 | 89145017 | 0 |
| 30 | ceu1215 | NA12760 | chr2 | 89093935 | 89175498 | 89145444 | 89145479 | 0 |
| 31 | ceu635 | NA11829 | chr7 | 141921685 | 141931471 | 141928763 | 141928777 | 0 |
| 32 | ceu635 | NA11829 | chr7 | 141921685 | 141931471 | 141928762 | 141928777 | 0 |
| 33 | ceu635 | NA11829 | chr7 | 141921685 | 141931471 | 141928762 | 141928777 | 0 |
| 34 | ceu635 | NA11829 | chr7 | 141921685 | 141931471 | 141928762 | 141928777 | 0 |
| 35 | ceu635 | NA11829 | chr7 | 141921685 | 141931471 | 141928762 | 141928777 | 0 |
| 36 | ceu635 | NA11829 | chr7 | 141921685 | 141931471 | 141928762 | 141928777 | 0 |
| 37 | ceu635 | NA11829 | chr7 | 141921685 | 141931471 | 141928762 | 141928777 | 0 |
| 38 | ceu635 | NA11829 | chr7 | 141921685 | 141931471 | 141928762 | 141928777 | 0 |
| 39 | ceu635 | NA11829 | chr7 | 141921685 | 141931471 | 141928762 | 141928777 | 0 |
| 40 | ceu635 | NA11829 | chr7 | 141921685 | 141931471 | 141928762 | 141928777 | 0 |
| 41 | ceu635 | NA11829 | chr7 | 141921685 | 141931471 | 141928765 | 141928778 | 0 |
| 42 | ceu635 | NA11829 | chr7 | 141921685 | 141931471 | 141928765 | 141928777 | 0 |
| 43 | ceu635 | NA11829 | chr7 | 141921685 | 141931471 | 141928762 | 141928777 | 0 |
| 44 | ceu635 | NA11829 | chr7 | 141921685 | 141931471 | 141928762 | 141928777 | 0 |
| 45 | ceu635 | NA11829 | chr7 | 141921685 | 141931471 | 141928762 | 141928777 | 0 |
| 46 | ceu273 | NA07357 | chr9 | 32991449 | 33014917 | 32996491 | 32996526 | 0 |
| 47 | ceu273 | NA07357 | chr9 | 32991449 | 33014917 | 33007565 | 33007600 | 0 |
| 48 | ceu273 | NA07357 | chr9 | 32991449 | 33014917 | 32998455 | 32998490 | 0 |
| 49 | ceu273 | NA07357 | chr9 | 32991449 | 33014917 | 32997874 | 32997909 | 0 |
| 50 | ceu273 | NA07357 | chr9 | 32991449 | 33014917 | 33011636 | 33011671 | 0 |
| 51 | ceu273 | NA07357 | chr9 | 32991449 | 33014917 | 32992218 | 32992253 | 0 |
| 52 | ceu273 | NA07357 | chr9 | 32991449 | 33014917 | 33006281 | 33006312 | 0 |
| 53 | ceu273 | NA07357 | chr9 | 32991449 | 33014917 | 33003440 | 33003475 | 0 |
| 54 | ceu273 | NA07357 | chr9 | 32991449 | 33014917 | 33000772 | 33000807 | 0 |
| 55 | ceu273 | NA07357 | chr9 | 32991449 | 33014917 | 33013500 | 33013535 | 0 |
| 56 | ceu258 | NA07357 | chr22 | 21118175 | 21360293 | 21121146 | 21121176 | 0 |
| 57 | ceu258 | NA07357 | chr22 | 21118175 | 21360293 | 21334484 | 21334497 | 0 |
| 58 | ceu258 | NA07357 | chr22 | 21118175 | 21360293 | 21334484 | 21334497 | 0 |
| 59 | ceu258 | NA07357 | chr22 | 21118175 | 21360293 | 21334484 | 21334497 | 0 |
| 60 | ceu261 | NA07357 | chr3 | 163840486 | 163939798 | 163888006 | 163888041 | 0 |
| 61 | ceu263 | NA07357 | chr3 | 163875766 | 163940699 | 163888006 | 163888041 | 0 |
| 62 | ceu264 | NA07357 | chr3 | 163882205 | 163926256 | 163888006 | 163888041 | 0 |
| 63 | ceu997 | NA12234 | chr8 | 14647130 | 15337510 | 14726591 | 14726626 | 0 |
| 64 | ceu997 | NA12234 | chr8 | 14647130 | 15337510 | 15032932 | 15032967 | 0 |
| 65 | ceu998 | NA12234 | chr8 | 14650691 | 15336034 | 14726591 | 14726626 | 0 |
| 66 | ceu998 | NA12234 | chr8 | 14650691 | 15336034 | 15032932 | 15032967 | 0 |
| 67 | ceu999 | NA12234 | chr8 | 14978429 | 15392548 | 15032932 | 15032967 | 0 |
| 68 | ceu1119 | NA12716 | chr4 | 69378123 | 69808237 | 69440912 | 69440947 | 0 |
| 69 | ceu1119 | NA12716 | chr4 | 69378123 | 69808237 | 69445409 | 69445439 | 0 |
| 70 | ceu1119 | NA12716 | chr4 | 69378123 | 69808237 | 69445409 | 69445439 | 0 |
| 71 | ceu1119 | NA12716 | chr4 | 69378123 | 69808237 | 69710834 | 69710869 | 0 |
| 72 | ceu63 | NA06994 | chr22 | 21116954 | 21282684 | 21192360 | 21192395 | 0 |
| 73 | ceu64 | NA06994 | chr22 | 21118175 | 21360293 | 21192360 | 21192395 | 0 |
| 74 | ceu1438 | NA12872 | chr9 | 81945705 | 81999325 | 81963494 | 81963529 | 0 |
| 75 | ceu660 | NA11831 | chr8 | 39271742 | 39390071 | 39354677 | 39354712 | 0 |
| 76 | ceu660 | NA11831 | chr8 | 39271742 | 39390071 | 39327035 | 39327070 | 0 |
| 77 | ceu1326 | NA12812 | chr3 | 163840486 | 163939798 | 163928117 | 163928152 | 0 |
| 78 | ceu1326 | NA12812 | chr3 | 163840486 | 163939798 | 163928117 | 163928152 | 0 |
| 79 | ceu832 | NA12006 | chr8 | 39271742 | 39390071 | 39361725 | 39361738 | 0 |
| 80 | ceu82 | NA07000 | chr2 | 89039268 | 89049267 | 89047154 | 89047189 | 0 |
| 81 | ceu82 | NA07000 | chr2 | 89039268 | 89049267 | 89046546 | 89046578 | 0 |
| 82 | ceu464 | NA10851 | chr8 | 39271742 | 39390071 | 39274454 | 39274489 | 0 |
| 83 | ceu459 | NA10851 | chr7 | 109003350 | 109007346 | 109005902 | 109005937 | 0 |
| 84 | ceu1450 | NA12873 | chr4 | 69441695 | 69482361 | 69477963 | 69477998 | 0 |
| 85 | ceu1450 | NA12873 | chr4 | 69441695 | 69482361 | 69445051 | 69445083 | 0 |
| 86 | ceu844 | NA12043 | chr4 | 69441695 | 69482361 | 69442297 | 69442332 | 0 |
| 87 | ceu836 | NA12043 | chr10 | 41640549 | 41649682 | 41644943 | 41644978 | 0 |
| 88 | ceu217 | NA07056 | chr2 | 52726065 | 52756887 | 52727428 | 52727463 | 0 |
| 89 | ceu721 | NA11992 | chr3 | 60783767 | 60860449 | 60832101 | 60832147 | 0 |
| 90 | ceu722 | NA11992 | chr3 | 60806084 | 60844635 | 60832101 | 60832147 | 0 |
| 91 | ceu723 | NA11992 | chr3 | 60816034 | 60842631 | 60832101 | 60832147 | 0 |
| 92 | ceu1166 | NA12750 | chr4 | 69441695 | 69482361 | 69445308 | 69445343 | 0 |
| 93 | ceu1254 | NA12762 | chr7 | 141456537 | 141472512 | 141457737 | 141457750 | 0 |
| 94 | yri1179 | NA19207 | chr9 | 62545375 | 62570383 | 62546937 | 62546950 | 0 |
| 95 | yri1179 | NA19207 | chr9 | 62545375 | 62570383 | 62546937 | 62546957 | 0 |
| 96 | yri1179 | NA19207 | chr9 | 62545375 | 62570383 | 62546937 | 62546954 | 0 |
| 97 | yri1179 | NA19207 | chr9 | 62545375 | 62570383 | 62546939 | 62546964 | 0 |
| 98 | yri1179 | NA19207 | chr9 | 62545375 | 62570383 | 62546937 | 62546951 | 0 |
| 99 | yri1179 | NA19207 | chr9 | 62545375 | 62570383 | 62546938 | 62546955 | 0 |
| 100 | yri1179 | NA19207 | chr9 | 62545375 | 62570383 | 62546937 | 62546957 | 0 |
| 101 | yri1179 | NA19207 | chr9 | 62545375 | 62570383 | 62546938 | 62546954 | 0 |
| 102 | yri1179 | NA19207 | chr9 | 62545375 | 62570383 | 62546937 | 62546956 | 0 |
| 103 | yri1179 | NA19207 | chr9 | 62545375 | 62570383 | 62546937 | 62546956 | 0 |
| 104 | yri1179 | NA19207 | chr9 | 62545375 | 62570383 | 62546937 | 62546956 | 0 |
| 105 | yri1179 | NA19207 | chr9 | 62545375 | 62570383 | 62546937 | 62546956 | 0 |
| 106 | yri1179 | NA19207 | chr9 | 62545375 | 62570383 | 62546937 | 62546957 | 0 |
| 107 | yri1179 | NA19207 | chr9 | 62545375 | 62570383 | 62546937 | 62546952 | 0 |
| 108 | yri1179 | NA19207 | chr9 | 62545375 | 62570383 | 62546937 | 62546956 | 0 |
| 109 | yri1179 | NA19207 | chr9 | 62545375 | 62570383 | 62546937 | 62546952 | 0 |
| 110 | yri1179 | NA19207 | chr9 | 62545375 | 62570383 | 62546937 | 62546956 | 0 |
| 111 | yri1179 | NA19207 | chr9 | 62545375 | 62570383 | 62546937 | 62546956 | 0 |
| 112 | yri1179 | NA19207 | chr9 | 62545375 | 62570383 | 62546937 | 62546950 | 0 |
| 113 | yri1179 | NA19207 | chr9 | 62545375 | 62570383 | 62546937 | 62546956 | 0 |
| 114 | yri1179 | NA19207 | chr9 | 62545375 | 62570383 | 62546937 | 62546954 | 0 |
| 115 | yri1179 | NA19207 | chr9 | 62545375 | 62570383 | 62546937 | 62546956 | 0 |
| 116 | yri1179 | NA19207 | chr9 | 62545375 | 62570383 | 62546937 | 62546955 | 0 |
| 117 | yri1179 | NA19207 | chr9 | 62545375 | 62570383 | 62546938 | 62546953 | 0 |
| 118 | yri1179 | NA19207 | chr9 | 62545375 | 62570383 | 62546937 | 62546956 | 0 |
| 119 | yri1179 | NA19207 | chr9 | 62545375 | 62570383 | 62546937 | 62546952 | 0 |
| 120 | yri1179 | NA19207 | chr9 | 62545375 | 62570383 | 62546937 | 62546955 | 0 |
| 121 | yri1179 | NA19207 | chr9 | 62545375 | 62570383 | 62546937 | 62546954 | 0 |
| 122 | yri1179 | NA19207 | chr9 | 62545375 | 62570383 | 62546937 | 62546956 | 0 |
| 123 | yri1179 | NA19207 | chr9 | 62545375 | 62570383 | 62546937 | 62546955 | 0 |
| 124 | yri1179 | NA19207 | chr9 | 62545375 | 62570383 | 62546938 | 62546951 | 0 |
| 125 | yri1179 | NA19207 | chr9 | 62545375 | 62570383 | 62546938 | 62546951 | 0 |
| 126 | yri1179 | NA19207 | chr9 | 62545375 | 62570383 | 62546937 | 62546954 | 0 |
| 127 | yri1179 | NA19207 | chr9 | 62545375 | 62570383 | 62546937 | 62546968 | 0 |
| 128 | yri1179 | NA19207 | chr9 | 62545375 | 62570383 | 62546937 | 62546956 | 0 |
| 129 | yri1179 | NA19207 | chr9 | 62545375 | 62570383 | 62546937 | 62546953 | 0 |
| 130 | yri1179 | NA19207 | chr9 | 62545375 | 62570383 | 62560599 | 62560634 | 0 |
| 131 | yri1179 | NA19207 | chr9 | 62545375 | 62570383 | 62546937 | 62546954 | 0 |
| 132 | yri1179 | NA19207 | chr9 | 62545375 | 62570383 | 62546937 | 62546956 | 0 |
| 133 | yri1179 | NA19207 | chr9 | 62545375 | 62570383 | 62546938 | 62546949 | 0 |
| 134 | yri1179 | NA19207 | chr9 | 62545375 | 62570383 | 62546937 | 62546954 | 0 |
| 135 | yri1179 | NA19207 | chr9 | 62545375 | 62570383 | 62546938 | 62546956 | 0 |
| 136 | yri1179 | NA19207 | chr9 | 62545375 | 62570383 | 62546937 | 62546955 | 0 |
| 137 | yri1179 | NA19207 | chr9 | 62545375 | 62570383 | 62546937 | 62546949 | 0 |
| 138 | yri1179 | NA19207 | chr9 | 62545375 | 62570383 | 62546939 | 62546954 | 0 |
| 139 | yri1179 | NA19207 | chr9 | 62545375 | 62570383 | 62546937 | 62546952 | 0 |
| 140 | yri1179 | NA19207 | chr9 | 62545375 | 62570383 | 62546937 | 62546956 | 0 |
| 141 | yri1179 | NA19207 | chr9 | 62545375 | 62570383 | 62546937 | 62546956 | 0 |
| 142 | yri1179 | NA19207 | chr9 | 62545375 | 62570383 | 62546937 | 62546956 | 0 |
| 143 | yri1179 | NA19207 | chr9 | 62545375 | 62570383 | 62546937 | 62546956 | 0 |
| 144 | yri1179 | NA19207 | chr9 | 62545375 | 62570383 | 62546937 | 62546956 | 0 |
| 145 | yri1179 | NA19207 | chr9 | 62545375 | 62570383 | 62546937 | 62546961 | 0 |
| 146 | yri1179 | NA19207 | chr9 | 62545375 | 62570383 | 62546937 | 62546952 | 0 |
| 147 | yri1179 | NA19207 | chr9 | 62545375 | 62570383 | 62546882 | 62546917 | 0 |
| 148 | yri1179 | NA19207 | chr9 | 62545375 | 62570383 | 62546937 | 62546954 | 0 |
| 149 | yri1179 | NA19207 | chr9 | 62545375 | 62570383 | 62546937 | 62546951 | 0 |
| 150 | yri1179 | NA19207 | chr9 | 62545375 | 62570383 | 62546937 | 62546956 | 0 |
| 151 | yri1179 | NA19207 | chr9 | 62545375 | 62570383 | 62546937 | 62546952 | 0 |
| 152 | yri1179 | NA19207 | chr9 | 62545375 | 62570383 | 62546937 | 62546951 | 0 |
| 153 | yri1179 | NA19207 | chr9 | 62545375 | 62570383 | 62546938 | 62546957 | 0 |
| 154 | yri1179 | NA19207 | chr9 | 62545375 | 62570383 | 62546938 | 62546952 | 0 |
| 155 | yri1179 | NA19207 | chr9 | 62545375 | 62570383 | 62546938 | 62546955 | 0 |
| 156 | yri1179 | NA19207 | chr9 | 62545375 | 62570383 | 62546937 | 62546955 | 0 |
| 157 | yri1179 | NA19207 | chr9 | 62545375 | 62570383 | 62546937 | 62546950 | 0 |
| 158 | yri1179 | NA19207 | chr9 | 62545375 | 62570383 | 62546937 | 62546954 | 0 |
| 159 | yri1179 | NA19207 | chr9 | 62545375 | 62570383 | 62546937 | 62546951 | 0 |
| 160 | yri1179 | NA19207 | chr9 | 62545375 | 62570383 | 62546937 | 62546955 | 0 |
| 161 | yri1179 | NA19207 | chr9 | 62545375 | 62570383 | 62546872 | 62546898 | 0 |
| 162 | yri1179 | NA19207 | chr9 | 62545375 | 62570383 | 62546937 | 62546956 | 0 |
| 163 | yri1179 | NA19207 | chr9 | 62545375 | 62570383 | 62546937 | 62546956 | 0 |
| 164 | yri1179 | NA19207 | chr9 | 62545375 | 62570383 | 62546938 | 62546953 | 0 |
| 165 | yri1179 | NA19207 | chr9 | 62545375 | 62570383 | 62546937 | 62546956 | 0 |
| 166 | yri1179 | NA19207 | chr9 | 62545375 | 62570383 | 62546937 | 62546955 | 0 |
| 167 | yri1179 | NA19207 | chr9 | 62545375 | 62570383 | 62546937 | 62546956 | 0 |
| 168 | yri1179 | NA19207 | chr9 | 62545375 | 62570383 | 62546937 | 62546956 | 0 |
| 169 | yri1179 | NA19207 | chr9 | 62545375 | 62570383 | 62546937 | 62546952 | 0 |
| 170 | yri1179 | NA19207 | chr9 | 62545375 | 62570383 | 62546937 | 62546957 | 0 |
| 171 | yri1179 | NA19207 | chr9 | 62545375 | 62570383 | 62546937 | 62546953 | 0 |
| 172 | yri1179 | NA19207 | chr9 | 62545375 | 62570383 | 62546937 | 62546950 | 0 |
| 173 | yri1179 | NA19207 | chr9 | 62545375 | 62570383 | 62546937 | 62546956 | 0 |
| 174 | yri1179 | NA19207 | chr9 | 62545375 | 62570383 | 62546937 | 62546950 | 0 |
| 175 | yri1179 | NA19207 | chr9 | 62545375 | 62570383 | 62546938 | 62546956 | 0 |
| 176 | yri1179 | NA19207 | chr9 | 62545375 | 62570383 | 62546937 | 62546956 | 0 |
| 177 | yri1179 | NA19207 | chr9 | 62545375 | 62570383 | 62546931 | 62546966 | 0 |
| 178 | yri1179 | NA19207 | chr9 | 62545375 | 62570383 | 62546937 | 62546956 | 0 |
| 179 | yri1179 | NA19207 | chr9 | 62545375 | 62570383 | 62546938 | 62546954 | 0 |
| 180 | yri1179 | NA19207 | chr9 | 62545375 | 62570383 | 62546937 | 62546955 | 0 |
| 181 | yri1179 | NA19207 | chr9 | 62545375 | 62570383 | 62546937 | 62546957 | 0 |
| 182 | yri1179 | NA19207 | chr9 | 62545375 | 62570383 | 62546938 | 62546956 | 0 |
| 183 | yri1179 | NA19207 | chr9 | 62545375 | 62570383 | 62546937 | 62546954 | 0 |
| 184 | yri1179 | NA19207 | chr9 | 62545375 | 62570383 | 62546937 | 62546955 | 0 |
| 185 | yri1179 | NA19207 | chr9 | 62545375 | 62570383 | 62546937 | 62546956 | 0 |
| 186 | yri1179 | NA19207 | chr9 | 62545375 | 62570383 | 62546937 | 62546954 | 0 |
| 187 | yri1179 | NA19207 | chr9 | 62545375 | 62570383 | 62546937 | 62546956 | 0 |
| 188 | yri1179 | NA19207 | chr9 | 62545375 | 62570383 | 62546937 | 62546956 | 0 |
| 189 | yri1179 | NA19207 | chr9 | 62545375 | 62570383 | 62546938 | 62546953 | 0 |
| 190 | yri1179 | NA19207 | chr9 | 62545375 | 62570383 | 62546937 | 62546951 | 0 |
| 191 | yri1179 | NA19207 | chr9 | 62545375 | 62570383 | 62546937 | 62546957 | 0 |
| 192 | yri1179 | NA19207 | chr9 | 62545375 | 62570383 | 62546937 | 62546953 | 0 |
| 193 | yri1179 | NA19207 | chr9 | 62545375 | 62570383 | 62546937 | 62546956 | 0 |
| 194 | yri1179 | NA19207 | chr9 | 62545375 | 62570383 | 62546937 | 62546957 | 0 |
| 195 | yri1179 | NA19207 | chr9 | 62545375 | 62570383 | 62546937 | 62546962 | 0 |
| 196 | yri1179 | NA19207 | chr9 | 62545375 | 62570383 | 62546937 | 62546956 | 0 |
| 197 | yri1179 | NA19207 | chr9 | 62545375 | 62570383 | 62546937 | 62546962 | 0 |
| 198 | yri1179 | NA19207 | chr9 | 62545375 | 62570383 | 62546937 | 62546953 | 0 |
| 199 | yri1179 | NA19207 | chr9 | 62545375 | 62570383 | 62546937 | 62546957 | 0 |
| 200 | yri1179 | NA19207 | chr9 | 62545375 | 62570383 | 62546937 | 62546951 | 0 |
| 201 | yri1179 | NA19207 | chr9 | 62545375 | 62570383 | 62546846 | 62546863 | 0 |
| 202 | yri1179 | NA19207 | chr9 | 62545375 | 62570383 | 62546937 | 62546949 | 0 |
| 203 | yri1179 | NA19207 | chr9 | 62545375 | 62570383 | 62546937 | 62546956 | 0 |
| 204 | yri1179 | NA19207 | chr9 | 62545375 | 62570383 | 62546937 | 62546956 | 0 |
| 205 | yri1179 | NA19207 | chr9 | 62545375 | 62570383 | 62546937 | 62546957 | 0 |
| 206 | yri1179 | NA19207 | chr9 | 62545375 | 62570383 | 62546937 | 62546954 | 0 |
| 207 | yri1179 | NA19207 | chr9 | 62545375 | 62570383 | 62546937 | 62546956 | 0 |
| 208 | yri1179 | NA19207 | chr9 | 62545375 | 62570383 | 62546937 | 62546960 | 0 |
| 209 | yri1179 | NA19207 | chr9 | 62545375 | 62570383 | 62546937 | 62546956 | 0 |
| 210 | yri1179 | NA19207 | chr9 | 62545375 | 62570383 | 62546938 | 62546954 | 0 |
| 211 | yri1179 | NA19207 | chr9 | 62545375 | 62570383 | 62546937 | 62546955 | 0 |
| 212 | yri1179 | NA19207 | chr9 | 62545375 | 62570383 | 62546938 | 62546954 | 0 |
| 213 | yri1179 | NA19207 | chr9 | 62545375 | 62570383 | 62546937 | 62546955 | 0 |
| 214 | yri1179 | NA19207 | chr9 | 62545375 | 62570383 | 62546937 | 62546952 | 0 |
| 215 | yri1179 | NA19207 | chr9 | 62545375 | 62570383 | 62546937 | 62546955 | 0 |
| 216 | yri1179 | NA19207 | chr9 | 62545375 | 62570383 | 62546937 | 62546956 | 0 |
| 217 | yri1179 | NA19207 | chr9 | 62545375 | 62570383 | 62546937 | 62546956 | 0 |
| 218 | yri1179 | NA19207 | chr9 | 62545375 | 62570383 | 62546937 | 62546956 | 0 |
| 219 | yri1179 | NA19207 | chr9 | 62545375 | 62570383 | 62546937 | 62546960 | 0 |
| 220 | yri1179 | NA19207 | chr9 | 62545375 | 62570383 | 62546937 | 62546953 | 0 |
| 221 | yri1179 | NA19207 | chr9 | 62545375 | 62570383 | 62546937 | 62546957 | 0 |
| 222 | yri1179 | NA19207 | chr9 | 62545375 | 62570383 | 62546937 | 62546950 | 0 |
| 223 | yri1179 | NA19207 | chr9 | 62545375 | 62570383 | 62546937 | 62546956 | 0 |
| 224 | yri1179 | NA19207 | chr9 | 62545375 | 62570383 | 62546937 | 62546956 | 0 |
| 225 | yri1179 | NA19207 | chr9 | 62545375 | 62570383 | 62546937 | 62546956 | 0 |
| 226 | yri1179 | NA19207 | chr9 | 62545375 | 62570383 | 62546937 | 62546951 | 0 |
| 227 | yri1179 | NA19207 | chr9 | 62545375 | 62570383 | 62546937 | 62546960 | 0 |
| 228 | yri1179 | NA19207 | chr9 | 62545375 | 62570383 | 62546937 | 62546951 | 0 |
| 229 | yri1179 | NA19207 | chr9 | 62545375 | 62570383 | 62546937 | 62546956 | 0 |
| 230 | yri1179 | NA19207 | chr9 | 62545375 | 62570383 | 62546937 | 62546955 | 0 |
| 231 | yri1179 | NA19207 | chr9 | 62545375 | 62570383 | 62546937 | 62546953 | 0 |
| 232 | yri1179 | NA19207 | chr9 | 62545375 | 62570383 | 62546937 | 62546957 | 0 |
| 233 | yri1179 | NA19207 | chr9 | 62545375 | 62570383 | 62546937 | 62546957 | 0 |
| 234 | yri1179 | NA19207 | chr9 | 62545375 | 62570383 | 62546937 | 62546952 | 0 |
| 235 | yri1179 | NA19207 | chr9 | 62545375 | 62570383 | 62546937 | 62546956 | 0 |
| 236 | yri1179 | NA19207 | chr9 | 62545375 | 62570383 | 62546937 | 62546956 | 0 |
| 237 | yri1179 | NA19207 | chr9 | 62545375 | 62570383 | 62546937 | 62546956 | 0 |
| 238 | yri1179 | NA19207 | chr9 | 62545375 | 62570383 | 62546937 | 62546952 | 0 |
| 239 | yri1179 | NA19207 | chr9 | 62545375 | 62570383 | 62546937 | 62546958 | 0 |
| 240 | yri1179 | NA19207 | chr9 | 62545375 | 62570383 | 62546937 | 62546952 | 0 |
| 241 | yri1179 | NA19207 | chr9 | 62545375 | 62570383 | 62546937 | 62546956 | 0 |
| 242 | yri1179 | NA19207 | chr9 | 62545375 | 62570383 | 62546937 | 62546954 | 0 |
| 243 | yri1179 | NA19207 | chr9 | 62545375 | 62570383 | 62546937 | 62546956 | 0 |
| 244 | yri1179 | NA19207 | chr9 | 62545375 | 62570383 | 62546937 | 62546954 | 0 |
| 245 | yri1179 | NA19207 | chr9 | 62545375 | 62570383 | 62546939 | 62546952 | 0 |
| 246 | yri1179 | NA19207 | chr9 | 62545375 | 62570383 | 62546937 | 62546956 | 0 |
| 247 | yri1179 | NA19207 | chr9 | 62545375 | 62570383 | 62546931 | 62546966 | 0 |
| 248 | yri1179 | NA19207 | chr9 | 62545375 | 62570383 | 62546937 | 62546951 | 0 |
| 249 | yri1179 | NA19207 | chr9 | 62545375 | 62570383 | 62546298 | 62546330 | 0 |
| 250 | yri1179 | NA19207 | chr9 | 62545375 | 62570383 | 62546937 | 62546958 | 0 |
| 251 | yri1179 | NA19207 | chr9 | 62545375 | 62570383 | 62546937 | 62546953 | 0 |
| 252 | yri1179 | NA19207 | chr9 | 62545375 | 62570383 | 62546937 | 62546950 | 0 |
| 253 | yri1179 | NA19207 | chr9 | 62545375 | 62570383 | 62546937 | 62546957 | 0 |
| 254 | yri1179 | NA19207 | chr9 | 62545375 | 62570383 | 62546937 | 62546956 | 0 |
| 255 | yri1179 | NA19207 | chr9 | 62545375 | 62570383 | 62546937 | 62546956 | 0 |
| 256 | yri1179 | NA19207 | chr9 | 62545375 | 62570383 | 62546937 | 62546956 | 0 |
| 257 | yri1179 | NA19207 | chr9 | 62545375 | 62570383 | 62546937 | 62546957 | 0 |
| 258 | yri1179 | NA19207 | chr9 | 62545375 | 62570383 | 62546937 | 62546956 | 0 |
| 259 | yri1179 | NA19207 | chr9 | 62545375 | 62570383 | 62546938 | 62546949 | 0 |
| 260 | yri1179 | NA19207 | chr9 | 62545375 | 62570383 | 62546937 | 62546952 | 0 |
| 261 | yri1179 | NA19207 | chr9 | 62545375 | 62570383 | 62546851 | 62546883 | 0 |
| 262 | yri1179 | NA19207 | chr9 | 62545375 | 62570383 | 62546937 | 62546954 | 0 |
| 263 | yri1179 | NA19207 | chr9 | 62545375 | 62570383 | 62546937 | 62546956 | 0 |
| 264 | yri1179 | NA19207 | chr9 | 62545375 | 62570383 | 62546937 | 62546955 | 0 |
| 265 | yri1179 | NA19207 | chr9 | 62545375 | 62570383 | 62546937 | 62546955 | 0 |
| 266 | yri1179 | NA19207 | chr9 | 62545375 | 62570383 | 62546937 | 62546954 | 0 |
| 267 | yri1179 | NA19207 | chr9 | 62545375 | 62570383 | 62546937 | 62546956 | 0 |
| 268 | yri1179 | NA19207 | chr9 | 62545375 | 62570383 | 62546937 | 62546950 | 0 |
| 269 | yri1179 | NA19207 | chr9 | 62545375 | 62570383 | 62546927 | 62546959 | 0 |
| 270 | yri1179 | NA19207 | chr9 | 62545375 | 62570383 | 62546937 | 62546956 | 0 |
| 271 | yri1179 | NA19207 | chr9 | 62545375 | 62570383 | 62546937 | 62546955 | 0 |
| 272 | yri1179 | NA19207 | chr9 | 62545375 | 62570383 | 62546937 | 62546950 | 0 |
| 273 | yri1179 | NA19207 | chr9 | 62545375 | 62570383 | 62546937 | 62546956 | 0 |
| 274 | yri1179 | NA19207 | chr9 | 62545375 | 62570383 | 62546940 | 62546955 | 0 |
| 275 | yri1179 | NA19207 | chr9 | 62545375 | 62570383 | 62546938 | 62546955 | 0 |
| 276 | yri1179 | NA19207 | chr9 | 62545375 | 62570383 | 62546937 | 62546956 | 0 |
| 277 | yri1179 | NA19207 | chr9 | 62545375 | 62570383 | 62546937 | 62546955 | 0 |
| 278 | yri1179 | NA19207 | chr9 | 62545375 | 62570383 | 62546939 | 62546955 | 0 |
| 279 | yri1179 | NA19207 | chr9 | 62545375 | 62570383 | 62546871 | 62546888 | 0 |
| 280 | yri1179 | NA19207 | chr9 | 62545375 | 62570383 | 62546937 | 62546956 | 0 |
| 281 | yri1179 | NA19207 | chr9 | 62545375 | 62570383 | 62546938 | 62546955 | 0 |
| 282 | yri1179 | NA19207 | chr9 | 62545375 | 62570383 | 62546937 | 62546954 | 0 |
| 283 | yri1179 | NA19207 | chr9 | 62545375 | 62570383 | 62547011 | 62547043 | 0 |
| 284 | yri1179 | NA19207 | chr9 | 62545375 | 62570383 | 62546937 | 62546954 | 0 |
| 285 | yri1179 | NA19207 | chr9 | 62545375 | 62570383 | 62546938 | 62546949 | 0 |
| 286 | yri1179 | NA19207 | chr9 | 62545375 | 62570383 | 62546937 | 62546952 | 0 |
| 287 | yri1179 | NA19207 | chr9 | 62545375 | 62570383 | 62546937 | 62546956 | 0 |
| 288 | yri1179 | NA19207 | chr9 | 62545375 | 62570383 | 62546937 | 62546952 | 0 |
| 289 | yri1179 | NA19207 | chr9 | 62545375 | 62570383 | 62546937 | 62546956 | 0 |
| 290 | yri1179 | NA19207 | chr9 | 62545375 | 62570383 | 62546938 | 62546950 | 0 |
| 291 | yri1179 | NA19207 | chr9 | 62545375 | 62570383 | 62546938 | 62546963 | 0 |
| 292 | yri1179 | NA19207 | chr9 | 62545375 | 62570383 | 62546938 | 62546953 | 0 |
| 293 | yri1179 | NA19207 | chr9 | 62545375 | 62570383 | 62546937 | 62546955 | 0 |
| 294 | yri1179 | NA19207 | chr9 | 62545375 | 62570383 | 62546937 | 62546952 | 0 |
| 295 | yri1179 | NA19207 | chr9 | 62545375 | 62570383 | 62546871 | 62546888 | 0 |
| 296 | yri1179 | NA19207 | chr9 | 62545375 | 62570383 | 62546937 | 62546957 | 0 |
| 297 | yri1179 | NA19207 | chr9 | 62545375 | 62570383 | 62546937 | 62546956 | 0 |
| 298 | yri1179 | NA19207 | chr9 | 62545375 | 62570383 | 62546937 | 62546955 | 0 |
| 299 | yri1179 | NA19207 | chr9 | 62545375 | 62570383 | 62546937 | 62546953 | 0 |
| 300 | yri1179 | NA19207 | chr9 | 62545375 | 62570383 | 62546937 | 62546955 | 0 |
| 301 | yri1179 | NA19207 | chr9 | 62545375 | 62570383 | 62546937 | 62546950 | 0 |
| 302 | yri1179 | NA19207 | chr9 | 62545375 | 62570383 | 62546937 | 62546955 | 0 |
| 303 | yri1179 | NA19207 | chr9 | 62545375 | 62570383 | 62546939 | 62546955 | 0 |
| 304 | yri1179 | NA19207 | chr9 | 62545375 | 62570383 | 62546937 | 62546952 | 0 |
| 305 | yri1179 | NA19207 | chr9 | 62545375 | 62570383 | 62546937 | 62546955 | 0 |
| 306 | yri1179 | NA19207 | chr9 | 62545375 | 62570383 | 62546937 | 62546955 | 0 |
| 307 | yri1179 | NA19207 | chr9 | 62545375 | 62570383 | 62546938 | 62546956 | 0 |
| 308 | yri1179 | NA19207 | chr9 | 62545375 | 62570383 | 62546938 | 62546956 | 0 |
| 309 | yri1177 | NA19207 | chr9 | 36872141 | 36890817 | 36886672 | 36886707 | 0 |
| 310 | yri1177 | NA19207 | chr9 | 36872141 | 36890817 | 36887606 | 36887634 | 0 |
| 311 | yri1177 | NA19207 | chr9 | 36872141 | 36890817 | 36885950 | 36885967 | 0 |
| 312 | yri1177 | NA19207 | chr9 | 36872141 | 36890817 | 36890043 | 36890075 | 0 |
| 313 | yri1177 | NA19207 | chr9 | 36872141 | 36890817 | 36883462 | 36883484 | 0 |
| 314 | yri1177 | NA19207 | chr9 | 36872141 | 36890817 | 36880788 | 36880823 | 0 |
| 315 | yri1177 | NA19207 | chr9 | 36872141 | 36890817 | 36888356 | 36888384 | 0 |
| 316 | yri1177 | NA19207 | chr9 | 36872141 | 36890817 | 36885950 | 36885968 | 0 |
| 317 | yri1177 | NA19207 | chr9 | 36872141 | 36890817 | 36889724 | 36889753 | 0 |
| 318 | yri1177 | NA19207 | chr9 | 36872141 | 36890817 | 36885951 | 36885969 | 0 |
| 319 | yri1177 | NA19207 | chr9 | 36872141 | 36890817 | 36885949 | 36885970 | 0 |
| 320 | yri1177 | NA19207 | chr9 | 36872141 | 36890817 | 36885949 | 36885970 | 0 |
| 321 | yri1177 | NA19207 | chr9 | 36872141 | 36890817 | 36885950 | 36885968 | 0 |
| 322 | yri1177 | NA19207 | chr9 | 36872141 | 36890817 | 36885950 | 36885967 | 0 |
| 323 | yri1176 | NA19207 | chr8 | 39271742 | 39390862 | 39321673 | 39321685 | 0 |
| 324 | yri1176 | NA19207 | chr8 | 39271742 | 39390862 | 39321673 | 39321685 | 0 |
| 325 | yri1176 | NA19207 | chr8 | 39271742 | 39390862 | 39321673 | 39321685 | 0 |
| 326 | yri1176 | NA19207 | chr8 | 39271742 | 39390862 | 39321673 | 39321685 | 0 |
| 327 | yri1176 | NA19207 | chr8 | 39271742 | 39390862 | 39321673 | 39321685 | 0 |
| 328 | yri1176 | NA19207 | chr8 | 39271742 | 39390862 | 39353584 | 39353596 | 0 |
| 329 | yri1176 | NA19207 | chr8 | 39271742 | 39390862 | 39321673 | 39321685 | 0 |
| 330 | yri1173 | NA19207 | chr4 | 70477074 | 70542965 | 70524381 | 70524400 | 0 |
| 331 | yri1173 | NA19207 | chr4 | 70477074 | 70542965 | 70491429 | 70491441 | 0 |
| 332 | yri1172 | NA19207 | chr3 | 163833596 | 163943569 | 163847884 | 163847897 | 0 |
| 333 | yri542 | NA19098 | chr4 | 34677422 | 34724191 | 34698173 | 34698187 | 0 |
| 334 | yri542 | NA19098 | chr4 | 34677422 | 34724191 | 34698173 | 34698187 | 0 |
| 335 | yri542 | NA19098 | chr4 | 34677422 | 34724191 | 34698173 | 34698187 | 0 |
| 336 | yri542 | NA19098 | chr4 | 34677422 | 34724191 | 34698173 | 34698187 | 0 |
| 337 | yri542 | NA19098 | chr4 | 34677422 | 34724191 | 34698173 | 34698187 | 0 |
| 338 | yri542 | NA19098 | chr4 | 34677422 | 34724191 | 34698173 | 34698187 | 0 |
| 339 | yri542 | NA19098 | chr4 | 34677422 | 34724191 | 34698173 | 34698187 | 0 |
| 340 | yri542 | NA19098 | chr4 | 34677422 | 34724191 | 34698173 | 34698187 | 0 |
| 341 | yri542 | NA19098 | chr4 | 34677422 | 34724191 | 34698173 | 34698187 | 0 |
| 342 | yri542 | NA19098 | chr4 | 34677422 | 34724191 | 34698173 | 34698187 | 0 |
| 343 | yri542 | NA19098 | chr4 | 34677422 | 34724191 | 34698173 | 34698187 | 0 |
| 344 | yri542 | NA19098 | chr4 | 34677422 | 34724191 | 34698173 | 34698187 | 0 |
| 345 | yri542 | NA19098 | chr4 | 34677422 | 34724191 | 34698173 | 34698187 | 0 |
| 346 | yri542 | NA19098 | chr4 | 34677422 | 34724191 | 34698173 | 34698187 | 0 |
| 347 | yri542 | NA19098 | chr4 | 34677422 | 34724191 | 34698173 | 34698187 | 0 |
| 348 | yri542 | NA19098 | chr4 | 34677422 | 34724191 | 34698173 | 34698187 | 0 |
| 349 | yri542 | NA19098 | chr4 | 34677422 | 34724191 | 34698173 | 34698187 | 0 |
| 350 | yri542 | NA19098 | chr4 | 34677422 | 34724191 | 34698173 | 34698187 | 0 |
| 351 | yri542 | NA19098 | chr4 | 34677422 | 34724191 | 34698173 | 34698187 | 0 |
| 352 | yri542 | NA19098 | chr4 | 34677422 | 34724191 | 34698173 | 34698187 | 0 |
| 353 | yri542 | NA19098 | chr4 | 34677422 | 34724191 | 34698173 | 34698187 | 0 |
| 354 | yri542 | NA19098 | chr4 | 34677422 | 34724191 | 34698173 | 34698187 | 0 |
| 355 | yri542 | NA19098 | chr4 | 34677422 | 34724191 | 34698173 | 34698187 | 0 |
| 356 | yri542 | NA19098 | chr4 | 34677422 | 34724191 | 34698173 | 34698187 | 0 |
| 357 | yri542 | NA19098 | chr4 | 34677422 | 34724191 | 34698173 | 34698187 | 0 |
| 358 | yri542 | NA19098 | chr4 | 34677422 | 34724191 | 34698173 | 34698187 | 0 |
| 359 | yri542 | NA19098 | chr4 | 34677422 | 34724191 | 34698173 | 34698187 | 0 |
| 360 | yri542 | NA19098 | chr4 | 34677422 | 34724191 | 34698173 | 34698187 | 0 |
| 361 | yri542 | NA19098 | chr4 | 34677422 | 34724191 | 34698173 | 34698187 | 0 |
| 362 | yri542 | NA19098 | chr4 | 34677422 | 34724191 | 34698173 | 34698187 | 0 |
| 363 | yri542 | NA19098 | chr4 | 34677422 | 34724191 | 34698173 | 34698187 | 0 |
| 364 | yri542 | NA19098 | chr4 | 34677422 | 34724191 | 34698173 | 34698187 | 0 |
| 365 | yri542 | NA19098 | chr4 | 34677422 | 34724191 | 34698173 | 34698187 | 0 |
| 366 | yri542 | NA19098 | chr4 | 34677422 | 34724191 | 34698173 | 34698187 | 0 |
| 367 | yri542 | NA19098 | chr4 | 34677422 | 34724191 | 34698173 | 34698187 | 0 |
| 368 | yri542 | NA19098 | chr4 | 34677422 | 34724191 | 34698173 | 34698187 | 0 |
| 369 | yri542 | NA19098 | chr4 | 34677422 | 34724191 | 34698173 | 34698187 | 0 |
| 370 | yri542 | NA19098 | chr4 | 34677422 | 34724191 | 34698173 | 34698187 | 0 |
| 371 | yri542 | NA19098 | chr4 | 34677422 | 34724191 | 34698173 | 34698187 | 0 |
| 372 | yri542 | NA19098 | chr4 | 34677422 | 34724191 | 34698173 | 34698187 | 0 |
| 373 | yri542 | NA19098 | chr4 | 34677422 | 34724191 | 34698173 | 34698187 | 0 |
| 374 | yri542 | NA19098 | chr4 | 34677422 | 34724191 | 34698173 | 34698187 | 0 |
| 375 | yri542 | NA19098 | chr4 | 34677422 | 34724191 | 34698173 | 34698187 | 0 |
| 376 | yri542 | NA19098 | chr4 | 34677422 | 34724191 | 34698173 | 34698187 | 0 |
| 377 | yri542 | NA19098 | chr4 | 34677422 | 34724191 | 34698173 | 34698187 | 0 |
| 378 | yri542 | NA19098 | chr4 | 34677422 | 34724191 | 34698173 | 34698187 | 0 |
| 379 | yri542 | NA19098 | chr4 | 34677422 | 34724191 | 34698173 | 34698187 | 0 |
| 380 | yri542 | NA19098 | chr4 | 34677422 | 34724191 | 34698173 | 34698187 | 0 |
| 381 | yri542 | NA19098 | chr4 | 34677422 | 34724191 | 34698173 | 34698187 | 0 |
| 382 | yri542 | NA19098 | chr4 | 34677422 | 34724191 | 34698173 | 34698187 | 0 |
| 383 | yri542 | NA19098 | chr4 | 34677422 | 34724191 | 34698173 | 34698187 | 0 |
| 384 | yri542 | NA19098 | chr4 | 34677422 | 34724191 | 34698173 | 34698187 | 0 |
| 385 | yri542 | NA19098 | chr4 | 34677422 | 34724191 | 34698173 | 34698187 | 0 |
| 386 | yri542 | NA19098 | chr4 | 34677422 | 34724191 | 34698173 | 34698187 | 0 |
| 387 | yri542 | NA19098 | chr4 | 34677422 | 34724191 | 34698173 | 34698187 | 0 |
| 388 | yri542 | NA19098 | chr4 | 34677422 | 34724191 | 34698173 | 34698187 | 0 |
| 389 | yri542 | NA19098 | chr4 | 34677422 | 34724191 | 34698173 | 34698187 | 0 |
| 390 | yri543 | NA19098 | chr4 | 69432417 | 69486334 | 69459593 | 69459611 | 0 |
| 391 | yri863 | NA19143 | chr12 | 63304111 | 63323750 | 63321617 | 63321649 | 147 |
| 392 | yri870 | NA19143 | chr4 | 70477074 | 70542965 | 70491312 | 70491335 | 0 |
| 393 | yri870 | NA19143 | chr4 | 70477074 | 70542965 | 70505769 | 70505791 | 0 |
| 394 | yri869 | NA19143 | chr4 | 69482361 | 69491890 | 69490192 | 69490216 | 0 |
| 395 | yri875 | NA19144 | chr15 | 32437866 | 32525037 | 32497574 | 32497601 | 0 |
| 396 | yri875 | NA19144 | chr15 | 32437866 | 32525037 | 32498296 | 32498331 | 0 |
| 397 | yri875 | NA19144 | chr15 | 32437866 | 32525037 | 32498364 | 32498384 | 0 |
| 398 | yri875 | NA19144 | chr15 | 32437866 | 32525037 | 32498130 | 32498165 | 0 |
| 399 | yri875 | NA19144 | chr15 | 32437866 | 32525037 | 32498210 | 32498245 | 0 |
| 400 | yri875 | NA19144 | chr15 | 32437866 | 32525037 | 32498367 | 32498387 | 0 |
| 401 | yri875 | NA19144 | chr15 | 32437866 | 32525037 | 32498032 | 32498057 | 0 |
| 402 | yri875 | NA19144 | chr15 | 32437866 | 32525037 | 32498584 | 32498619 | 0 |
| 403 | yri875 | NA19144 | chr15 | 32437866 | 32525037 | 32498367 | 32498387 | 0 |
| 404 | yri875 | NA19144 | chr15 | 32437866 | 32525037 | 32443256 | 32443269 | 0 |
| 405 | yri875 | NA19144 | chr15 | 32437866 | 32525037 | 32439796 | 32439812 | 0 |
| 406 | yri875 | NA19144 | chr15 | 32437866 | 32525037 | 32498102 | 32498120 | 0 |
| 407 | yri875 | NA19144 | chr15 | 32437866 | 32525037 | 32498367 | 32498387 | 0 |
| 408 | yri875 | NA19144 | chr15 | 32437866 | 32525037 | 32443256 | 32443269 | 0 |
| 409 | yri875 | NA19144 | chr15 | 32437866 | 32525037 | 32443256 | 32443269 | 0 |
| 410 | yri875 | NA19144 | chr15 | 32437866 | 32525037 | 32522674 | 32522685 | 0 |
| 411 | yri875 | NA19144 | chr15 | 32437866 | 32525037 | 32443256 | 32443268 | 0 |
| 412 | yri875 | NA19144 | chr15 | 32437866 | 32525037 | 32443256 | 32443269 | 0 |
| 413 | yri875 | NA19144 | chr15 | 32437866 | 32525037 | 32443257 | 32443269 | 0 |
| 414 | yri875 | NA19144 | chr15 | 32437866 | 32525037 | 32443256 | 32443269 | 0 |
| 415 | yri875 | NA19144 | chr15 | 32437866 | 32525037 | 32498280 | 32498315 | 0 |
| 416 | yri876 | NA19144 | chr22 | 21359787 | 21388825 | 21388131 | 21388145 | 200 |
| 417 | yri778 | NA19137 | chr2 | 89796705 | 90026105 | 90011340 | 90011351 | 0 |
| 418 | yri778 | NA19137 | chr2 | 89796705 | 90026105 | 90011340 | 90011352 | 0 |
| 419 | yri778 | NA19137 | chr2 | 89796705 | 90026105 | 89810564 | 89810582 | 0 |
| 420 | yri778 | NA19137 | chr2 | 89796705 | 90026105 | 89871664 | 89871679 | 0 |
| 421 | yri778 | NA19137 | chr2 | 89796705 | 90026105 | 90011340 | 90011352 | 0 |
| 422 | yri778 | NA19137 | chr2 | 89796705 | 90026105 | 90011340 | 90011352 | 0 |
| 423 | yri778 | NA19137 | chr2 | 89796705 | 90026105 | 89913976 | 89913989 | 0 |
| 424 | yri778 | NA19137 | chr2 | 89796705 | 90026105 | 90011340 | 90011352 | 0 |
| 425 | yri778 | NA19137 | chr2 | 89796705 | 90026105 | 90011340 | 90011352 | 0 |
| 426 | yri778 | NA19137 | chr2 | 89796705 | 90026105 | 89885178 | 89885191 | 0 |
| 427 | yri778 | NA19137 | chr2 | 89796705 | 90026105 | 90011340 | 90011352 | 0 |
| 428 | yri778 | NA19137 | chr2 | 89796705 | 90026105 | 89914293 | 89914305 | 0 |
| 429 | yri778 | NA19137 | chr2 | 89796705 | 90026105 | 89949801 | 89949812 | 0 |
| 430 | yri778 | NA19137 | chr2 | 89796705 | 90026105 | 89922603 | 89922614 | 0 |
| 431 | yri778 | NA19137 | chr2 | 89796705 | 90026105 | 90011341 | 90011352 | 0 |
| 432 | yri778 | NA19137 | chr2 | 89796705 | 90026105 | 90011340 | 90011352 | 0 |
| 433 | yri777 | NA19137 | chr12 | 32414722 | 32422479 | 32415417 | 32415435 | 0 |
| 434 | yri708 | NA19129 | chr4 | 70471691 | 70542965 | 70504679 | 70504692 | 0 |
| 435 | yri729 | NA19130 | chr6 | 103787052 | 103807031 | 103801862 | 103801874 | 0 |
| 436 | yri729 | NA19130 | chr6 | 103787052 | 103807031 | 103801862 | 103801874 | 0 |
| 437 | yri729 | NA19130 | chr6 | 103787052 | 103807031 | 103801862 | 103801874 | 0 |
| 438 | yri729 | NA19130 | chr6 | 103787052 | 103807031 | 103801862 | 103801874 | 0 |
| 439 | yri724 | NA19130 | chr2 | 41213645 | 41220036 | 41219713 | 41219729 | 0 |
| 440 | yri724 | NA19130 | chr2 | 41213645 | 41220036 | 41219714 | 41219728 | 0 |
| 441 | yri724 | NA19130 | chr2 | 41213645 | 41220036 | 41219713 | 41219728 | 0 |
| 442 | yri724 | NA19130 | chr2 | 41213645 | 41220036 | 41219713 | 41219728 | 0 |
| 443 | yri736 | NA19130 | chr8 | 115126252 | 115130784 | 115130401 | 115130413 | 0 |
| 444 | yri736 | NA19130 | chr8 | 115126252 | 115130784 | 115130401 | 115130413 | 0 |
| 445 | yri727 | NA19130 | chr3 | 127035541 | 127042413 | 127040328 | 127040339 | 0 |
| 446 | yri727 | NA19130 | chr3 | 127035541 | 127042413 | 127036259 | 127036273 | 0 |
| 447 | yri725 | NA19130 | chr20 | 1564704 | 1567374 | 1565133 | 1565146 | 0 |
| 448 | yri725 | NA19130 | chr20 | 1564704 | 1567374 | 1564695 | 1564710 | 0 |
| 449 | yri728 | NA19130 | chr4 | 108651560 | 108665451 | 108659511 | 108659530 | 0 |
| 450 | yri126 | NA18505 | chr2 | 89796705 | 90026105 | 89976340 | 89976355 | 0 |
| 451 | yri126 | NA18505 | chr2 | 89796705 | 90026105 | 90011340 | 90011352 | 0 |
| 452 | yri126 | NA18505 | chr2 | 89796705 | 90026105 | 89835187 | 89835202 | 0 |
| 453 | yri126 | NA18505 | chr2 | 89796705 | 90026105 | 89835093 | 89835121 | 0 |
| 454 | yri128 | NA18505 | chr2 | 89826086 | 89981417 | 89976340 | 89976355 | 0 |
| 455 | yri128 | NA18505 | chr2 | 89826086 | 89981417 | 89835187 | 89835202 | 0 |
| 456 | yri128 | NA18505 | chr2 | 89826086 | 89981417 | 89835093 | 89835121 | 0 |
| 457 | yri127 | NA18505 | chr2 | 89803197 | 89848524 | 89835187 | 89835202 | 0 |
| 458 | yri127 | NA18505 | chr2 | 89803197 | 89848524 | 89835093 | 89835121 | 0 |
| 459 | yri125 | NA18505 | chr2 | 71306841 | 71317129 | 71309141 | 71309176 | 0 |
| 460 | yri125 | NA18505 | chr2 | 71306841 | 71317129 | 71309141 | 71309176 | 0 |
| 461 | yri125 | NA18505 | chr2 | 71306841 | 71317129 | 71312657 | 71312676 | 0 |
| 462 | yri133 | NA18505 | chr3 | 46758432 | 46807284 | 46802728 | 46802747 | 0 |
| 463 | yri134 | NA18505 | chr3 | 163833596 | 163943569 | 163862406 | 163862422 | 0 |
| 464 | yri117 | NA18505 | chr1 | 94609885 | 94625063 | 94622194 | 94622212 | 0 |
| 465 | yri1060 | NA19200 | chr4 | 34677422 | 34724191 | 34698173 | 34698187 | 0 |
| 466 | yri1060 | NA19200 | chr4 | 34677422 | 34724191 | 34698173 | 34698187 | 0 |
| 467 | yri1060 | NA19200 | chr4 | 34677422 | 34724191 | 34693530 | 34693545 | 0 |
| 468 | yri1060 | NA19200 | chr4 | 34677422 | 34724191 | 34698173 | 34698187 | 0 |
| 469 | yri1060 | NA19200 | chr4 | 34677422 | 34724191 | 34698173 | 34698187 | 0 |
| 470 | yri1060 | NA19200 | chr4 | 34677422 | 34724191 | 34698173 | 34698187 | 0 |
| 471 | yri1060 | NA19200 | chr4 | 34677422 | 34724191 | 34698173 | 34698187 | 0 |
| 472 | yri1060 | NA19200 | chr4 | 34677422 | 34724191 | 34698173 | 34698187 | 0 |
| 473 | yri1059 | NA19200 | chr3 | 163833596 | 163943569 | 163889451 | 163889472 | 0 |
| 474 | yri1059 | NA19200 | chr3 | 163833596 | 163943569 | 163888487 | 163888505 | 0 |
| 475 | yri1059 | NA19200 | chr3 | 163833596 | 163943569 | 163922670 | 163922687 | 0 |
| 476 | yri1059 | NA19200 | chr3 | 163833596 | 163943569 | 163841085 | 163841097 | 0 |
| 477 | yri1061 | NA19200 | chr4 | 69450972 | 69458490 | 69451026 | 69451050 | 0 |
| 478 | yri1064 | NA19200 | chr7 | 141456537 | 141472285 | 141467479 | 141467494 | 0 |
| 479 | yri1064 | NA19200 | chr7 | 141456537 | 141472285 | 141466343 | 141466359 | 0 |
| 480 | yri1065 | NA19200 | chr7 | 141462154 | 141472285 | 141467479 | 141467494 | 0 |
| 481 | yri1065 | NA19200 | chr7 | 141462154 | 141472285 | 141466343 | 141466359 | 0 |
| 482 | yri1066 | NA19200 | chrX | 91086005 | 91109766 | 91103995 | 91104011 | 0 |
| 483 | yri189 | NA18508 | chr4 | 70471691 | 70542965 | 70472063 | 70472074 | 0 |
| 484 | yri189 | NA18508 | chr4 | 70471691 | 70542965 | 70472063 | 70472074 | 0 |
| 485 | yri548 | NA19099 | chr1 | 146591613 | 146605848 | 146593130 | 146593143 | 0 |
| 486 | yri548 | NA19099 | chr1 | 146591613 | 146605848 | 146593118 | 146593142 | 0 |
| 487 | yri548 | NA19099 | chr1 | 146591613 | 146605848 | 146593110 | 146593142 | 0 |
| 488 | yri548 | NA19099 | chr1 | 146591613 | 146605848 | 146593115 | 146593141 | 0 |
| 489 | yri548 | NA19099 | chr1 | 146591613 | 146605848 | 146593121 | 146593141 | 0 |
| 490 | yri548 | NA19099 | chr1 | 146591613 | 146605848 | 146593116 | 146593138 | 0 |
| 491 | yri548 | NA19099 | chr1 | 146591613 | 146605848 | 146593110 | 146593141 | 0 |
| 492 | yri548 | NA19099 | chr1 | 146591613 | 146605848 | 146593117 | 146593142 | 0 |
| 493 | yri548 | NA19099 | chr1 | 146591613 | 146605848 | 146593116 | 146593142 | 0 |
| 494 | yri548 | NA19099 | chr1 | 146591613 | 146605848 | 146593131 | 146593143 | 0 |
| 495 | yri548 | NA19099 | chr1 | 146591613 | 146605848 | 146593113 | 146593126 | 0 |
| 496 | yri548 | NA19099 | chr1 | 146591613 | 146605848 | 146593148 | 146593171 | 0 |
| 497 | yri548 | NA19099 | chr1 | 146591613 | 146605848 | 146593114 | 146593129 | 0 |
| 498 | yri560 | NA19099 | chr7 | 141456537 | 141472285 | 141470345 | 141470360 | 0 |
| 499 | yri561 | NA19099 | chr7 | 141462154 | 141472285 | 141470345 | 141470360 | 0 |
| 500 | yri742 | NA19131 | chr4 | 34685154 | 34701647 | 34698173 | 34698187 | 0 |
| 501 | yri742 | NA19131 | chr4 | 34685154 | 34701647 | 34698173 | 34698187 | 0 |
| 502 | yri742 | NA19131 | chr4 | 34685154 | 34701647 | 34698173 | 34698187 | 0 |
| 503 | yri742 | NA19131 | chr4 | 34685154 | 34701647 | 34698173 | 34698187 | 0 |
| 504 | yri742 | NA19131 | chr4 | 34685154 | 34701647 | 34698173 | 34698187 | 0 |
| 505 | yri742 | NA19131 | chr4 | 34685154 | 34701647 | 34698173 | 34698187 | 0 |
| 506 | yri742 | NA19131 | chr4 | 34685154 | 34701647 | 34698173 | 34698187 | 0 |
| 507 | yri742 | NA19131 | chr4 | 34685154 | 34701647 | 34698173 | 34698187 | 0 |
| 508 | yri742 | NA19131 | chr4 | 34685154 | 34701647 | 34698173 | 34698187 | 0 |
| 509 | yri742 | NA19131 | chr4 | 34685154 | 34701647 | 34698173 | 34698187 | 0 |
| 510 | yri742 | NA19131 | chr4 | 34685154 | 34701647 | 34698173 | 34698187 | 0 |
| 511 | yri742 | NA19131 | chr4 | 34685154 | 34701647 | 34698173 | 34698187 | 0 |
| 512 | yri742 | NA19131 | chr4 | 34685154 | 34701647 | 34698173 | 34698187 | 0 |
| 513 | yri742 | NA19131 | chr4 | 34685154 | 34701647 | 34698173 | 34698187 | 0 |
| 514 | yri743 | NA19131 | chr4 | 34686467 | 34707485 | 34698173 | 34698187 | 0 |
| 515 | yri743 | NA19131 | chr4 | 34686467 | 34707485 | 34698173 | 34698187 | 0 |
| 516 | yri743 | NA19131 | chr4 | 34686467 | 34707485 | 34698173 | 34698187 | 0 |
| 517 | yri743 | NA19131 | chr4 | 34686467 | 34707485 | 34698173 | 34698187 | 0 |
| 518 | yri743 | NA19131 | chr4 | 34686467 | 34707485 | 34698173 | 34698187 | 0 |
| 519 | yri743 | NA19131 | chr4 | 34686467 | 34707485 | 34698173 | 34698187 | 0 |
| 520 | yri743 | NA19131 | chr4 | 34686467 | 34707485 | 34698173 | 34698187 | 0 |
| 521 | yri743 | NA19131 | chr4 | 34686467 | 34707485 | 34698173 | 34698187 | 0 |
| 522 | yri743 | NA19131 | chr4 | 34686467 | 34707485 | 34698173 | 34698187 | 0 |
| 523 | yri743 | NA19131 | chr4 | 34686467 | 34707485 | 34698173 | 34698187 | 0 |
| 524 | yri743 | NA19131 | chr4 | 34686467 | 34707485 | 34698173 | 34698187 | 0 |
| 525 | yri743 | NA19131 | chr4 | 34686467 | 34707485 | 34698173 | 34698187 | 0 |
| 526 | yri743 | NA19131 | chr4 | 34686467 | 34707485 | 34698173 | 34698187 | 0 |
| 527 | yri743 | NA19131 | chr4 | 34686467 | 34707485 | 34698173 | 34698187 | 0 |
| 528 | yri738 | NA19131 | chr10 | 54723271 | 54798755 | 54770817 | 54770832 | 0 |
| 529 | yri746 | NA19131 | chr6 | 103787052 | 103807031 | 103801862 | 103801874 | 0 |
| 530 | yri746 | NA19131 | chr6 | 103787052 | 103807031 | 103801862 | 103801874 | 0 |
| 531 | yri746 | NA19131 | chr6 | 103787052 | 103807031 | 103801862 | 103801874 | 0 |
| 532 | yri329 | NA18858 | chr4 | 34685154 | 34701647 | 34698173 | 34698187 | 0 |
| 533 | yri329 | NA18858 | chr4 | 34685154 | 34701647 | 34694149 | 34694171 | 0 |
| 534 | yri329 | NA18858 | chr4 | 34685154 | 34701647 | 34698173 | 34698187 | 0 |
| 535 | yri329 | NA18858 | chr4 | 34685154 | 34701647 | 34698173 | 34698187 | 0 |
| 536 | yri329 | NA18858 | chr4 | 34685154 | 34701647 | 34698173 | 34698187 | 0 |
| 537 | yri329 | NA18858 | chr4 | 34685154 | 34701647 | 34698173 | 34698187 | 0 |
| 538 | yri329 | NA18858 | chr4 | 34685154 | 34701647 | 34698173 | 34698187 | 0 |
| 539 | yri329 | NA18858 | chr4 | 34685154 | 34701647 | 34698173 | 34698187 | 0 |
| 540 | yri329 | NA18858 | chr4 | 34685154 | 34701647 | 34698173 | 34698187 | 0 |
| 541 | yri329 | NA18858 | chr4 | 34685154 | 34701647 | 34698173 | 34698187 | 0 |
| 542 | yri329 | NA18858 | chr4 | 34685154 | 34701647 | 34698173 | 34698187 | 0 |
| 543 | yri329 | NA18858 | chr4 | 34685154 | 34701647 | 34698173 | 34698187 | 0 |
| 544 | yri329 | NA18858 | chr4 | 34685154 | 34701647 | 34698173 | 34698187 | 0 |
| 545 | yri329 | NA18858 | chr4 | 34685154 | 34701647 | 34698173 | 34698187 | 0 |
| 546 | yri336 | NA18858 | chr8 | 103010682 | 103011802 | 103011195 | 103011208 | 0 |
| 547 | yri335 | NA18858 | chr8 | 39250107 | 39326538 | 39321673 | 39321685 | 0 |
| 548 | yri642 | NA19119 | chr1 | 149771758 | 149798424 | 149792876 | 149792891 | 0 |
| 549 | yri642 | NA19119 | chr1 | 149771758 | 149798424 | 149798313 | 149798328 | 0 |
| 550 | yri649 | NA19119 | chr4 | 70471691 | 70542965 | 70472063 | 70472074 | 0 |
| 551 | yri649 | NA19119 | chr4 | 70471691 | 70542965 | 70527341 | 70527354 | 0 |
| 552 | yri649 | NA19119 | chr4 | 70471691 | 70542965 | 70472063 | 70472074 | 0 |
| 553 | yri649 | NA19119 | chr4 | 70471691 | 70542965 | 70472063 | 70472074 | 0 |
| 554 | yri649 | NA19119 | chr4 | 70471691 | 70542965 | 70472063 | 70472074 | 0 |
| 555 | yri643 | NA19119 | chr12 | 11400655 | 11434605 | 11429298 | 11429310 | 0 |
| 556 | yri643 | NA19119 | chr12 | 11400655 | 11434605 | 11429298 | 11429310 | 0 |
| 557 | yri509 | NA19093 | chr2 | 18160633 | 18171634 | 18171450 | 18171461 | 0 |
| 558 | yri510 | NA19093 | chr4 | 70471691 | 70542965 | 70472063 | 70472074 | 0 |
| 559 | yri508 | NA19093 | chr2 | 18156325 | 18177354 | 18171450 | 18171461 | 0 |
| 560 | yri916 | NA19153 | chr4 | 70471691 | 70542965 | 70472063 | 70472074 | 0 |
| 561 | yri916 | NA19153 | chr4 | 70471691 | 70542965 | 70514116 | 70514142 | 0 |
| 562 | yri916 | NA19153 | chr4 | 70471691 | 70542965 | 70491429 | 70491442 | 0 |
| 563 | yri916 | NA19153 | chr4 | 70471691 | 70542965 | 70472063 | 70472074 | 0 |
| 564 | yri916 | NA19153 | chr4 | 70471691 | 70542965 | 70472063 | 70472074 | 0 |
| 565 | yri916 | NA19153 | chr4 | 70471691 | 70542965 | 70472063 | 70472074 | 0 |
| 566 | yri916 | NA19153 | chr4 | 70471691 | 70542965 | 70472063 | 70472074 | 0 |
| 567 | yri914 | NA19153 | chr14 | 74338282 | 74350474 | 74338714 | 74338725 | 0 |
| 568 | yri914 | NA19153 | chr14 | 74338282 | 74350474 | 74341635 | 74341667 | 0 |
| 569 | yri914 | NA19153 | chr14 | 74338282 | 74350474 | 74340706 | 74340719 | 0 |
| 570 | yri920 | NA19153 | chrX | 107662335 | 107675738 | 107666582 | 107666617 | 0 |
| 571 | yri920 | NA19153 | chrX | 107662335 | 107675738 | 107667290 | 107667312 | 0 |
| 572 | yri68 | NA18502 | chrX | 65105136 | 65531010 | 65338335 | 65338347 | 0 |
| 573 | yri68 | NA18502 | chrX | 65105136 | 65531010 | 65283186 | 65283221 | 0 |
| 574 | yri68 | NA18502 | chrX | 65105136 | 65531010 | 65133740 | 65133758 | 0 |
| 575 | yri68 | NA18502 | chrX | 65105136 | 65531010 | 65427016 | 65427029 | 0 |
| 576 | yri68 | NA18502 | chrX | 65105136 | 65531010 | 65133735 | 65133762 | 0 |
| 577 | yri68 | NA18502 | chrX | 65105136 | 65531010 | 65133740 | 65133757 | 0 |
| 578 | yri1119 | NA19204 | chr12 | 130253222 | 130299606 | 130282686 | 130282702 | 0 |
| 579 | yri1119 | NA19204 | chr12 | 130253222 | 130299606 | 130282686 | 130282699 | 0 |
| 580 | yri1119 | NA19204 | chr12 | 130253222 | 130299606 | 130268777 | 130268792 | 0 |
| 581 | yri1119 | NA19204 | chr12 | 130253222 | 130299606 | 130282686 | 130282698 | 0 |
| 582 | yri1121 | NA19204 | chr3 | 163833596 | 163943569 | 163921937 | 163921949 | 0 |
| 583 | yri1254 | NA19222 | chr7 | 104193511 | 104201772 | 104199737 | 104199756 | 0 |
| 584 | yri1254 | NA19222 | chr7 | 104193511 | 104201772 | 104197523 | 104197543 | 0 |
| 585 | yri1254 | NA19222 | chr7 | 104193511 | 104201772 | 104196279 | 104196314 | 0 |
| 586 | yri1254 | NA19222 | chr7 | 104193511 | 104201772 | 104196399 | 104196434 | 0 |
| 587 | yri1254 | NA19222 | chr7 | 104193511 | 104201772 | 104196399 | 104196434 | 0 |
| 588 | yri1254 | NA19222 | chr7 | 104193511 | 104201772 | 104195793 | 104195820 | 0 |
| 589 | yri1254 | NA19222 | chr7 | 104193511 | 104201772 | 104195793 | 104195820 | 0 |
| 590 | yri1254 | NA19222 | chr7 | 104193511 | 104201772 | 104196279 | 104196314 | 0 |
| 591 | yri1253 | NA19222 | chr7 | 3136864 | 3171352 | 3154535 | 3154549 | 0 |
| 592 | yri1253 | NA19222 | chr7 | 3136864 | 3171352 | 3154538 | 3154549 | 0 |
| 593 | yri1211 | NA19210 | chr13 | 18261867 | 18268071 | 18264983 | 18265012 | 0 |
| 594 | yri1211 | NA19210 | chr13 | 18261867 | 18268071 | 18264981 | 18265011 | 0 |
| 595 | yri1211 | NA19210 | chr13 | 18261867 | 18268071 | 18264983 | 18265011 | 0 |
| 596 | yri1211 | NA19210 | chr13 | 18261867 | 18268071 | 18264981 | 18265011 | 0 |
| 597 | yri1211 | NA19210 | chr13 | 18261867 | 18268071 | 18264989 | 18265010 | 0 |
| 598 | yri1211 | NA19210 | chr13 | 18261867 | 18268071 | 18262168 | 18262181 | 0 |
| 599 | yri1210 | NA19210 | chr12 | 81459099 | 81470766 | 81470751 | 81470762 | 0 |
| 600 | yri1210 | NA19210 | chr12 | 81459099 | 81470766 | 81470751 | 81470763 | 0 |
| 601 | yri1210 | NA19210 | chr12 | 81459099 | 81470766 | 81470751 | 81470763 | 0 |
| 602 | yri1210 | NA19210 | chr12 | 81459099 | 81470766 | 81470751 | 81470762 | 0 |
| 603 | yri1210 | NA19210 | chr12 | 81459099 | 81470766 | 81470751 | 81470763 | 0 |
| 604 | yri955 | NA19160 | chr4 | 69432417 | 69486334 | 69440918 | 69440953 | 0 |
| 605 | yri955 | NA19160 | chr4 | 69432417 | 69486334 | 69440523 | 69440558 | 0 |
| 606 | yri955 | NA19160 | chr4 | 69432417 | 69486334 | 69440918 | 69440953 | 0 |
| 607 | yri955 | NA19160 | chr4 | 69432417 | 69486334 | 69440918 | 69440953 | 0 |
| 608 | yri955 | NA19160 | chr4 | 69432417 | 69486334 | 69440919 | 69440954 | 0 |
| 609 | yri957 | NA19160 | chr4 | 70471691 | 70542965 | 70523527 | 70523562 | 0 |
| 610 | yri837 | NA19141 | chrX | 15964777 | 15971948 | 15967765 | 15967791 | 0 |
| 611 | yri1072 | NA19201 | chr4 | 70471691 | 70542965 | 70472063 | 70472074 | 0 |
| 612 | yri1072 | NA19201 | chr4 | 70471691 | 70542965 | 70472063 | 70472074 | 0 |
| 613 | yri1072 | NA19201 | chr4 | 70471691 | 70542965 | 70472063 | 70472074 | 0 |
| 614 | yri1067 | NA19201 | chr12 | 130625352 | 130629179 | 130625600 | 130625613 | 0 |
| 615 | yri1067 | NA19201 | chr12 | 130625352 | 130629179 | 130627857 | 130627874 | 0 |
| 616 | yri1070 | NA19201 | chr2 | 203499611 | 203511609 | 203502874 | 203502896 | 0 |
| 617 | yri600 | NA19102 | chr20 | 16562202 | 16580314 | 16571995 | 16572007 | 0 |
| 618 | yri600 | NA19102 | chr20 | 16562202 | 16580314 | 16567328 | 16567340 | 0 |
| 619 | yri787 | NA19138 | chr2 | 71306841 | 71317129 | 71306990 | 71307015 | 0 |
| 620 | yri787 | NA19138 | chr2 | 71306841 | 71317129 | 71308703 | 71308719 | 0 |
| 621 | yri787 | NA19138 | chr2 | 71306841 | 71317129 | 71312397 | 71312411 | 0 |
| 622 | yri788 | NA19138 | chr3 | 163833596 | 163943569 | 163888730 | 163888743 | 0 |
| 623 | yri788 | NA19138 | chr3 | 163833596 | 163943569 | 163856809 | 163856820 | 0 |
| 624 | yri784 | NA19138 | chr1 | 16392736 | 16400201 | 16393182 | 16393200 | 0 |
| 625 | yri784 | NA19138 | chr1 | 16392736 | 16400201 | 16393811 | 16393826 | 0 |
| 626 | yri784 | NA19138 | chr1 | 16392736 | 16400201 | 16393177 | 16393207 | 0 |
| 627 | yri908 | NA19152 | chr3 | 46758432 | 46807284 | 46773943 | 46773964 | 0 |
| 628 | yri908 | NA19152 | chr3 | 46758432 | 46807284 | 46760083 | 46760098 | 0 |
| 629 | yri909 | NA19152 | chr3 | 163833596 | 163943569 | 163888205 | 163888220 | 0 |
| 630 | yri909 | NA19152 | chr3 | 163833596 | 163943569 | 163860781 | 163860795 | 0 |
| 631 | yri910 | NA19152 | chr4 | 9969524 | 9980122 | 9973065 | 9973076 | 0 |
| 632 | yri1008 | NA19172 | chr4 | 69432417 | 69486334 | 69440919 | 69440953 | 0 |
| 633 | yri1008 | NA19172 | chr4 | 69432417 | 69486334 | 69440919 | 69440953 | 0 |
| 634 | yri1008 | NA19172 | chr4 | 69432417 | 69486334 | 69462177 | 69462191 | 0 |
| 635 | yri1008 | NA19172 | chr4 | 69432417 | 69486334 | 69445664 | 69445677 | 0 |
| 636 | yri1007 | NA19172 | chr21 | 9979029 | 10012221 | 9986763 | 9986795 | 0 |
| 637 | yri1007 | NA19172 | chr21 | 9979029 | 10012221 | 9985612 | 9985639 | 0 |
| 638 | yri1010 | NA19172 | chr4 | 69460790 | 69486227 | 69462177 | 69462191 | 0 |
| 639 | yri1263 | NA19223 | chrX | 107662335 | 107675738 | 107668792 | 107668827 | 0 |
| 640 | yri1263 | NA19223 | chrX | 107662335 | 107675738 | 107666564 | 107666599 | 0 |
| 641 | yri1263 | NA19223 | chrX | 107662335 | 107675738 | 107672493 | 107672517 | 0 |
| 642 | yri1263 | NA19223 | chrX | 107662335 | 107675738 | 107672660 | 107672689 | 0 |
| 643 | yri941 | NA19159 | chr9 | 11903287 | 11978036 | 11914669 | 11914691 | 0 |
| 644 | yri943 | NA19159 | chrX | 91086005 | 91109766 | 91095542 | 91095554 | 0 |
| 645 | yri940 | NA19159 | chr4 | 21123929 | 21126700 | 21126126 | 21126143 | 0 |
| 646 | yri366 | NA18861 | chr3 | 163833596 | 163943569 | 163852312 | 163852329 | 0 |
| 647 | yri366 | NA18861 | chr3 | 163833596 | 163943569 | 163867289 | 163867304 | 0 |
| 648 | yri409 | NA18870 | chr7 | 141456537 | 141472285 | 141471160 | 141471174 | 0 |
| 649 | yri410 | NA18870 | chr7 | 141462154 | 141472285 | 141471160 | 141471174 | 0 |
| 650 | yri406 | NA18870 | chr7 | 109002968 | 109011761 | 109005142 | 109005157 | 0 |
| 651 | yri407 | NA18870 | chr7 | 109003350 | 109007346 | 109005142 | 109005157 | 0 |
| 652 | yri498 | NA19092 | chr21 | 9979029 | 10012221 | 9981229 | 9981258 | 0 |
| 653 | yri503 | NA19092 | chr7 | 78439557 | 78445109 | 78440452 | 78440469 | 0 |
| 654 | yri499 | NA19092 | chr4 | 9969524 | 9980122 | 9973065 | 9973076 | 0 |
| 655 | yri501 | NA19092 | chr6 | 33985151 | 33989083 | 33988000 | 33988030 | 0 |
| 656 | yri641 | NA19116 | chr8 | 141973953 | 141975249 | 141975020 | 141975050 | 0 |
| 657 | yri628 | NA19116 | chr3 | 163833596 | 163943569 | 163882567 | 163882597 | 0 |
| 658 | yri630 | NA19116 | chr4 | 138551715 | 138556270 | 138552971 | 138553006 | 0 |
| 659 | yri640 | NA19116 | chr8 | 95515277 | 95528026 | 95522210 | 95522242 | 0 |
| 660 | yri1204 | NA19209 | chr3 | 163833596 | 163943569 | 163908250 | 163908264 | 0 |
| 661 | yri1204 | NA19209 | chr3 | 163833596 | 163943569 | 163908250 | 163908264 | 0 |
| 662 | yri1204 | NA19209 | chr3 | 163833596 | 163943569 | 163925954 | 163925966 | 0 |
| 663 | yri1204 | NA19209 | chr3 | 163833596 | 163943569 | 163861425 | 163861441 | 0 |
| 664 | yri1204 | NA19209 | chr3 | 163833596 | 163943569 | 163861425 | 163861441 | 0 |
| 665 | yri1204 | NA19209 | chr3 | 163833596 | 163943569 | 163861425 | 163861442 | 0 |
| 666 | yri1204 | NA19209 | chr3 | 163833596 | 163943569 | 163883714 | 163883725 | 0 |
| 667 | yri1204 | NA19209 | chr3 | 163833596 | 163943569 | 163892558 | 163892569 | 0 |
| 668 | yri1204 | NA19209 | chr3 | 163833596 | 163943569 | 163924687 | 163924698 | 0 |
| 669 | yri1202 | NA19209 | chr12 | 94301826 | 94311594 | 94307613 | 94307627 | 0 |
| 670 | yri1207 | NA19209 | chr8 | 2242110 | 2250519 | 2245141 | 2245156 | 0 |
| 671 | yri1207 | NA19209 | chr8 | 2242110 | 2250519 | 2246124 | 2246136 | 0 |
| 672 | yri1208 | NA19209 | chr8 | 16212027 | 16216726 | 16215806 | 16215817 | 0 |
| 673 | yri1203 | NA19209 | chr17 | 39893166 | 39898343 | 39896716 | 39896727 | 0 |
| 674 | yri1205 | NA19209 | chr6 | 54698192 | 54707081 | 54700513 | 54700527 | 0 |

**Table S4. Sequences of unique reads mapped to the deletion region from 62545375 to 62570383 on chromosome 9 of YRI sample NA19207**. “The start and end” mean the start and end positions of read sequences. “Uniquely and multi mapping” indicates whether sequences were uniquely mapped to the deletion or multi-mapped to other non-deletion regions, respectively. (+) indicates that the read was mapped to the forward strand of the reference genome. Otherwise the read was mapped to the reverse strand. 46 out of 218 reads mapping to the deletion are unique sequences; 3 unique sequences were mapped to the sense strand and 43 to the antisense strand of the reference genome. 14 out of 46 unique reads were multi-mapped to 197 non-deletion regions, whereas the remaining 32 unique reads were mapped exclusively to the deletion region. The average size of unique reads is 15.29 nt for the multi-mapped reads and 24.88 nt for the uniquely mapped reads.

| Start | End | Sequence | Mapping |
| --- | --- | --- | --- |
| 62546298 | 62546330 | CGGGAGCCGTGGCGCCGAGCCTGTCCACGGGGC | Uniquely |
| 62546548 | 62546570 | GTGACGCTGAATGAATGAATACA | Uniquely (+) |
| 62546846 | 62546863 | TAAAGCCTACGGTACCGC | Uniquely |
| 62546851 | 62546883 | CCTACGGTACCGCTATTACCAGGCGGAATCCCA | Uniquely |
| 62546871 | 62546888 | AGGCGGGATCCCATCCAA | Uniquely |
| 62546872 | 62546898 | GGCGGGATCCCATCCAAGTACTAACCA | Uniquely |
| 62546882 | 62546917 | CATCCAAGTACTAACCAGTCCCGACCCTGCTTAGCT | Uniquely |
| 62546927 | 62546959 | CAGAGGCGAGCGGGCGCGTTCAGGGTGGTGTGG | Uniquely |
| 62546931 | 62546966 | GGCGAGCGGGCGCGTTCAGGGTGGTGTGGCCTAGAC | Uniquely |
| 62546931 | 62546966 | TTCGAGCGGGCGCGTTCAGGGTGGTGTGGCCTAGAC | Uniquely |
| 62546937 | 62546950 | CCGGGCGCGTTCAG | Multi |
| 62546937 | 62546949 | CCGGGCGCGTTCA | Multi |
| 62546937 | 62546957 | CCGGGCGCGTTCAGGGTGGTA | Uniquely |
| 62546937 | 62546954 | CCGGGCGCGTTCAGGGTG | Uniquely |
| 62546937 | 62546951 | CCGGGCGCGTTCAGG | Uniquely |
| 62546937 | 62546956 | CCGGGCGCGTTCAGGGTGGT | Uniquely |
| 62546937 | 62546952 | CCGGGCGCGTTCAGGG | Uniquely |
| 62546937 | 62546955 | CCGGGCGCGTTCAGGGTGG | Uniquely |
| 62546937 | 62546968 | CCGGGCGCGTTCAGGGTGGTGTGGCCGTAGAC | Uniquely |
| 62546937 | 62546953 | CCGGGCGCGTTCAGGGT | Uniquely |
| 62546937 | 62546961 | CCGGGCGCGTTCAGGGTGGTATGGC | Uniquely |
| 62546937 | 62546956 | CCGGGCGCGTTCAGGGTGGC | Uniquely |
| 62546937 | 62546962 | CCGGGCGCGTTCAGGGTGGTATGGCC | Uniquely |
| 62546937 | 62546960 | CCGGGCGCGTTCAGGGTGGTATGG | Uniquely |
| 62546937 | 62546955 | CCGGGCGCTTTCAGGGTGG | Uniquely |
| 62546937 | 62546956 | CCGGGCGTGTTCAGGGTGGT | Uniquely |
| 62546937 | 62546958 | CCGGGCGCGTTCAGGGTGGTAT | Uniquely |
| 62546938 | 62546955 | CGGGCGCGTTCAGGGTGG | Multi |
| 62546938 | 62546954 | CGGGCGCGTTCAGGGTG | Multi |
| 62546938 | 62546953 | CGGGCGCGTTCAGGGT | Multi |
| 62546938 | 62546951 | CGGGCGCGTTCAGG | Multi |
| 62546938 | 62546949 | CGGGCGCGTTCA | Multi |
| 62546938 | 62546956 | CGGGCGCGTTCAGGGTGGT | Multi |
| 62546938 | 62546952 | CGGGCGCGTTCAGGG | Multi |
| 62546938 | 62546950 | CGGGCGCGTTCAG | Multi |
| 62546938 | 62546957 | CGGGCGCGTTCAGGGTGGTT | Uniquely |
| 62546938 | 62546963 | CGGGCGCGTTCAGGGTGGTGTGGCCT | Uniquely |
| 62546939 | 62546954 | GGGCGCGTTCAGGGTG | Multi |
| 62546939 | 62546952 | GGGCGCGTTCAGGG | Multi |
| 62546939 | 62546955 | GGGCGCGTTCAGGGTGG | Multi |
| 62546939 | 62546964 | GGGCGCGTTCAGGGTGGTGTGGCCTA | Uniquely |
| 62546940 | 62546955 | GGCGCGTTCAGGGTGG | Multi |
| 62547011 | 62547043 | AGCCCGCATGACTCCAGGCGTCACCGCCACCCC | Uniquely |
| 62547331 | 62547348 | GCTCAGGACGGGTCGTGG | Uniquely (+) |
| 62555415 | 62555444 | CACACTGTGGCCTGAGGTGCATTGCCCACC | Uniquely (+) |
| 62560599 | 62560634 | TTAGAGTCCTTGAGTCCAGGCCTAGGGTCTTCTTGG | Uniquely |

**Table S5. The genetic property of 30 deletions which overlap gene regions**. “Sample” is the name of a sample which includes a deletion. “Gene” is a list of genes which the deletion overlaps. “Exon/intron” indicates that the deletion overlaps any intron or exon region of the indicated gene(s).

| Sample | Exon/Intron | Gene |
| --- | --- | --- |
| NA12812 | Intron | AF168132 |
| NA07357 | Exon | AY208831, AY208839, AY040777, BX538161, AY208833, AY208830, AY208837, AY208834, AJ565851 |
| NA10851 | Intron | BC036371, AF285120 |
| NA12006 | Exon | BC053859, U61084 |
| NA12154 | Intron | AK095838, AB019493, AB019490, AB019489, AB019491, BC018630, AB007940 |
| NA19200 | Exon | AY253269, AY253271, U88244, AF071019 |
| NA19201 | Exon | AF177272 |
| NA19206 | Intron | BC052985, U25110 |
| NA19207 | Exon | AK128178 |
| NA19207 | Intron | M96944, AY463953, AY463954, AY463955, AY463956, AY463957 |
| NA19207 | Exon | AK130933 |
| NA19223 | Intron | BC052985, U25110 |
| NA18505 | Intron | AF058925, BC039695 |
| NA18508 | Exon | AF177272 |
| NA18858 | Intron | AF251061 |
| NA19092 | Intron | AB014605 |
| NA19093 | Exon | AF177272 |
| NA19093 | Exon | AY253269, AY253271, U88244, AF071019 |
| NA19098 | Intron | AY061855 |
| NA19116 | Intron | L13616 |
| NA19119 | Exon | AF177272 |
| NA19130 | Intron | Y10376 |
| NA19137 | Exon | U90028 |
| NA19138 | Exon | AL035288 |
| NA19138 | Exon | BC020825 |
| NA19143 | Exon | AY217662, AY217663, AK000844 |
| NA19144 | Exon | BC022822, BC030984, U96394, AF035038, BC020233, BC022098, BC012876, L38562, AF035032, BC022823, BC015833, BC064491, BC028090, BC007782, L43092 |
| NA19153 | Exon | AB023215, AL136808, AL133586 |
| NA19153 | Exon | AF177272 |
| NA19159 | Intron | AK096400 |

**Table S6. Enrichment of reads in deletions and adjacent regions**

“[Start, End]” indicates the entire deletion or random region. Each deletion region had a random region of a corresponding size. “[Start, Start+1000 nt][End-1000 nt, End] indicates the 1000 nt regions in each end of the deletion or the random region. “[Start-1000 nt, Start]” – indicates the 1000 nt upstream of the 5’ end of the deletion or the random region. “[End, End+1000 nt]” – indicates the 1000 nt downstream of the 3’ end of the deletion or the random region. Enrichments were calculated as described in Figure 2.

| Number of deletions | Region | Deletion  region | Random  region | Enrichment  score | P-value |
| --- | --- | --- | --- | --- | --- |
| 56 deletions | [Start, End] | 7.48e-04  (0.00124) | 8.33e-05  (0.00013) | 8.98 | 9.86e-14 |
|  | [Start, Start+1000 nt]  [End-1000 nt, End] | 1.69e-03  (0.00262) | 5.35e-05  (0.00018) | 31.50 | 2.20e-16 |
|  | [Start-1000 nt, Start] | 5.53e-04  (0.00211) | 5.35e-05  (0.00023) | 10.33 | 0.03358 |
|  | [End, End+1000 nt] | 1.07e-04  (0.00037) | 1.78e-05  (0.00013) | 6.00 | 0.09478 |

**Table S7. Enrichment of reads at the deletion ends as compared to 300 random regions**

The enrichment score of reads in the end region was calculated as the ratio of the normalized total read count in the end region to the normalized total read count in the non-end region. Then the enrichment score of deletions was compared with the median value of the 300 random enrichment scores. For the significance of the read enrichment in the end region of deletions, a randomization test was performed. The p-value was calculated as the proportion of the random sets with an enrichment score greater than the enrichment score of deletions in the 300 random sets.

| Number of regions | Region | Enrichment | | P-value |
| --- | --- | --- | --- | --- |
|  |  | Deletion | 300 random regions (median) |  |
| 2 mismatches |  |  | |  |
| 229 | 5’-end | 2.88 | 0.77 | 0.1100 |
|  | 3’-end | 5.43 | 0.71 | 0.0667 |
|  | 5’- and 3’-end | 4.15 | 0.93 | 0.0933 |
| 56 | 5’-end | 3.28 | 1.07 | 0.1333 |
|  | 3’-end | 6.20 | 0.95 | 0.0400 |
|  | 5’- and3’-end | 4.74 | 1.10 | 0.0800 |
| 1 mismatch |  |  | |  |
| Number of regions | Region | Enrichment | | P |
|  |  | Deletion | 300 random regions (median) |  |
| 229 | 5’-end | 2.91 | 0.83 | 0.1233 |
|  | 3’-end | 5.49 | 0.77 | 0.0733 |
|  | 5’- and 3’-end | 4.20 | 0.97 | 0.0967 |
| 56 | 5’-end | 3.25 | 1.04 | 0.1300 |
|  | 3’-end | 6.12 | 0.94 | 0.0400 |
|  | 5’- and 3’-end | 4.69 | 1.11 | 0.0800 |
| 0 mismatches |  |  | |  |
| Number of regions | Region | Enrichment | | P |
|  |  | Deletion | 300 random regions (median) |  |
| 229 | 5’-end | 2.97 | 0.94 | 0.1200 |
|  | 3’-end | 5.17 | 0.87 | 0.0700 |
|  | 5’- and 3’-end | 4.07 | 1.08 | 0.0867 |
| 43 | 5’-end | 10.24 | 1.18 | 0.0200 |
|  | 3’-end | 17.82 | 1.11 | 0.0267 |
|  | 5’- and 3’-end | 14.03 | 1.32 | 0.0400 |

**Table S8. Sequence logo of the first 15 nucleotides at the 5’-end of ncRNA sequences**. Sequence logos were drawn by WebLogo at http://weblogo.berkeley.edu/logo.cgi.

| ncRNA Type | Sequence logo |
| --- | --- |
| miRNA-pseudogene | 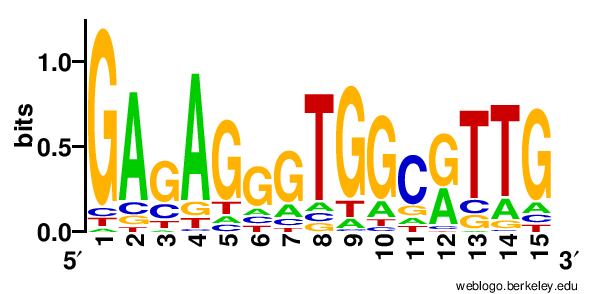 |
| rRNA | 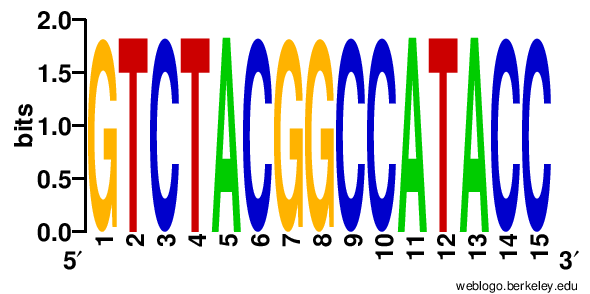 |
| rRNA-pseudogene | 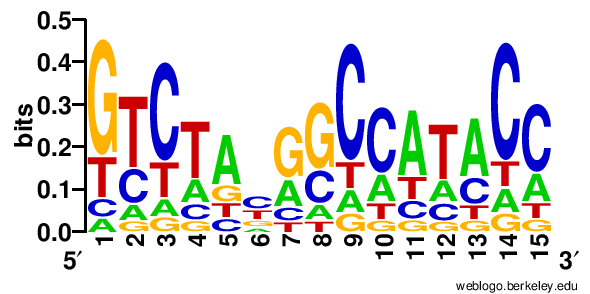 |
| scRNA | 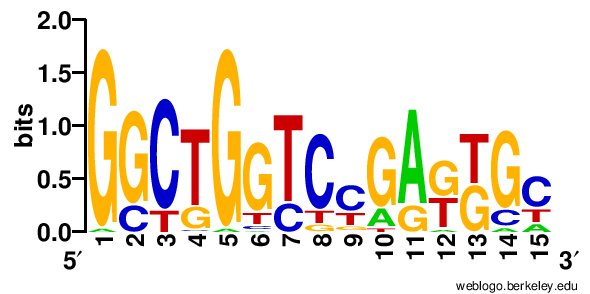 |
| scRNA-pseudogene | 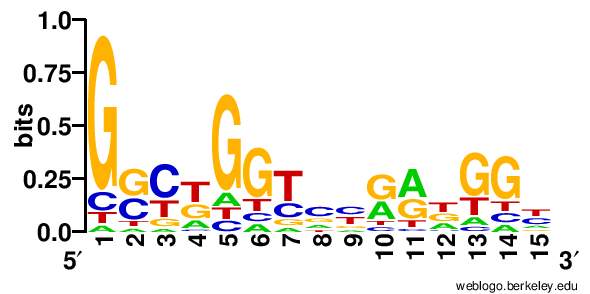 |
| snRNA | 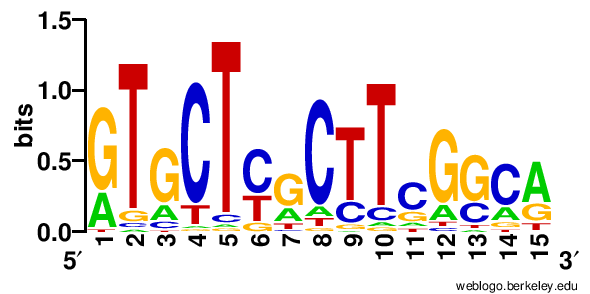 |
| snRNA-pseudogene | 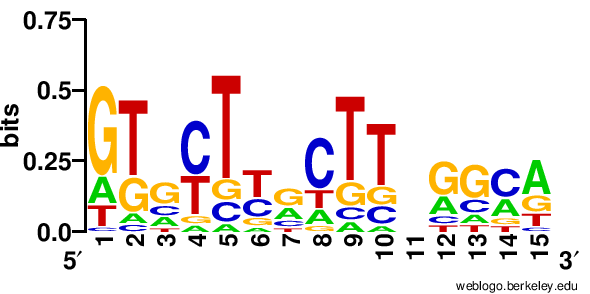 |
| snoRNA | 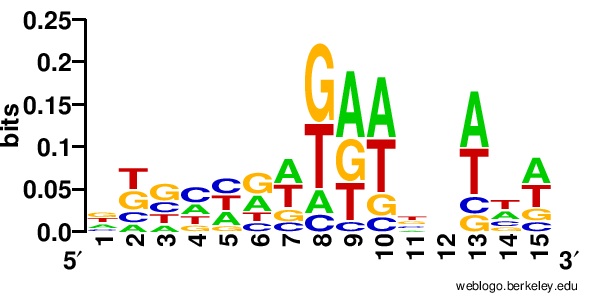 |
| snoRNA-pseudogene | 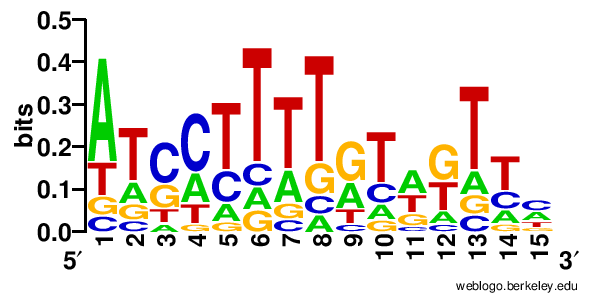 |
| tRNA-pseudogene | 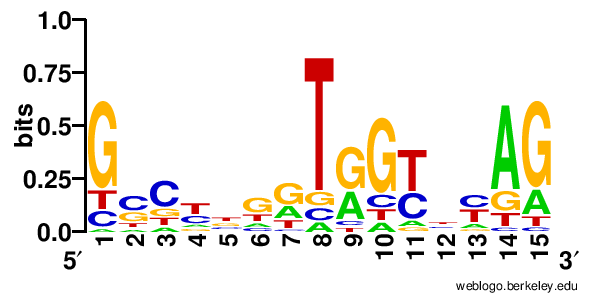 |
| Mt-tRNA-pseudogene | 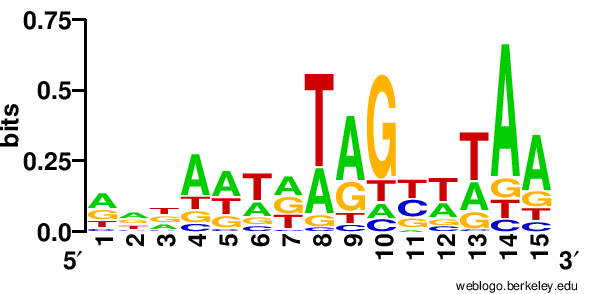 |
| misc_RNA | 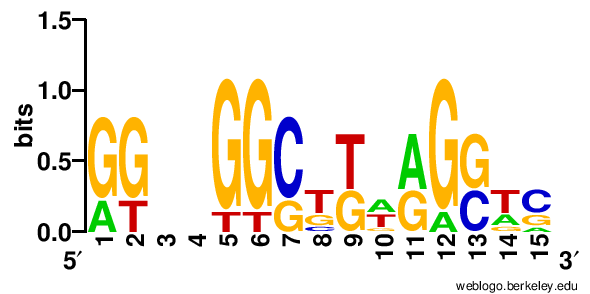 |
| misc_RNA-pseudogene | 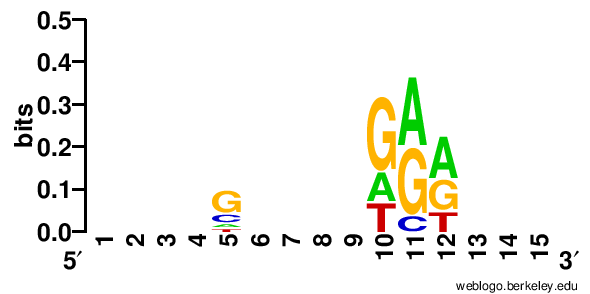 |

**Table S8. Analysis of ncRNA and mRNA read number in samples with deletions and in samples without deletions. Genes inside deletions - all deletions in which genes are entirely inside deletions.**

“Deletion” - deletion catalogue number; “Sample” - sample name; “Chr” - chromosome number; “Start” - start of the deletion; “End” - end of the deletion; “ncRnaRpm” - ncRNA read number (rpm) in the deleted region; “ncRnaRpmNonDel” - ncRNA read number (rpm) in the same region in the samples without a deletion (on average); “mRnaRpm” - mRNA read number (rpm) in the deleted region; “mRnaRpmNonDel”- mRNA read number (rpm) in the same region in the samples without a deletion (on average); “Gene” - name of the gene(s) inside of the deletion; “DavidAnnotation” - link to gene annotation; “DavidUnknown gene” - unknown gene.

| **Deletion** | **Sample** | **Chr** | **Start** | **End** | **ncRnaRpm** | **ncRnaRpm NonDel** | **mRnaRpm** | **mRnaRpm NonDel** | **Gene** | **DavidAnnotation** | **DavidUnknownGene** |
| --- | --- | --- | --- | --- | --- | --- | --- | --- | --- | --- | --- |
| ceu258 | NA07357 | chr22 | 21118175 | 21360293 | **0.209** | **0.312** | **2.339** | 3.0690 | BC040020;BC053901;BC014074;NM_131915;NM_014348;X98922;NM_080842 | [david1\ceu258.PNG](../../../../Users/Igor%20Kovalchuk/AppData/Local/Temp/Temp1_Summary_01262016.zip/Summary_01262016/david1/ceu258.PNG) | NM_131915;NM_014348;NM_080842 |
| ceu5 | NA06985 | chr12 | 131823562 | 131838245 | 0.412 | 0.331 | 0.000 | 0.0005 | AK000762;X52351 | [david1\ceu5.PNG](../../../../Users/Igor%20Kovalchuk/AppData/Local/Temp/Temp1_Summary_01262016.zip/Summary_01262016/david1/ceu5.PNG) | . |
| ceu1451 | NA12873 | chr8 | 39250107 | 39397764 | 0.341 | 0.062 | 0.000 | 0.0071 | AK128178 | . | AK128178 |
| ceu1452 | NA12873 | chr8 | 39268398 | 39389812 | 0.341 | 0.062 | 0.000 | 0.0071 | AK128178 | . | AK128178 |
| ceu464 | NA10851 | chr8 | 39271742 | 39390071 | 0.156 | 0.062 | 0.000 | 0.0071 | AK128178 | . | AK128178 |
| **ceu818** | **NA12005** | **chr8** | **39271742** | **39390071** | **0.092** | **0.062** | **0.038** | **0.0071** | **AK128178** | **.** | **AK128178** |
| ceu660 | NA11831 | chr8 | 39271742 | 39390071 | 0.078 | 0.062 | 0.000 | 0.0071 | AK128178 | . | AK128178 |
|  |  |  |  |  |  |  |  |  |  |  |  |
| ceu1125 | NA12716 | chr8 | 39250107 | 39397764 | 0 | 0.062 | 0.000 | 0.0071 | AK128178 | . | AK128178 |
| ceu1126 | NA12716 | chr8 | 39268398 | 39389812 | 0 | 0.062 | 0.000 | 0.0071 | AK128178 | . | AK128178 |
| ceu832 | NA12006 | chr8 | 39271742 | 39390071 | 0 | 0.062 | 0.000 | 0.0071 | AK128178 | . | AK128178 |
| ceu955 | NA12154 | chr8 | 39250107 | 39397764 | 0 | 0.062 | 0.000 | 0.0071 | AK128178 | . | AK128178 |
| ceu956 | NA12154 | chr8 | 39268398 | 39389812 | 0 | 0.062 | 0.000 | 0.0071 | AK128178 | . | AK128178 |
| yri1176 | NA19207 | chr8 | 39271742 | 39390862 | 0 | 0.017 | 0.000 | 0.0023 | AK128178 | . | AK128178 |
| yri1013 | NA19172 | chr8 | 55414544 | 55423847 | 0 | 0.049 | 0.000 | 0.0006 | AK025905 | [david1\yri1013.PNG](../../../../Users/Igor%20Kovalchuk/AppData/Local/Temp/Temp1_Summary_01262016.zip/Summary_01262016/david1/yri1013.PNG) | . |
| yri126 | NA18505 | chr2 | 89796705 | 90026105 | 0 | 0.028 | 0.000 | 0.0004 | AF035035 | [david1\yri126.PNG](../../../../Users/Igor%20Kovalchuk/AppData/Local/Temp/Temp1_Summary_01262016.zip/Summary_01262016/david1/yri126.PNG) | . |
| yri778 | NA19137 | chr2 | 89796705 | 90026105 | 0 | 0.028 | 0.000 | 0.0004 | AF035035 | [david1\yri778.PNG](../../../../Users/Igor%20Kovalchuk/AppData/Local/Temp/Temp1_Summary_01262016.zip/Summary_01262016/david1/yri778.PNG) | . |
| yri640 | NA19116 | chr8 | 95515277 | 95528026 | 0 | 0.069 | 0.027 | 0.0396 | AF132206 | . | AF132206 |
| yri863 | NA19143 | chr12 | 63304111 | 63323750 | 0 | 0.035 | 0.000 | 0.0028 | AK000844 | [david1\yri863.PNG](../../../../Users/Igor%20Kovalchuk/AppData/Local/Temp/Temp1_Summary_01262016.zip/Summary_01262016/david1/yri863.PNG) | . |
| ceu668 | NA11832 | chr12 | 131823562 | 131838245 | 0 | 0.331 | 0.000 | 0.0005 | AK000762;X52351 | [david1\ceu668.PNG](../../../../Users/Igor%20Kovalchuk/AppData/Local/Temp/Temp1_Summary_01262016.zip/Summary_01262016/david1/ceu668.PNG) | . |
| ceu1119 | NA12716 | chr4 | 69378123 | 69808237 | 0 | 0.546 | 0.048 | 0.4628 | U59209;AF064819;AY359017 | [david1\ceu1119.PNG](../../../../Users/Igor%20Kovalchuk/AppData/Local/Temp/Temp1_Summary_01262016.zip/Summary_01262016/david1/ceu1119.PNG) | . |
| ceu452 | NA10851 | chr4 | 69378123 | 69808237 | 0 | 0.546 | 0.084 | 0.4628 | U59209;AF064819;AY359017 | [david1\ceu452.PNG](../../../../Users/Igor%20Kovalchuk/AppData/Local/Temp/Temp1_Summary_01262016.zip/Summary_01262016/david1/ceu452.PNG) | . |
| ceu63 | NA06994 | chr22 | 21116954 | 21282684 | 0 | 0.247 | 1.608 | 2.7038 | BC040020;BC053901;BC014074 | [david1\ceu63.PNG](../../../../Users/Igor%20Kovalchuk/AppData/Local/Temp/Temp1_Summary_01262016.zip/Summary_01262016/david1/ceu63.PNG) | . |
| ceu64 | NA06994 | chr22 | 21118175 | 21360293 | 0 | 0.312 | 1.954 | 3.0690 | BC040020;BC053901;BC014074;NM_131915;NM_014348;X98922;NM_080842 | [david1\ceu64.PNG](../../../../Users/Igor%20Kovalchuk/AppData/Local/Temp/Temp1_Summary_01262016.zip/Summary_01262016/david1/ceu64.PNG) | NM_014348;NM_080842;NM_131915 |
| ceu856 | NA12044 | chr22 | 21268707 | 21396778 | 0 | 0.060 | 0.035 | 0.3420 | NM_131915;NM_014348;X98922;NM_080842 | [david1\ceu856.PNG](../../../../Users/Igor%20Kovalchuk/AppData/Local/Temp/Temp1_Summary_01262016.zip/Summary_01262016/david1/ceu856.PNG) | NM_131915;NM_080842;NM_014348 |

**Table S9. Analysis of ncRNA and mRNA read number in samples with deletions and in samples without deletions. Genes overlapping deletions – all deletions in which genes partially overlap deletions.**

“Deletion” - deletion catalogue number; “Sample” - sample name; “Chr” - chromosome number; “Start” - start of the deletion; “End” - end of the deletion; “ncRnaRpm” - ncRNA read number (rpm) in the deleted region; “ncRnaRpmNonDel” - ncRNA read number (rpm) in the same region in the samples without a deletion (on average); “mRnaRpm” - mRNA read number (rpm) in the deleted region; “mRnaRpmNonDel”- mRNA read number (rpm) in the same region in the samples without a deletion (on average); “Gene” - name of the gene(s) inside of the deletion; “DavidAnnotation” - link to gene annotation; “DavidUnknown gene” - unknown gene.

| Deletion | Sample | Chr | Start | End | ncRnaRpm | ncRnaRpm NonDel | mRnaRpm | mRnaRpm NonDel | Gene | DavidAnnotation | DavidUnknownGene |
| --- | --- | --- | --- | --- | --- | --- | --- | --- | --- | --- | --- |
| yri916 | NA19153 | chr4 | 70471691 | 70542965 | 1.81 | 0.40 | 0.00 | 0.0000 | AF177272 | [david2\yri916.PNG](../david2/yri916.PNG) | . |
| yri1072 | NA19201 | chr4 | 70471691 | 70542965 | 1.18 | 0.40 | 0.00 | 0.0000 | AF177272 | [david2\yri1072.PNG](../david2/yri1072.PNG) | . |
| yri189 | NA18508 | chr4 | 70471691 | 70542965 | 0.99 | 0.40 | 0.00 | 0.0000 | AF177272 | [david2\yri189.PNG](../david2/yri189.PNG) | . |
| yri649 | NA19119 | chr4 | 70471691 | 70542965 | 0.49 | 0.40 | 0.00 | 0.0000 | AF177272 | [david2\yri649.PNG](../david2/yri649.PNG) | . |
| yri510 | NA19093 | chr4 | 70471691 | 70542965 | 0.16 | 0.40 | 0.00 | 0.0000 | AF177272 | [david2\yri510.PNG](../david2/yri510.PNG) | . |
| yri1263 | NA19223 | chrX | 107662335 | 107675738 | 0.51 | 0.28 | 0.00 | 0.0003 | AF030555 | [david2\yri1263.PNG](../david2/yri1263.PNG) | . |
| yri920 | NA19153 | chrX | 107662335 | 107675738 | 0.36 | 0.28 | **0.02** | 0.0003 | AF030555 | [david2\yri920.PNG](../david2/yri920.PNG) | . |
| ceu1438 | NA12872 | chr9 | 81945705 | 81999325 | 0.64 | 0.13 | 0.00 | 0.0000 | AL133654;AY237536;AY237537;AY237538 | [david2\ceu1438.PNG](../david2/ceu1438.PNG) | . |
| ceu259 | NA07357 | chr22 | 21396778 | 21538141 | 0.63 | 1.96 | 0.00 | 0.0020 | AF035038;AK130519;BC062711;BC018749;BC030983;BC032452;BC033102 | [david2\ceu259.PNG](../david2/ceu259.PNG) | BC062711;AK130519;AF035038 |
| yri837 | NA19141 | chrX | 15964777 | 15971948 | 0.22 | 0.13 | 0.00 | 0.0004 | AK025654;AK024070;BC006256 | [david2\yri837.PNG](../david2/yri837.PNG) | . |
| yri511 | NA19093 | chr6 | 29963788 | 29971727 | 0.16 | 0.03 | 0.00 | 0.0003 | AY253269;AY253271 | [david2\yri511.PNG](../david2/yri511.PNG) | . |
| **yri640** | **NA19116** | **chr8** | **95515277** | **95528026** | **0.13** | **0.15** | **0.47** | **0.6198** | **AB037850;AF116724** | [**david2\yri640.PNG**](../david2/yri640.PNG) | **.** |
| yri876 | NA19144 | chr22 | 21359787 | 21388825 | 1.35 | 3.06 | 0.00 | 0.0145 | BC015833;BC064491;BC028090;BC007782;L43092 | [david2\yri876.PNG](../david2/yri876.PNG) | BC064491;BC015833 |
| ceu1215 | NA12760 | chr2 | 89093935 | 89175498 | 0.79 | 0.01 | 0.00 | 0.0048 | BC034146 | [david2\ceu1215.PNG](../david2/ceu1215.PNG) | . |
| ceu5 | NA06985 | chr12 | 131823562 | 131838245 | 0.62 | 0.22 | 0.00 | 0.0005 | BC046206 | [david2\ceu5.PNG](../david2/ceu5.PNG) | . |
| yri831 | NA19141 | chr12 | 22086469 | 22099211 | 0.45 | 0.15 | 0.00 | 0.0010 | BC016609;AK022927 | [david2\yri831.PNG](../david2/yri831.PNG) | . |
| **ceu824** | **NA12006** | **chr22** | **37615466** | **37624865** | **0.17** | **0.14** | **55.51** | **50.8301** | **BC053859;U61084** | [**david2\ceu824.PNG**](../david2/ceu824.PNG) | **.** |
|  |  |  |  |  | 0.65 | 0.52 |  |  |  |  |  |
|  |  |  |  |  |  |  |  |  |  |  |  |
| ceu856 | NA12044 | chr22 | 21268707 | 21396778 | 0.00 | 2.37 | 2.56 | 2.9955 | U96394;BC020233;AF035032;BC022823;BC015833;BC064491;BC028090;BC007782;L43092 | [david2\ceu856.PNG](../david2/ceu856.PNG) | AF035032;BC064491;BC015833;BC020233 |
| ceu64 | NA06994 | chr22 | 21118175 | 21360293 | 0.00 | 0.15 | 2.30 | 2.8223 | BC030984;U96394;BC020233;L38562;AF035032;BC022823 | [david2\ceu64.PNG](../david2/ceu64.PNG) | AF035032;BC020233 |
| ceu258 | NA07357 | chr22 | 21118175 | 21360293 | 0.00 | 0.15 | 0.91 | 2.8223 | BC030984;U96394;BC020233;L38562;AF035032;BC022823 | [david2\ceu258.PNG](../david2/ceu258.PNG) | BC020233;AF035032 |
| yri787 | NA19138 | chr2 | 71306841 | 71317129 | 0.00 | 0.02 | 0.10 | 0.0179 | BC020825 | [david2\yri787.PNG](../david2/yri787.PNG) | . |
| yri125 | NA18505 | chr2 | 71306841 | 71317129 | 0.00 | 0.02 | 0.07 | 0.0179 | BC020825 | [david2\yri125.PNG](../david2/yri125.PNG) | . |
| ceu273 | NA07357 | chr9 | 32991449 | 33014917 | 0.00 | 0.05 | 0.06 | 0.0245 | AY208831;AY208839;AY040777;BX538161;AY208833;AY208830;AY208837;AY208834;AJ565851 | [david2\ceu273.PNG](../david2/ceu273.PNG) | . |
| yri949 | NA19160 | chr14 | 74338282 | 74350474 | 0.00 | 0.08 | 0.05 | 0.0200 | AB023215;AL136808;AL133586 | [david2\yri949.PNG](../david2/yri949.PNG) | . |
| ceu1356 | NA12814 | chr1 | 142902233 | 142921305 | 0.00 | 0.00 | 0.00 | 0.0007 | AB033071 | [david2\ceu1356.PNG](../david2/ceu1356.PNG) | . |
| ceu1423 | NA12872 | chr2 | 89093935 | 89175498 | 0.00 | 0.01 | 0.00 | 0.0048 | BC034146 | [david2\ceu1423.PNG](../david2/ceu1423.PNG) | . |
| ceu257 | NA07357 | chr21 | 9979029 | 10016793 | 0.00 | 0.00 | 0.00 | 0.0023 | BC028719;AY219887;AY219888;AF007118 | [david2\ceu257.PNG](../david2/ceu257.PNG) | . |
| ceu63 | NA06994 | chr22 | 21116954 | 21282684 | 0.00 | 0.00 | 0.00 | 0.0054 | BC030984;L38562 | [david2\ceu63.PNG](../david2/ceu63.PNG) | . |
| ceu66 | NA06994 | chr22 | 21396778 | 21538141 | 0.00 | 1.96 | 0.00 | 0.0020 | AF035038;AK130519;BC062711;BC018749;BC030983;BC032452;BC033102 | [david2\ceu66.PNG](../david2/ceu66.PNG) | AK130519;AF035038;BC062711 |
| ceu668 | NA11832 | chr12 | 131823562 | 131838245 | 0.00 | 0.22 | 0.00 | 0.0005 | BC046206 | [david2\ceu668.PNG](../david2/ceu668.PNG) | . |
| yri1007 | NA19172 | chr21 | 9979029 | 10012221 | 0.00 | 0.01 | 0.00 | 0.0000 | BC028719;AY219887;AY219888;AF007118 | [david2\yri1007.PNG](../david2/yri1007.PNG) | . |
| yri1008 | NA19172 | chr4 | 69432417 | 69486334 | 0.00 | 0.27 | 0.00 | 0.1717 | U59209 | [david2\yri1008.PNG](../david2/yri1008.PNG) | . |
| yri1056 | NA19200 | chr10 | 46327384 | 46342622 | 0.00 | 0.00 | 0.00 | 0.0185 | AK128634 | [david2\yri1056.PNG](../david2/yri1056.PNG) | . |
| yri1063 | NA19200 | chr6 | 29963788 | 29971727 | 0.00 | 0.03 | 0.00 | 0.0003 | AY253269;AY253271 | [david2\yri1063.PNG](../david2/yri1063.PNG) | . |
| yri1179 | NA19207 | chr9 | 62545375 | 62570383 | 0.00 | 0.02 | 0.00 | 0.0000 | AK130933 | . | AK130933 |
| yri1203 | NA19209 | chr17 | 39893166 | 39898343 | 0.00 | 0.01 | 0.00 | 0.0030 | BC009971 | [david2\yri1203.PNG](../david2/yri1203.PNG) | . |
| yri1254 | NA19222 | chr7 | 104193511 | 104201772 | 0.00 | 0.15 | 0.00 | 0.0021 | AK126439 | No Annotation | . |
| yri133 | NA18505 | chr3 | 46758432 | 46807284 | 0.00 | 0.01 | 0.00 | 0.0000 | AK128390 | [david2\yri133.PNG](../david2/yri133.PNG) | . |
| yri184 | NA18508 | chr12 | 47011045 | 47020876 | 0.00 | 0.04 | 0.00 | 0.0089 | AL713659 | [david2\yri184.PNG](../david2/yri184.PNG) | . |
| yri449 | NA18912 | chr4 | 70471691 | 70542965 | 0.00 | 0.40 | 0.00 | 0.0000 | AF177272 | [david2\yri449.PNG](../david2/yri449.PNG) | . |
| yri708 | NA19129 | chr4 | 70471691 | 70542965 | 0.00 | 0.40 | 0.00 | 0.0000 | AF177272 | [david2\yri708.PNG](../david2/yri708.PNG) | . |
| yri957 | NA19160 | chr4 | 70471691 | 70542965 | 0.00 | 0.40 | 0.00 | 0.0000 | AF177272 | [david2\yri957.PNG](../david2/yri957.PNG) | . |
| yri498 | NA19092 | chr21 | 9979029 | 10012221 | 0.00 | 0.01 | 0.00 | 0.0000 | BC028719;AY219887;AY219888;AF007118 | [david2\yri498.PNG](../david2/yri498.PNG) | . |
| yri543 | NA19098 | chr4 | 69432417 | 69486334 | 0.00 | 0.27 | 0.00 | 0.1717 | U59209 | [david2\yri543.PNG](../david2/yri543.PNG) | . |
| yri57 | NA18502 | chr12 | 11398341 | 11431147 | 0.00 | 0.01 | 0.00 | 0.0080 | BC044827;K03204 | [david2\yri57.PNG](../david2/yri57.PNG) | . |
| yri58 | NA18502 | chr14 | 74338282 | 74350474 | 0.00 | 0.08 | 0.00 | 0.0200 | AB023215;AL136808;AL133586 | [david2\yri58.PNG](../david2/yri58.PNG) | . |
| yri777 | NA19137 | chr12 | 32414722 | 32422479 | 0.00 | 0.01 | 0.00 | 0.0179 | U90028 | [david2\yri777.PNG](../david2/yri777.PNG) | . |
| yri784 | NA19138 | chr1 | 16392736 | 16400201 | 0.00 | 0.00 | 0.00 | 0.0000 | AL035288 | No Annotation | . |
| yri908 | NA19152 | chr3 | 46758432 | 46807284 | 0.00 | 0.01 | 0.00 | 0.0000 | AK128390 | [david2\yri908.PNG](../david2/yri908.PNG) | . |
| yri914 | NA19153 | chr14 | 74338282 | 74350474 | 0.00 | 0.08 | 0.00 | 0.0200 | AB023215;AL136808;AL133586 | [david2\yri914.PNG](../david2/yri914.PNG) | . |
| yri955 | NA19160 | chr4 | 69432417 | 69486334 | 0.00 | 0.27 | 0.00 | 0.1717 | U59209 | [david2\yri955.PNG](../david2/yri955.PNG) | . |
